# Evaluation of the Immune Response of Patulin by Proteomics

**DOI:** 10.3390/bios14070322

**Published:** 2024-06-27

**Authors:** Feng Wang, Lukai Ma, Qin Wang, Bruce D. Hammock, Gengsheng Xiao, Ruijing Liu

**Affiliations:** 1 Guangdong Provincial Key Laboratory of Lingnan Specialty Food Science and Technology, College of Light Industry and Food, Zhongkai University of Agriculture and Engineering, Guangzhou 510225, China; m1991lk@163.com (L.M.); wangqin@zhku.edu.cn (Q.W.); gshxiao@aliyun.com (G.X.); 2 Department of Entomology and Nematology and UCD Comprehensive Cancer Center, University of California, Davis, CA 95616, USA; bdhammock@ucdavis.edu; 3 Guangdong Engineering Technology Research Center for Pre-Cooked Food Processing and Quality Evaluation, Shunde Polytechnic, Foshan 528333, China

**Keywords:** 4D-DIA, proteomics, patulin, immune response, differential proteins

## Abstract

Patulin, an emerging mycotoxin with high toxicity, poses great risks to public health. Considering the poor antibody production in patulin immunization, this study focuses on the four-dimensional data-independent acquisition (4D-DIA) quantitative proteomics to reveal the immune response of patulin in rabbits. The rabbit immunization was performed with the complete developed antigens of patulin, followed by the identification of the immune serum. A total of 554 differential proteins, including 292 up-regulated proteins and 262 down-regulated proteins, were screened; the differential proteins were annotated; and functional enrichment analysis was performed. The differential proteins were associated with the pathways of metabolism, gene information processing, environmental information processing, cellular processes, and organismal systems. The functional enrichment analysis indicated that the immunization procedures mostly resulted in the regulation of biochemical metabolic and signal transduction pathways, including the biosynthesis of amino acid (glycine, serine, and threonine), ascorbate, and aldarate metabolism; fatty acid degradation; and antigen processing and presentation. The 14 key proteins with high connectivity included G1U9T1, B6V9S9, G1SCN8, G1TMS5, G1U9U0, A0A0G2JH20, G1SR03, A0A5F9DAT4, G1SSA2, G1SZ14, G1T670, P30947, P29694, and A0A5F9C804, which were obtained by the analysis of protein–protein interaction networks. This study could provide potential directions for protein interaction and antibody production for food hazards in animal immunization.

## 1. Introduction

As a highly toxic mycotoxin produced by the main *Penicillium* and *Aspergillus* strains, patulin (PAT) has been reported in many kinds of fruits and fruit products [1,2]. The patulin biosynthesis in *Penicillium expansum* required several gene clusters, characterized enzymes, a regulation factor, and transporter genes [3,4]. Some risk assessment studies revealed various contamination levels with a median level of 6.30–8.90 μg/kg and a maximum level of 20.0–78.0 μg/kg in apple juices from four different processing seasons in Shaanxi Province of China; 5.1–87.6 μg/kg in dried figs, longans, and hawthorn products from China; 10.5–43.1 μg/L in organic, conventional, and handcrafted apple juices from Belgium; and 2.8–30.9 μg/L in fruit juices from South Korea [5,6,7,8]. Dietary exposure to PAT could bring great risks to human health, including hepatotoxicity, nephrotoxicity, gastrointestinal alterations, immunotoxicity, long-term carcinogenicity, and other toxic effects [9,10]. Compared with instrument-based detection methods, including gas chromatography, liquid chromatography, and mass spectrometry, the antibody-mediated immunoassays for mycotoxins detection have been proven to be an extremely effective and convenient rapid detection method [11]. The immunoassays have shown a great advantage for the analysis of food hazards, including their low cost, high sensitivity, and high-throughput tests [12]. However, production studies of antibodies against PAT have already been tried several times with poor immune effect, including low titer, low specificity, and low sensitivity, and the unsatisfactory immune response in animal immunization has no clear elaboration in these reports [13,14,15,16,17]. The instability and nucleophilic addition reaction of patulin resulted in the low efficiency of immunogen preparation [18,19,20]. Thus, the proteomic investigation of animal immune serum is of great significance for further understanding the immune response.

Proteomics is an emerging technology that is helpful in elaborating the composition, function, and interaction relationships of proteins [21]. Proteomic studies have been performed in the analysis of food-specific proteins and food allergens, food authentication, and the functional evaluation of active food components [22,23]. Furthermore, serum proteomics served as an efficient tool to evaluate the immune response after immunization. Proteomics with the utilization of tandem mass tag (TMT), data-dependent acquisition (DDA), data-independent acquisition (DIA), and four-dimensional data-independent acquisition (4D-DIA) were developed for omics technology [24,25]. VanDuijn et al. utilized recombinant antigens to immunize Wistar rats and then characterized the immune repertoires in groups of rats after immunization using label-free quantitation proteomics and next-generation sequencing. The proteomic results showed that the sharing motifs were present with the clustering of antigen-specific subsets in the immune repertoire [26]. Boutz et al. reported the proteomic identification of monoclonal antibodies from the immune serum of New Zealand white rabbits with the immunization of *Concholepas concholepas* hemocyanin [27]. However, serum proteomics had several challenges associated with the complex diversity of proteins and protein–protein interaction system [28]. Furthermore, 4D-DIA proteomics is a promising technology with high detection efficiency and analytical sensitivity, especially in the analysis of low abundance proteins in serum. The proteomic perspective was explored in the intestinal barrier protective study of jujube peel polyphenols/zein complexes in Zhu’s study, which indicated that the activated lysosome pathway could regulate the immune responses and lipid transportation in the combined Caco-2 Cell and *Caenorhabditis elegans* model to improve the barrier function through 4D-DIA proteomic analysis [29].

In this study, 4D-DIA quantitative proteomics was used to investigate the immune response of the patulin mycotoxin in rabbits, with the key procedures of the preparation of patulin complete antigens, the evaluation of immunization, and serum proteomics. After the immunization of New Zealand White rabbits with patulin antigen, the serum proteomics was investigated by gene ontology (GO), eukaryotic orthologous groups (KOG), and the Kyoto Encyclopedia of Genes and Genomes (KEGG), and the differential proteins in rabbit serum and the protein–protein interaction networks were explored by omics technologies, including proteomic data analysis, functional annotation, functional enrichment analysis, weighted protein co-expression network, and protein–protein interaction networks.

## 2. Materials and Methods

### 2.1. Reagents, Materials, and Apparatus

PAT mycotoxin was obtained with a purity of 98% from J&K Chemical Ltd. (Shanghai, China). Bovine serum albumin (BSA), ovalbumin (OVA), Freund’s complete adjuvants, and Freund’s incomplete adjuvants were purchased from Sigma (Shanghai, China). N,N′-carbonyldiimidazole, 4-dimethylaminopyridine, N,N-dimethyl-formamide, urea, and dithiothreitol were purchased from Aladdin Chemical Technology Co., Ltd. (Shanghai, China). The goat anti-rabbit antibodies labeled with horseradish peroxidase (anti-rabbit IgG-HRP, Catalog number: HS101-01) and prestained protein marker were purchased from Beijing Transgen Biotech Co., Ltd. (Beijing, China). TMB two-component substrate solutions of enzyme-linked immunosorbent assay (ELISA) (Catalog number: PR1210) were obtained from Beijing Solarbio Science & Technology Co., Ltd. (Beijing, China). The ProteoMiner™ Protein Enrichment Small-Capacity Kit (Catalog number: 1633006) was from Bio-Rad Laboratories (Shanghai) Co., Ltd. (Shanghai, China). The trypsin with sequencing grade (Catalog number: V5111) was from Promega Corporation (Madison, WI, USA). Other chemical reagents were purchased from Shanghai Yuanye Biological Technology Co. (Shanghai, China). The MALDI-TOF-MS analysis was performed on the analysis on a Bruker Microflex LRF instrument (Bruker, Billerica, MA, USA). The absorbance values were measured on a Tecan Infinte 200 Pro microplate reader (Mannedorf, Switzerland). LC-MS/MS analysis was performed on a NanoElute UHPLC system coupled to timsTOF Pro (Bruker Daltonics, Billerica, MA, USA).

### 2.2. Synthesis and Identification of Complete Antigens for Patulin

The patulin mycotoxin was a small molecule with a molecular weight of 154.12 below 1000 daltons, which almost had no immunogenicity in animal immunization. Therefore, the complete antigens for patulin were synthesized as described before [30]. More specifically, 2.4 mg patulin, 12.6 mg N,N’-carbonyldiimidazole, and 9.5 mg 4-dimethylaminopyridine were dissolved in 500 μL N,N-dimethyl-formamide and stirred at 4 °C overnight for activation reactions. The supernatant of the mixture was collected after centrifugation at 10,000 rpm, and it was gradually dropped into the solution of a carrier protein (10 mg BSA and 7 mg OVA) dissolved in 6 mL of 0.01 M PBS buffer. The coupling reaction was conducted at 4 °C overnight, and the mixture was dialyzed in 0.01 M PBS buffer for 3 d. The obtained complete antigens were defined as PAT-BSA and PAT-OVA for the target mycotoxin coupled to two kinds of carrier proteins. The complete antigens were analyzed by sodium dodecyl sulphate-polyacrylamide gel electrophoresis (SDS-PAGE).

### 2.3. Immunization of New Zealand White Rabbits with the Complete Antigen PAT-BSA

Female healthy New Zealand white rabbits were selected for multiple immunizations with the complete antigen PAT-BSA. The first immunization injection was conducted with the emulsified reagent of 0.5 mg PAT-BSA with the same volume of Freund’s complete adjuvant. The following booster immunization was performed at intervals of 2 weeks with the equivalent quantity of PAT-BSA emulsified with Freund’s incomplete adjuvant. After five immunizations, all the blood of the rabbits was collected 7 days after the last injection to separate and obtain the serum, and the rabbit serum was stored at −80 °C for further use.

### 2.4. Analysis of Serum Response of Patulin Immunization by ELISA

The obtained serum of rabbits was analyzed by ELISA using the coating antigens PAT-OVA. Briefly, 1.0 mg/L coating antigens in carbonate buffer (0.05 mol/L, pH 9.6) was added in polystyrene microplates with 100 μL/well, and the plates were further incubated in a 37 °C water bath overnight. After 2 times of washing with phosphate-buffered saline with 0.1% Tween 20 (PBST), 5% skimmed milk in PBS solution was added into the microplate wells with 300 μL/well for 3 h incubation with water bath at 37 °C, followed by a drying step in an oven at 37 °C for 1 h. The diluted serum was added into the microplate wells at 100 μL/well and incubated at 37 °C for 40 min. The goat anti-rabbit IgG-HRP (1:5000 diluted in PBST) was then added to each well after the consecutive washing step. The mixture was further incubated at 37 °C for 30 min, followed by another 5-time washing. The fresh substrate solution was added at 100 μL/well to perform another 10 min incubation at 37 °C. The enzymatic reaction was stopped with 2 mol/L H_2_SO_4_ (50 μL/well) after 10 min incubation. The absorption values were measured at a wavelength of 450 nm with a microplate reader.

### 2.5. Protein Extraction, Digestion, and Cleanup of Rabbit Serum

ProteoMiner™ Protein Enrichment Small-Capacity Kit from BioRad (Hercules, CA, USA) was used to perform the extraction, low abundant protein enrichment, and high abundant protein removal in one step. Specifically, a total of 200 μL rabbit serum was first performed with protein extraction, with a protein enrichment small-capacity kit after the removal of high-abundance proteins, and the concentration of the collected eluent protein was measured by bicinchoninic acid (BCA) protein quantitation assay. The 100 μg target serum protein was mixed with 160 μL of 8 M urea, reduced for 45 min at 37 °C with 10 mM dithiothreitol solution, and alkylated for 15 min with 50 mM iodoacetamide at room temperature in the dark. The mixture solution was conducted by adding a 4-fold volume of precooled acetone, and the protein was precipitated at −20 °C for 2 h. The protein precipitate was collected after centrifugation at 15,000 rpm at 4 °C, and it was completely redissolved in 200 μL of 25 mM ammonium bicarbonate solution. The protein solution was added to 3 μL of trypsin (mass ratio of trypsin:protein = 1:50) for overnight digestion at 37 °C. The digested peptides were desalted and concentrated by vacuum centrifugation and redissolved in 0.1% (*v*/*v*) formic acid for proteomic analysis.

### 2.6. 4D-DIA Quantitative Proteomic Analysis of Rabbit Serum

The 4D-DIA quantitative proteomic analysis of rabbit serum was performed by LC-MS/MS. Briefly, the LC procedure was conducted with a reverse-phase C18 column (25 cm × 75 μm, 1.6 μm particle size, IonOpticks, Fitzroy, Australia) on a nanoElute UHPLC system. The 200 ng peptides were separated within 60 min at 50 °C. The mobile phases were composed of phase A (0.1% formic acid) and phase B (0.1% formic acid in acetonitrile) at a flow rate of 0.3 µL/min as follows: 0 min, 2% B; 0–45 min, 2–22% B; 45–50 min, 22–35% B; 50–55 min, 35–80% B; and 55–60 min, 80% B. The peptides were determined with the LC system coupled online to a hybrid timsTOF Pro2 via a CaptiveSpray nano-electrospray ion source (CSI) (Bruker Daltonics, Billerica, MA, USA) in positive mode. The capillary voltage was set to 1500 V, and the 0.7–1.4 Vs/cm^2^ was used for the ion mobility range (1/K0). The MS and MS/MS spectra were acquired from 100 to 1700 *m*/*z*. The TIMS accumulation and ramp time were both set to 100 ms for an ion utilization rate close to 100%. Proteome Discoverer software was used to search and match the spectra of the Uniprot protein database. The 4D-DIA proteomic data were analyzed using DIA-NN (v1.8.1) with the library-free method with the aid of deep learning neural network algorithms. A spectral library from DIA data was created for proteomic quantitative analysis, and the filtered data were used for proteomic bioinformatics analysis.

### 2.7. Proteomic Bioinformatics Analysis

The obtained 4D-DIA proteomic data were identified in the UniProt data resource, and the principal component analysis (PCA), volcano plot analysis, and cluster analysis were performed on the Metware cloud platform (https://cloud.metware.cn/, accessed on 17 April 2023). The functional annotations of differential serum proteins were conducted on the databases of GO (http://geneontology.org/, accessed on 19 April 2023), KOG (http://www.ncbi.nlm.nih.gov/COG, accessed on 22 April 2023), and KEGG (https://www.genome.jp/kegg/, accessed on 25 April 2023). The KEGG pathway enrichment was obtained by the KEGG database. The protein–protein interaction was analyzed on the StringDB databases (http://string-db.org/, accessed on 28 April 2023) and the interaction network was created using Cytoscape 3.10.0 software.

## 3. Results and Discussion

### 3.1. Synthesis and Analysis of Complete Antigens for Patulin

**Figure 1:**
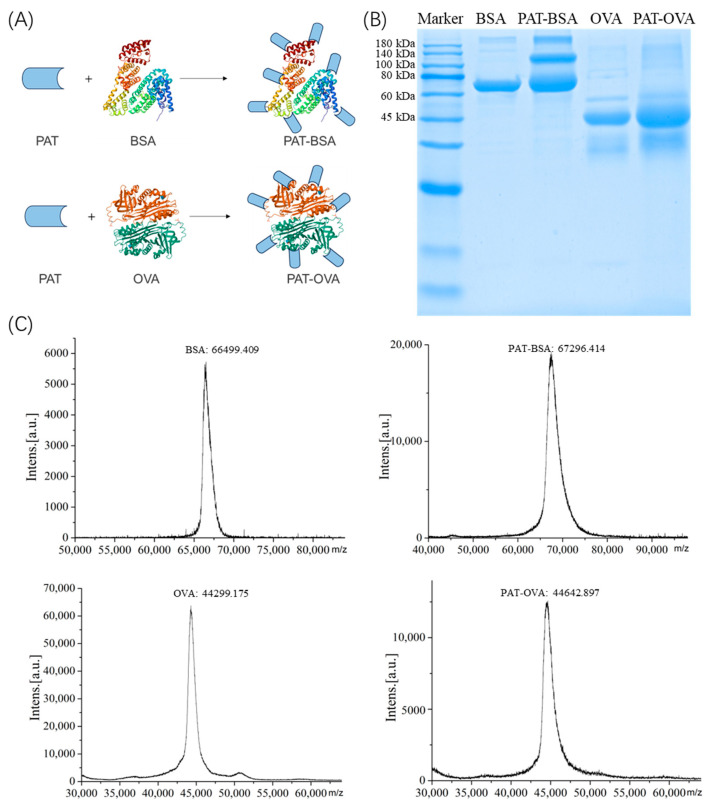
Synthesis diagram (**A**), SDS-PAGE (**B**), and MALDI-TOF-MS (**C**) of the complete antigens of PAT-BSA and PAT-OVA.

In this study, two kinds of complete antigens PAT-BSA and PAT-OVA were prepared using the N,N′-carbonyl diimidazole mediated method as described before [30] (Figure 1A). SDS-PAGE was performed to confirm the successful conjugation of PAT-BSA and PAT-OVA. As shown in Figure 1B, both of the carrier proteins of BSA and OVA and the complete antigens PAT-BSA, PAT-OVA could be performed with different properties. It was indicated that BSA was observed with a molecular weight of ~66 kDa, and OVA was observed with a molecular weight of ~45 kDa. The complete antigens PAT-BSA and PAT-OVA with different coupling ratios presented a larger molecular weight than that of BSA or OVA, which resulted from the coupled reaction between the PAT toxin and the two carrier proteins with similar results to the previous study [31]. The MALDI-TOF-MS analysis of complete antigens of PAT-BSA and PAT-OVA in Figure 1C also showed a successful conjugation. The nominal conjugation rate of PAT to BSA was about 5.2:1, and that of PAT to OVA was about 2.2:1. However, the relatively low coupling rate may be due to the instability and nucleophilic addition reaction of patulin in the coupling reaction [18,19,20]. The successful coupling of PAT conjugates had the characteristics of PAT for further study.

### 3.2. Analysis of Serum Response of Patulin Immunization by ELISA

**Figure 2:**
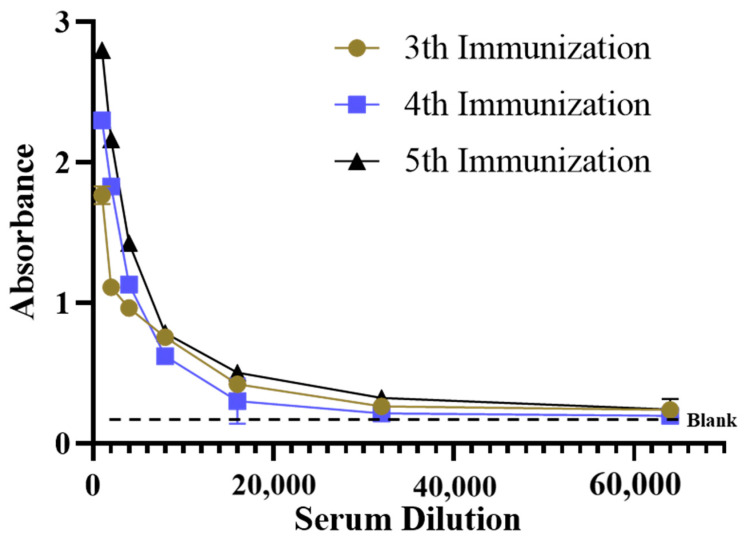
Serum antibody titer analysis with different immunizations.

The complete antigen PAT-BSA was selected as the immunogen for rabbit immunization to improve the immunogenicity of patulin. After five immunizations, the response of PAT immunization in rabbits reached a plateau. The affinity of antiserum is shown in Figure 2, which suggests that multiple immunizations led to the production of specific proteins against PAT with a titer of 1:16,000 (the absorbance at 450 nm about 1.0) in rabbits. The inhibitory rate of PAT was ~20% with an ELISA competitive assay, which may be due to the relatively low coupling rate of the PAT-BSA immunogen. The specific antibodies account for a certain proportion of the serum, which was similar to the previous studies of PAT immunization [13,14,15,16,17].

### 3.3. Differential Expression Protein Analysis in Rabbit Serum

**Figure 3:**
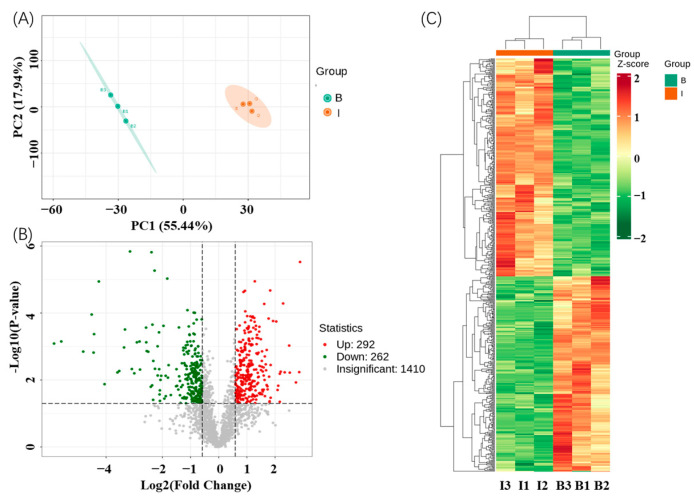
PCA plot (**A**), volcano plot (**B**), and cluster analysis (**C**) of the serums before and after rabbit immunization. The B and I labels refer the blank serum and immune serum, respectively.

After five rabbit immunizations, the final serum of rabbits was collected for proteomic studies. The pre-immune serum from these same rabbits were considered as the proteomic control. All the samples were performed with protein extraction, digestion, cleanup, LC-MS/MS analysis, and finally database search and bioinformatics analysis. The identified proteins in the immune serum of rabbits are listed in Table S1. A total of 1964 unique proteins and 19,080 unique peptides were obtained by 4D-DIA proteomic analysis. Most peptide segments were distributed with 7–20 amino acids, which could follow the general pattern based on enzymatic hydrolysis of trypsin and mass spectrometry fragmentation [32]. Principal component analysis is shown in Figure 3A, which indicates obvious differences in the distribution of protein abundance among different group samples. The parameters of *p* < 0.05 and the fold change (FC) values (FC < 0.6667 or FC > 1.5) were considered as the screening indicators for differential proteins, and the results of differential expression analysis revealed a sum of 554 differential proteins (292 up-regulated proteins and 262 down-regulated proteins) in immunized rabbits compared with the unimmunized rabbits (Figure 3B). The differential proteins in different groups can obviously be observed from the cluster heat maps in Figure 3C. Thus, the immunization procedures had a significant impact on the distribution of serum proteins, and they could up-regulate or down-regulate some key proteins through the immune system and other related systems in rabbits [26,27].

### 3.4. Functional Annotation of Differential Proteins

**Figure 4:**
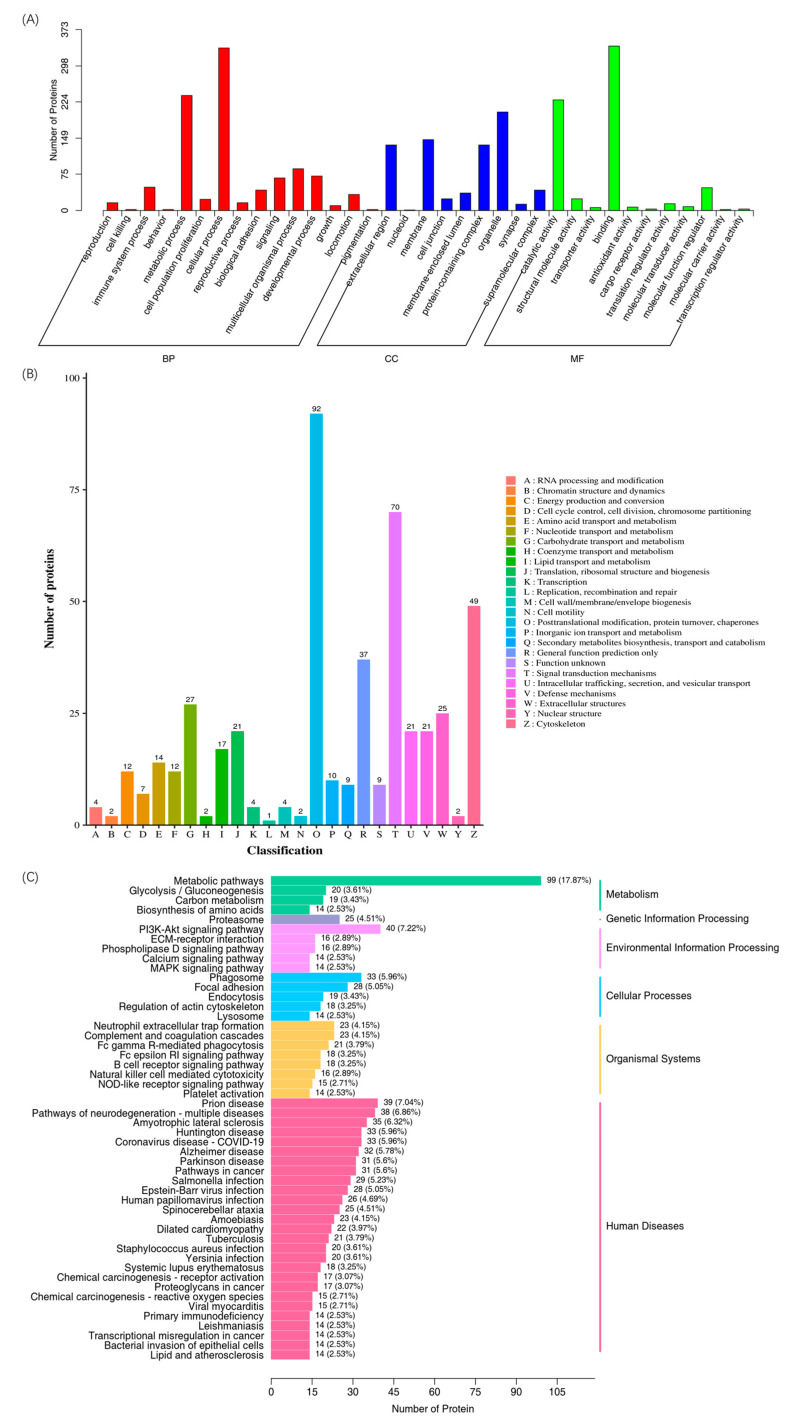
Functional annotations ((**A**): GO; (**B**): KOG; and (**C**): KEGG) of differential proteins.

**Figure 5:**
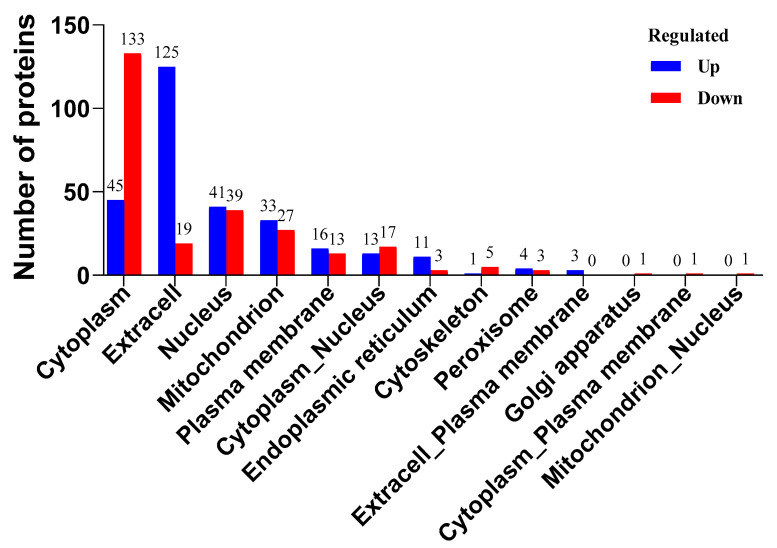
Subcellular localization analysis of differential serum proteins.

All the differential proteins in the rabbit serum were annotated by gene ontology (GO) and were classified into three different categories, including biological processes (BPs), cellular components (CCs), and molecular functions (MFs). As shown in Figure 4A, a total of 1778, 735, and 681 differential proteins belonged to the BP, CC, and MF categories, respectively. The main differential proteins in BP were highly correlated with metabolic process and cellular process, that of CC had a potential relationship with organelles, and that of MF was mainly related to the catalytic activity and binging function. The annotation of eukaryotic orthologous groups (KOG) also showed that the main differential proteins were closely related to the function of posttranslational modification, protein turnover, chaperones, and the function of signal transduction mechanisms and the cytoskeleton (Figure 4B). The functional annotation of GO and KOG clearly illustrated that the PAT immunization had a significant impact on the immune system and related responses in rabbits. Due to the complex response of the immune system and the diversification of antibody production in rabbits, the immunization of PAT antigens could stimulate the rabbits to produce an immune response and lead to the generation of specific antibodies against the PAT mycotoxin [33,34]. The proteins in organisms could coordinate with each other to exercise their biological functions. Thus, the Kyoto Encyclopedia of Genes and Genomes (KEGG) was used to evaluate the relationships of these differential proteins in rabbit serum. As shown in Figure 4C, the differential proteins were relevant to the pathways of metabolism, gene information processing, environmental information processing, cellular processes, and organismal systems. The highest number of differential proteins with a sum of 99 kinds was attributed to the metabolism pathways. Biological cells are a highly ordered structure, and different proteins are synthesized in ribosomes and transported to specific parts to perform biological functions. Therefore, the understanding of the subcellular localization information of differential proteins had great significance [35,36]. Subcellular localization analysis of differential proteins was performed and is shown in Figure 5. A total of 178, 144, 80, and 60 proteins were located in cytoplasm, extracell, nucleus, and mitochondrion, respectively. These four kinds of proteins accounted for 84.4% of the differential proteins, which could greatly affect the physiological function of cells. In particular, 133 proteins located in the cytoplasm were down-regulated and 125 proteins located in extracell were up-regulated. Thus, immunization with PAT could lead to significant changes in the metabolic system and cellular system in response to the mycotoxin stimulation in rabbits.

### 3.5. Functional Enrichment Analysis of Differential Proteins by GO, KOG, and KEGG

**Figure 6:**
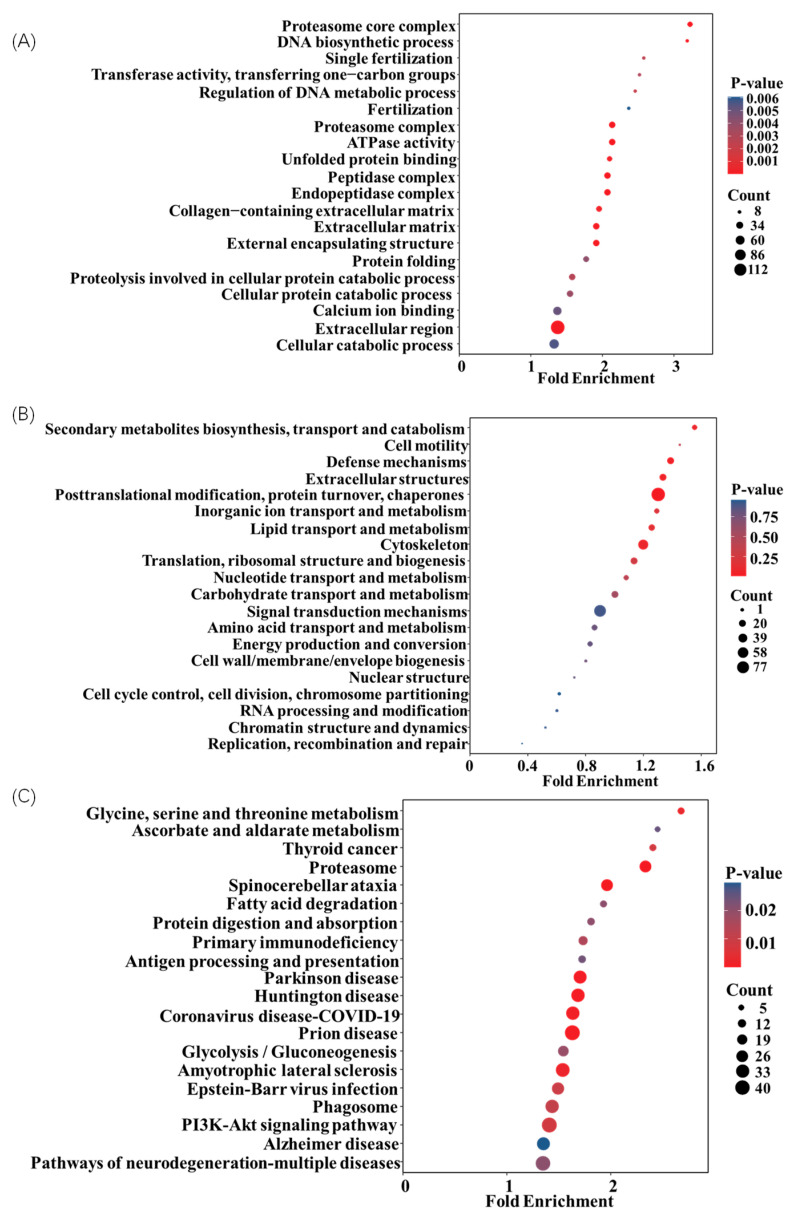
Functional enrichment analysis ((**A**): GO, (**B**): KOG, and (**C**): KEGG) of differential serum proteins.

As shown in Figure 6, the functional enrichment analysis of differential proteins was performed by GO, KOG, and KEGG. The GO enrichment analysis could effectively determine the significant correlation between differential proteins and specific biological functions. As shown by the results in Figure 6A, these differential proteins were involved in BP, CC, and MF, which were highly correlated with the functions of the catabolic process, organic substance catabolic process, cellular catabolic process, extracellular region, and extracellular region space. A further study of the top 20 GO terms in *p*-value ranking indicated that the immunization procedures could affect the immune response with the changes in the proteasome core complex, DNA biosynthetic process, and other 18 kinds of functions. Similar results were obtained with KOG enrichment analysis in Figure 6B, which were involved in secondary metabolite biosynthesis, transport, and catabolism and 19 other kinds of functions. Finally, the KEGG analysis shown in Figure 6C indicated that the immunization was most likely driven by modulating the biosynthesis of amino acids (glycine, serine, and threonine), ascorbate and aldarate metabolism, fatty acid degradation, and antigen processing and presentation. Thus, the procedures of rabbit immunization could be involved in the main biochemical metabolic and signal transduction pathways in differential expression protein to regulate the immune response and immunoglobulin repertoire’s characteristics [37,38,39].

### 3.6. Weighted Protein Co-Expression Network Analysis of Differential Proteins

**Figure 7:**
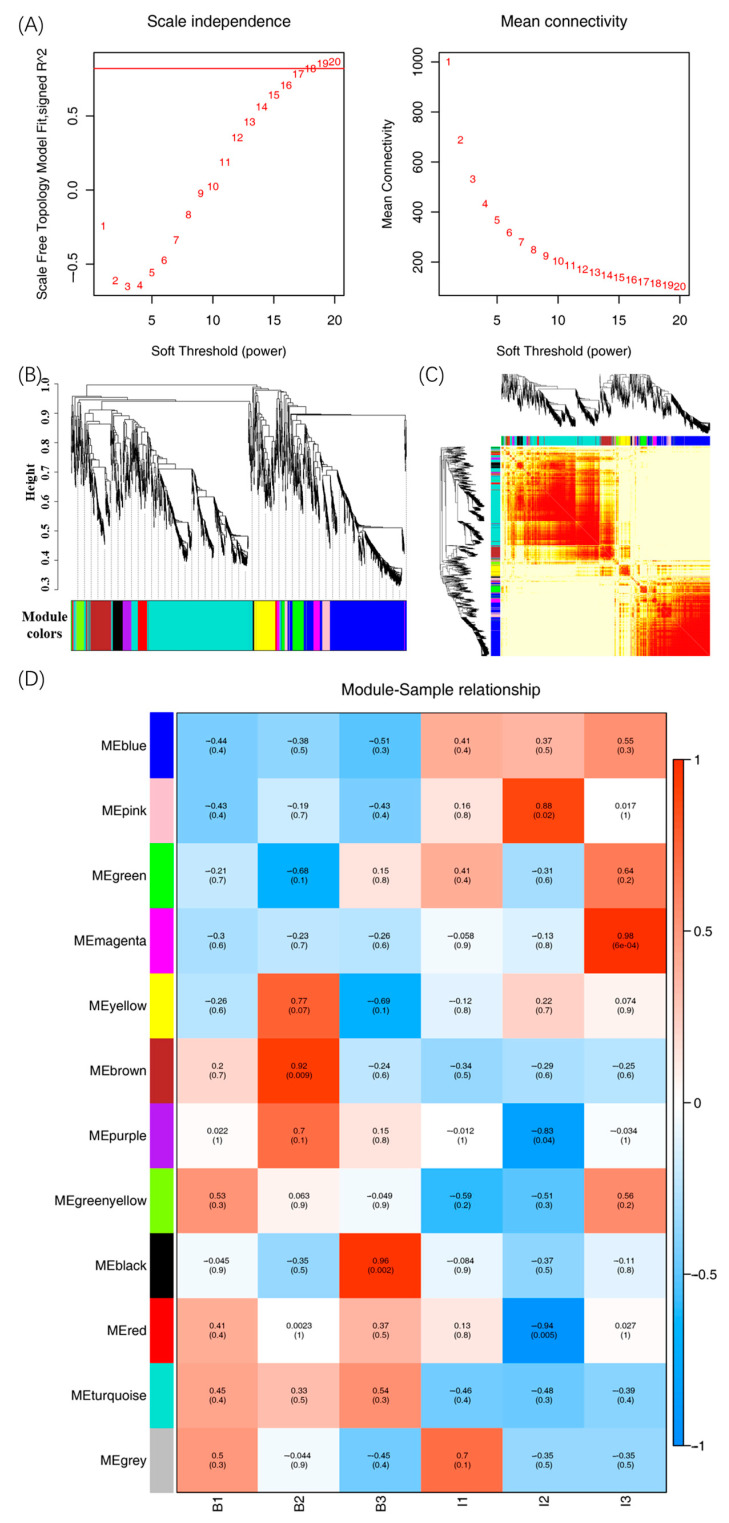
Co-expression network analysis identifying protein modules. (**A**) Analysis of soft-thresholding power in the WPCNA, (**B**) hierarchical cluster tree of differential proteins, (**C**) heat map of differential protein modules with a topological overlap matrix, and (**D**) module–sample relationship in differential proteins.

Weighted protein co-expression network analysis (WPCNA) has been conducted to explore the co-expression network of the differential proteins in food studies [40,41]. As shown in Figure 7A, the soft thresholding power was selected as 20, and the scale-free topology fitting index R2 values of the fits were set to 0.8. The cluster dendrogram in Figure 7B was obtained by clustering the correlation between protein expression levels and dividing it into modules. The heatmap plot of the topological overlap in the protein network was developed for further analysis of the correlation between the two proteins in these identified differential proteins (Figure 7C). A total of 12 modules were obtained through hierarchical clustering in Figure 7D. The color module was linked with the differential proteins for further analysis.

### 3.7. Analysis of the Protein–Protein Interaction Networks

**Figure 8:**
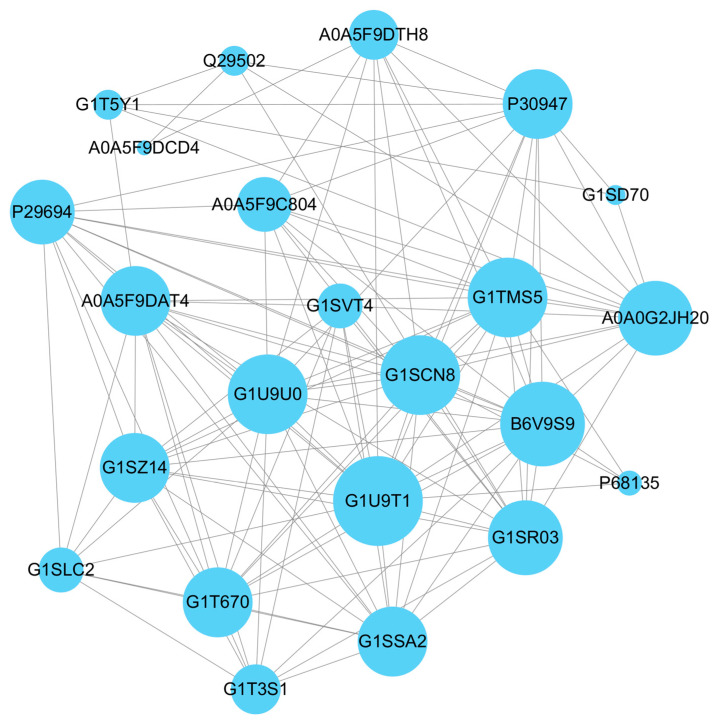
The protein–protein interaction network of the top 30 node degrees of differential proteins. Each node represents a differential protein. Each line represents the interaction between two differential proteins. The size of node indicates the interaction numbers among the specific differential protein with other differential proteins.

The analysis of protein–protein interaction networks was performed based on the top 30 node degrees of differential proteins. As shown in Figure 8, the protein–protein interaction networks had a total of 24 nodes and 123 edges with an enrichment *p*-value below 1.0 × 10^−16^. The 14 key proteins with high connectivity included G1U9T1, B6V9S9, G1SCN8, G1TMS5, G1U9U0, A0A0G2JH20, G1SR03, A0A5F9DAT4, G1SSA2, G1SZ14, G1T670, P30947, P29694, and A0A5F9C804, which better revealed the potential interactions between the target differential proteins (Table 1). The G1U9T1, B6V9S9, G1SCN8, G1TMS5, and G1U9U0 belonged to the TCP-1 chaperonin family, which could boost the correct conformation folding of the newly synthesized protein and polypeptides [42]. The heat shock protein HSP 90-alpha served as a molecular chaperone in the activation and maturation process of proteins [43]. The transitional endoplasmic reticulum ATPase was involved in protein biosynthesis processes such as the folding and assembly of proteins [44]. The 26S proteasome non-ATPase regulatory subunit played a key role in the maintenance of protein homeostasis [45]. Proteasome subunit alpha was an essential function of a modified proteasome, which could join in the mycotoxin stimulation and MHC Class I recognition reaction in rabbits [46]. The elongation factor 1-gamma and glucose-regulated protein participated in the response of antigenic stimulation through the biological regulatory system [47,48]. Thus, these key differential proteins could participate in the induction of immune responses and biological reactions after the external stimuli with the immunization of PAT antigen.

## 4. Conclusions

Considering the poor immune effect of the PAT mycotoxin for antibody production, this study aimed to deeply investigate the immune response of serum in rabbit immunization by 4D-DIA quantitative proteomics. The complete antigens for patulin were prepared and identified for rabbit immunization and serum evaluation. The serum proteomics revealed the functional annotations, functional enrichment analysis, co-expression network analysis, and protein–protein interaction network of differential proteins in serum, which could provide new experimental evidence for immunization with food hazards and be a useful tool for exploring the mechanism of protein interaction and antibody production in animal immunization. Moreover, the diversity of antibodies could also be analyzed to explore the bioinformatics characteristics of proteins in the immune serum, including antibodies and peptides. The germline antibody and its features could be further investigated by antibody engineering technology.

## Figures and Tables

**Table 1:** Quantitative information on the key differential proteins.

Accession Number	Protein Name	Gene	Protein Regulated	FC	*p*-Value
G1U9T1	T-complex protein 1 subunit eta	CCT7	down	0.508072	0.004703
B6V9S9	T-complex protein 1 subunit beta	CCT2	down	0.507246	0.02178
G1SCN8	T-complex protein 1 subunit gamma	CCT3	down	0.498206	0.009301
G1TMS5	T-complex protein 1 subunit epsilon	CCT5	down	0.488531	0.000455
G1U9U0	T-complex protein 1 subunit delta	CCT4	down	0.526914	0.004409
A0A0G2JH20	Heat shock protein HSP 90-alpha	HSP90AA1	down	0.358797	0.014942
G1SR03	Transitional endoplasmic reticulum ATPase	VCP	down	0.46324	8.21 × 10^−5^
A0A5F9DAT4	26S proteasome non-ATPase regulatory subunit 1	PSMD1	down	0.662827	0.013399
G1SSA2	26S proteasome non-ATPase regulatory subunit 2	PSMD2	down	0.635052	0.019449
G1SZ14	Proteasome subunit alpha type-3	PSMA3	up	2.304367	0.003462
G1T670	Proteasome subunit alpha type	PSMA5	up	2.100609	0.001789
P30947	Heat shock protein HSP 90-beta	HSP90AB1	down	0.437356	0.003641
P29694	Elongation factor 1-gamma	EEF1G	down	0.643377	0.010648
A0A5F9C804	glucose-regulated protein	HSPA5	up	1.861571	0.002569

## Data Availability

Data will be made available on request.

## Supplementary Materials

The following supporting information can be downloaded at https://www.mdpi.com/article/10.3390/bios14070322/s1. Table S1: The identified proteins in the immune serum of rabbits by 4D-DIA proteomics.

## References

[1] Bacha S.A.S., Li Y., Nie J., Xu G., Han L., Farooq S. (2023). Comprehensive review on patulin and *Alternaria* toxins in fruit and derived products. Front. Plant Sci..

[2] Iqbal S.Z., Waseem M., Razis A.F.A., Bhatti I.A., Khaneghah A.M., Mohammed O.A., Lakshminarayanan S.P., Iqbal M. (2024). Mycotoxin patulin contamination in various fruits and estimating its dietary impact on the consumers: From orchard to table. Heliyon.

[3] Puel O., Galtier P., Oswald I.P. (2010). Biosynthesis and toxicological effects of patulin. Toxins.

[4] Li B., Chen Y., Zong Y., Shang Y., Zhang Z., Xu X., Wang X., Long M., Tian S. (2019). Dissection of patulin biosynthesis, spatial control and regulation mechanism in *Penicillium expansum*. Environ. Microbiol..

[5] Guo Y., Zhou Z., Yuan Y., Yue T. (2013). Survey of patulin in apple juice concentrates in Shaanxi (China) and its dietary intake. Food Control.

[6] Ji X., Li R., Yang H., Qi P., Xiao Y., Qian M. (2017). Occurrence of patulin in various fruit products and dietary exposure assessment for consumers in China. Food Control.

[7] Baert K., De Meulenaer B., Kamala A., Kasase C., Devlieghere F. (2006). Occurrence of patulin in organic, conventional, and handcrafted apple juices marketed in Belgium. J. Food Prot..

[8] Cho M.S., Kim K., Seo E., Kassim N., Mtenga A.B., Shim W.B., Lee S.H., Chung D.H. (2010). Occurrence of patulin in various fruit juices from South Korea: An exposure assessment. Food Sci. Biotechnol..

[9] Fan L., Hu H. (2024). Involvement of multiple forms of cell death in patulin-induced toxicities. Toxicon.

[10] Qiu Y., Chen X., Chen Z., Zeng X., Yue T., Yuan Y. (2022). Effects of selenium nanoparticles on preventing patulin-induced liver, kidney and gastrointestinal damage. Foods.

[11] Adunphatcharaphon S., Elliott C.T., Sooksimuang T., Charlermroj R., Petchkongkaew A., Karoonuthaisiri N. (2022). The evolution of multiplex detection of mycotoxins using immunoassay platform technologies. J. Hazard. Mater..

[12] Hong S.P., Zakaria S.N.A., Ahmed M.U. (2022). Trends in the development of immunoassays for mycotoxins and food allergens using gold and carbon nanostructured material. Food Chem. Adv..

[13] McElroy L.J., Weiss C.M. (1993). The production of polyclonal antibodies against the mycotoxin derivative patulin hemiglutarate. Can. J. Microbiol..

[14] Sheu F., Lee O., Shyu Y.T. (1999). The synthesis of antigens and the production of antibodies against patulin derivatives. J. Food Drug Anal..

[15] de Champdore M., Bazzicalupo P., De Napoli L., Montesarchio D., Di Fabio G., Cocozza I., Rossi M., D’Auria S. (2007). A new competitive fluorescence assay for the detection of patulin toxin. Anal. Chem..

[16] Mhadhbi H., Benrejeb S., Martel A. (2005). Studies on the affinity chromatography purification of anti-patulin polyclonal antibodies by enzyme linked immunosorbent assay and electrophoresis. Food Addit. Contam..

[17] Song X., Wang D., Kim M. (2019). Immunoliposome-based fluorometric patulin assay by using immunomagnetic nanoparticles. Microchim. Acta.

[18] Fliege R., Metzler M. (2000). Electrophilic properties of patulin. N-acetylcysteine and glutathione adducts. Chem. Res. Toxicol..

[19] Glaser N., Stopper H. (2012). Patulin: Mechanism of genotoxicity. Food Chem. Toxicol..

[20] Duncan H., Mercader J.V., Agulló C., Gil-Sepulcre M., Abad-Somovilla A., Abad-Fuentes A. (2021). Chemical strategies for triggering the immune response to the mycotoxin patulin. Sci. Rep..

[21] Shuken S.R. (2023). An introduction to mass spectrometry-based proteomics. J. Proteome Res..

[22] Afzaal M., Saeed F., Hussain M., Shahid F., Siddeeg A., Al-Farga A. (2022). Proteomics as a promising biomarker in food authentication, quality and safety: A review. Food Sci. Nutr..

[23] Pedreschi R., Hertog M., Lilley K.S., Nicolai B. (2010). Proteomics for the food industry: Opportunities and challenges. Crit. Rev. Food Sci..

[24] Cho W.C.S. (2007). Proteomics technologies and challenges. Genom. Proteom. Bioinf..

[25] Hou X.H., Zhou P.Y., Gong P.Y., Fu J.L., Liu C., Wang H.P. (2022). Progress in data analysis methods for proteome mass spectrometry based on data-independent acquisition. Prog. Biochem. Biophys..

[26] VanDuijn M.M., Dekker L.J., van IJcken W.F., Sillevis Smitt P.A., Luider T.M. (2017). Immune repertoire after immunization as seen by next-generation sequencing and proteomics. Front. Immunol..

[27] Boutz D.R., Horton A.P., Wine Y., Lavinder J.J., Georgiou G., Marcotte E.M. (2014). Proteomic identification of monoclonal antibodies from serum. Anal. Chem..

[28] Sennels L., Salek M., Lomas L., Boschetti E., Righetti P.G., Rappsilber J. (2007). Proteomic analysis of human blood serum using peptide library beads. J. Proteome Res..

[29] Zhu J., Dou J., Wu C., Fan G., Li T., Shen D. (2023). Intestinal barrier protective study of jujube peel polyphenols/zein complexes by a combined Caco-2 cell and caenorhabditis elegans model: A perspective of proteomics. J. Agric. Food Chem..

[30] Sakamoto S., Nagamitsu R., Yusakul G., Miyamoto T., Tanaka H., Morimoto S. (2017). Ultrasensitive immunoassay for monocrotaline using monoclonal antibody produced by N,N’-carbonyldiimidazole mediated hapten-carrier protein conjugates. Talanta.

[31] Xu N., Zhu Q., Zhu J., Jia J., Wei X., Wang Y. (2021). Novel latex microsphere immunochromatographic assay for rapid detection of cadmium ion in asparagus. Foods.

[32] Tran B.Q., Hernandez C., Waridel P., Potts A., Barblan J., Lisacek F., Quadroni M. (2011). Addressing trypsin bias in large scale (phospho) proteome analysis by size exclusion chromatography and secondary digestion of large post-trypsin peptides. J. Proteome Res..

[33] Kodangattil S., Huard C., Ross C., Li J., Gao H., Mascioni A., Hodawadekar S., Naik S., Min-debartolo J., Visintin A. (2014). The functional repertoire of rabbit antibodies and antibody discovery via next-generation sequencing. MAbs.

[34] Weber J., Peng H., Rader C. (2017). From rabbit antibody repertoires to rabbit monoclonal antibodies. Exp. Mol. Med..

[35] Christopher J.A., Stadler C., Martin C.E., Morgenstern M., Pan Y., Betsinger C.N., Rattray D.G., Mahdessian D., Gingras A., Warscheid B. (2021). Subcellular proteomics. Nat. Rev. Methods Prime.

[36] Drissi R., Dubois M.L., Boisvert F.M. (2013). Proteomics methods for subcellular proteome analysis. FEBS J..

[37] Cui X., Xu X., Huang P., Bao G., Liu Y. (2022). Safety and efficacy of the *Bordetella bronchiseptica* vaccine combined with a vegetable oil adjuvant and multi-omics analysis of its potential role in the protective response of rabbits. Pharmaceutics.

[38] Li Y., Kong Y., Yu X., Yu W., Wen K., Shen J., Wang Z. (2023). Characteristics of rabbit hapten-specific and germline-based BCR repertoires following repeated immunization. One Health Adv..

[39] Wine Y., Boutz D.R., Lavinder J.J., Miklos A.E., Hughes R.A., Hoi K.H., Jung S.T., Horton A.P., Murrin E.M., Ellington A.D. (2013). Molecular deconvolution of the monoclonal antibodies that comprise the polyclonal serum response. Proc. Natl. Acad. Sci. USA.

[40] Liu F.J., Shen S.K., Chen Y.W., Dong X.P., Han J.R., Xie H.J., Ding Z.W. (2022). Quantitative proteomics reveals the relationship between protein changes and off-flavor in Russian sturgeon (*Acipenser gueldenstaedti*) fillets treated with low temperature vacuum heating. Food Chem..

[41] Shen S., Liu F., Chen Y., Xie H., Hu H., Ren S., Ding Z., Bu Q. (2022). Insight into the molecular mechanism of texture improvement of sturgeon fillets treated by low temperature vacuum heating technology using label-free quantitative proteomics. Food Res. Int..

[42] Brackley K.I., Grantham J. (2009). Activities of the chaperonin containing TCP-1 (CCT): Implications for cell cycle progression and cytoskeletal organisation. Cell Stress Chaperon..

[43] Garrido C., Gurbuxani S., Ravagnan L., Kroemer G. (2001). Heat shock proteins: Endogenous modulators of apoptotic cell death. Biochem. Biophys. Res. Commun..

[44] Luo Q., Qin X., Qiu Y., Hou L., Yang N. (2018). The change of synovial fluid proteome in rabbit surgery-induced model of knee osteoarthritis. Am. J. Transl. Res..

[45] Chondrogianni N., Sakellari M., Lefaki M., Papaevgeniou N., Gonos E.S. (2014). Proteasome activation delays aging in vitro and in vivo. Free Radic. Bio. Med..

[46] Seifert U., Bialy L.P., Ebstein F., Bech-Otschir D., Voigt A., Schröter F., Prozorovski T., Lange N., Steffen J., Rieger M. (2010). Immunoproteasomes preserve protein homeostasis upon interferon-induced oxidative stress. Cell.

[47] Sasikumar A.N., Perez W.B., Kinzy T.G. (2012). The many roles of the eukaryotic elongation factor 1 complex. WIREs RNA.

[48] Lee A.S. (2001). The glucose-regulated proteins: Stress induction and clinical applications. Trends Biochem. Sci..

