# Supplementary material for: Evaluation of the Immune Response of Patulin by Proteomics
Source: Biosensors (Basel). 2024 Jun 27;14(7):322. doi: 10.3390/bios14070322 (PMC11274796; doi:10.3390/bios14070322)
Supplement: Supplementary file 1 [file biosensors-14-00322-s001.zip › biosensors-3032780-supplementary.pdf]

Table S1. The identified proteins in immune serum of rabbits by 4D-DIA proteomics.

Accession	Peptides Count	B1	B2	B3	I1	I2	I3
A0A068BGJ4	2	1272.66	2234.03	1095.64	859.971	934.098	938.225
A0A0G2JH20	2	4941.5	3883.36	5846.3	1688.46	1660.77	1589.8
A0A140TAW0	2	626.536	1353.04	844.085	591.705	962.029	1066.74
A0A5F9C0Q4	9	5690.39	4575.72	6903.52	10241	9230.57	9670.19
A0A5F9C0R0	5	1711.28	1676.46	1419.79	1751.59	1348.77	2544.51
A0A5F9C0T2	3	51226.9	76975.7	46733.1	66126.8	91714.8	76972.5
A0A5F9C0T5	56	500936	561809	244514	264870	284636	273245
A0A5F9C0T9	1	3267.87	2129.08	1912.21	1382.33	582.298	
A0A5F9C0W8	5	1674.61	1244.96	1172.28	740.838	1663.58	861.447
A0A5F9C0X1	2	1649.55	1110.45	1630.49	492.034	1021.77	530.899
A0A5F9C0Y4	2	14244	15022.2	12718.6	7065.31	6995.5	3610.81
A0A5F9C100	10	4961.84	5373.19	4006.66	3437.87	3368.76	2852.31
A0A5F9C121	13	1890.76	1833.66	1446.05	2294.97	1517.15	1626.54
A0A5F9C125	18	7614.03	8426.3	5946.09	7183.45	7518.42	7010.34
A0A5F9C139	20	2307.17	2802.6	2095.65	4889.34	4930.81	4870.75
A0A5F9C145	41	7571.05	8107.95	7208.45	5649.01	5328.38	5509.52
A0A5F9C148	6	1763.86	2070.51	1610.38	2369.99	2889.05	3177.94
A0A5F9C152	12	20192.9	19638.3	12722.2	35225.4	33952.9	40124.3
A0A5F9C158	4	1740.76	1441.96	1169.79	941.074	1189.57	1071.1
A0A5F9C162	11	5965.34	5245.71	6239.6	2862	2859.78	2755.42
A0A5F9C164	3	14563.5	11014.2	10775.9	6572.77	6628.7	6003.71
A0A5F9C181	2	559.845	582.646	363.143	393.044	493.451	580.248
A0A5F9C185	8	13822.2	15347.9	11097.5	10306.8	10920.9	8100.51
A0A5F9C192	8	4009.36	4406.83	4322.97	3008.84	2150.66	2060.06
A0A5F9C1B3	1	8850.76	5124.82	7639.06	14086.1	13127.5	15410.4
A0A5F9C1B6	2	960.584	852.985	1263.48	735.211	1153.18	880.034
A0A5F9C1B7	1	1623.32	1981.28	312.833	1123.17	940.554	1809.69
A0A5F9C1C3	4	2047.95	1788.55	1430.27	2115.78	2610.53	2164.82
A0A5F9C1C5	13	119967	316383	99183.6	448373	672628	463331
A0A5F9C1E0	8	2840.35	2697.33	2764.97	2212.78	1788.14	2284.84
A0A5F9C1F3	4	462.216	639.067	902.143	925.636	1039.34	1096.21
A0A5F9C1H6	4	630.158	633.419	863.16	686.462	780.548	186.266
A0A5F9C1I1	3	1241.09	985.702	1470.09	803.87	805.763	871.12
A0A5F9C1I2	16	1666.12	1711.53	1241.31	1446.38	1242.57	1235.02
A0A5F9C1I6	9	2063.56	1816.38	2112.4	1271.67	1288.89	1201.54
A0A5F9C1I7	5	2291.49	2396.31	2483.85	1904.54	1955.47	1813.06
A0A5F9C1K7	3	1201.88	1123.24	949.589	572.598	786.163	567.858
A0A5F9C1T6	10	3094.97	2671.25	3955.93	3610.5	3414.92	4429.87
A0A5F9C1W1	5	1202.09	1147.23	1162.98	2113.2	2170.7	1913.59
A0A5F9C1W5	51	8635.57	13074.5	9465.18	7253.9	6911.54	6647.43
A0A5F9C207	1	470.345	838.984	1260.55	633.961	735.716	576.83
A0A5F9C214	8	1692.52	1411.08	1484.56	745.464	835.974	825.174
A0A5F9C255	3	1467.27	1939.59	1696.4		1939.06	
A0A5F9C260	2	2122.24	1494.32	1622.48	1409.44	1089.31	1266.78
A0A5F9C295	3	59746.5	41333.3	46705.7	105456	97732.3	94715.8
A0A5F9C2B4	29	8088.21	8148.16	8020.81	5175.91	5851.86	5527.44
A0A5F9C2C6	2	1198.3	1926.69	2060.28	1240.12	1019.36	987.321

A0A5F9C2D3	3	1810.85	1003.87	1556.3	2142.4	1395.82	1660.93
A0A5F9C2E0	3	3688.92	2676.93	1766.98	2595.51	2926	3008.35
A0A5F9C2E1	1	2027.77	658.016	1047.57	517.663	534.286	384.718
A0A5F9C2G0	2	1499.58	1107.78	1372.9			
A0A5F9C2K9	2	1167.37	1395.13	1267.02	1622.76	2023.42	2028.47
A0A5F9C2M9	2	524.339	526.283	452.326			
A0A5F9C2N4	7	4686.76	5813.5	1401.11	1352.84	547.951	597.572
A0A5F9C2N6	1	1948.38	1549.53	1716.29	1026.61	1334.52	1569.86
A0A5F9C2Q1	1		476.769	421.004	308.833		673.323
A0A5F9C2R1	1			636.217			
A0A5F9C2T3	1	428.563	952.854	441.224	415.701	201.926	1051.08
A0A5F9C2W9	14	41187.5	56188	52762.7	92166.1	78635.6	100369
A0A5F9C2X4	10	1110.67	1195.03	545.285	1851.38	2039.84	2107.15
A0A5F9C2Y8	6	1079.44	1149.38	1073.29	847.289	701.424	692.24
A0A5F9C302	1	3438.94	4141.35	4953.8	1445.71	2182.65	2382.43
A0A5F9C306	6	2838.21	2647.42	2924.86	1468.48	1675.65	1554.33
A0A5F9C315	6	3164.42	3104.76	2954.27	2709	2507.41	2896.35
A0A5F9C318	1	28189.1	20057.2	27981.1	1175.89	496.908	882.906
A0A5F9C326	2	1215.99	1454.01	865.956	904.386	1024.1	461.839
A0A5F9C342	1	1305.21	3290.03	1266.53		357.211	355.07
A0A5F9C353	15	3408.87	2893.4	2757.24	4166.49	4045.42	4314.42
A0A5F9C369	1	15237.3	8305.57	5022	16811.9	16828.6	21927.2
A0A5F9C380	1						1334.47
A0A5F9C396	3	1767.89	1983.18	2050.72	2334.31	2319.81	2143.96
A0A5F9C3A1	33	83546.5	68893.6	79134.3	46986.2	40895.4	38647.5
A0A5F9C3A6	1			730.459			2524.83
A0A5F9C3B7	69	47242	80183.7	28644.7	34085.6	46129.7	34860.9
A0A5F9C3C4	18	14118.8	14571.6	10541.5	15586	15858.2	15758.5
A0A5F9C3D2	1	16917.1			1826.99		
A0A5F9C3E4	5	1050.22	771.719	1694.12	712.695		736.054
A0A5F9C3E7	6	1333.41	1336.89	1797.17	1517.48	844.801	1393.56
A0A5F9C3G7	1	1306.81	573.733	1003.56	807.497	401.859	577.335
A0A5F9C3N0	1	17835.2	12476.3	10382.3	13592.8	14895.3	16058
A0A5F9C3R0	1	1505.18	1893.57	499.289	827.765	738.155	1121.35
A0A5F9C3R7	12	3263.95	4039.13	2568.22	2258.57	2360	2754.03
A0A5F9C3R8	6	28437.1	38698.5	22375.7	24693.8	30298.1	29185.7
A0A5F9C3S1	11	3325.74	3176.98	3304.71	2133.65	1922.07	1997.78
A0A5F9C3S2	2	374.707		661.918			
A0A5F9C3S3	1	784.71	1358.19	965.858	822.083		566.635
A0A5F9C3S6	11	10257.3	10045.6	11696.5	18838.2	20401.4	16648.2
A0A5F9C3T1	3	1164.47	847.672	1311.98	1125.4	867.275	1112.97
A0A5F9C3V2	10	10328.4	10675	13524.7	14705.4	11618	14545.4
A0A5F9C3W2	8	1940.36	2040.66	2413.43	1237.86	1017.86	894.96
A0A5F9C402	22	14433.4	18312.1	13530.3	18109.4	19115.6	20414.5
A0A5F9C409	2	26421.3	26882.1	27868.8	23285.4	24984.6	23272.8
A0A5F9C428	12	1926.21	2344.24	2442.49	1278.22	1330.5	1805.79
A0A5F9C432	6	1785.09	2201.24	1920.94	1861.95	1999.89	1944.21
A0A5F9C434	6	3165.17	2891.86	3586.79	1999.37	2262.13	2263.19
A0A5F9C454	24	34702.2	30730.9	41195.1	16605.2	11712.2	14673.8

A0A5F9C456	2	676.341	450.769	577.808	640.957	820.482	504.179
A0A5F9C462	3	1472.69	1960.11	1395.1	1474.5	920.121	1450.88
A0A5F9C469	5	1593.17	1413.68	1416.36	994.634	867.901	766.968
A0A5F9C476	3	1303.92	724	1156.25	1158.7	590.444	841.157
A0A5F9C492	2	1252.53	1365.48	1073.94			
A0A5F9C494	3	659.064	312.605	659.279	263.12		250.781
A0A5F9C497	9	2742.28	2315.68	2650.14	2042.7	2321.19	2197.08
A0A5F9C4A2	5	1244.66	878.616	968.276	875.827	1001.03	966.577
A0A5F9C4A7	23	4399.15	4132.03	4063.77	2560.95	2813.91	2548.64
A0A5F9C4B0	10	13150.3	13348.7	11160.6	11373	10266.4	12027.5
A0A5F9C4B1	5	479.295	708.412	627.868	1592.82	1581.32	1279.71
A0A5F9C4B7	20	63049.8	82633.4	40170.8	64874.9	67423.8	56039
A0A5F9C4D4	9	1928.23	2271.25	1467.55			
A0A5F9C4D8	4	7689.28	9020.38	7863.27	3877.26	5216.87	5964.08
A0A5F9C4F5	4	1705.06	2240.4	879.695	1482.33	1089.08	927.215
A0A5F9C4G7	36	1565.63	1455.97	1654.44	4935.5	4108.44	4397.55
A0A5F9C4I3	6	3756.06	4337.28	3293.94	6285.84	5126.34	5174.57
A0A5F9C4J3	43	7143.66	7986.57	5688.75	11617.1	10220.4	12007.8
A0A5F9C4N0	9	3022.1	2658.27	3810.08	3083.9	2451.07	3289.75
A0A5F9C4N8	1	1267.11	1118.88	1625.68	3748.98	2812.28	2913.66
A0A5F9C4R6	5	5449.55	7955.17	978.18	1505.55	1063.73	1442.26
A0A5F9C4U8	8	6070.3	5906.85	6115.76	4457.61	4595.95	4378.62
A0A5F9C4V2	5	3044.55	4424.71	3747.09	4523.39	4765.04	5415.21
A0A5F9C4W7	9	6430.44	13575.4	5956.31	14805.3	16767	14895.8
A0A5F9C4Y1	3	1947.42	2336.51	2943.39	1195.99	1117.49	1009.1
A0A5F9C4Y3	2	2083.17	1403.43	2007.14	1126.69	777.641	1028.3
A0A5F9C4Z2	18	3888.99	3853.82	4232.5	2615.89	2583.45	2404.3
A0A5F9C548	7	1817.81	2001.35	1884.26	1992.59	1943.02	2029.64
A0A5F9C564	1	7305.05	25155.2	3461.01	25654.6		25193.1
A0A5F9C5D0	3	3120.14	4576.35	2611.01	3235.36	3352.55	3255.87
A0A5F9C5D3	4	2277.66	2535.47	2354.89	1490.41	1079.93	1571.72
A0A5F9C5E0	2	1518.66	683.578	1736.09	919.417		559.707
A0A5F9C5H9	5	2535.42	2643.08	2334.29	1978.56	1371.71	1791.59
A0A5F9C5J0	1	35365.3	35820.5	30091.2	62501.5	90181.6	
A0A5F9C5N8	2	543.412	945.55	417.381	868.842	1973.88	1197.96
A0A5F9C5Q7	6	4029.65	6304.01	6093.99	5673.9	5743.9	6239.06
A0A5F9C5R6	2	3807.85	5287.65	3554.69	4012.35	4337.29	3404.26
A0A5F9C5R8	1					1276.64	
A0A5F9C5S9	2		62.2471	477.057	180.737		
A0A5F9C5T7	4	1138.49	1279.78	838.673	873.502	1226.25	869.762
A0A5F9C5U2	2	712.517	737.596	800.539	611.353	583.84	711.1
A0A5F9C5W9	2		366.787	383.972	665.19		378.107
A0A5F9C5Y2	1	586.723		778.9		720.108	606.081
A0A5F9C5Y4	12	3659.28	3370.8	4078.69	3186.89	2458.36	2569.71
A0A5F9C637	3	1426.1	1755.75	1108.68	2758.35	1547.52	2022.99
A0A5F9C648	3	2140.92	3216.62	1658.58	6061.72	5098.55	5639.33
A0A5F9C656	6	2298.97	2401.32	1435.51	1493.57	1375.62	1305.24
A0A5F9C671	1	1753.25	2061.11		2828.77	3662.51	
A0A5F9C6A2	4	1197.28	748.637	1415.16	1553.71	984.306	1415.53

A0A5F9C6A6	1	44263.8	42182	49866.6		16946.4	16826.6
A0A5F9C6A7	3	1029.07	1799.32	946.706	1549.92	1056.96	946.909
A0A5F9C6B1	24	3041.05	2777.33	2762.53	3916.01	3818.35	4025.28
A0A5F9C6C9	32	99749.9	87347.8	100306	154916	146638	165658
A0A5F9C6E6	14	13081	14870.7	10364.5	12824.6	11285.5	12249.8
A0A5F9C6G1	7	1406.9	1393.77	1464.38	2518.74	2138.62	2034.31
A0A5F9C6K5	1	1819.33	1044.98	1276.34	515.635		481.891
A0A5F9C6K9	14	5820.18	4689.8	5124.7	2891.61	3137.47	2647.08
A0A5F9C6M2	1	1287.12	1017.44	1671.37	1508.06	1181.12	1523.74
A0A5F9C6N1	1	638.95	694.242	582.807	542.799	299.684	484.619
A0A5F9C6P1	27	11104.7	13163.7	8239.04	13628.1	12490.2	13768.8
A0A5F9C6Q8	1					14509.2	
A0A5F9C6T8	1	1260.45	624.656		595.427		
A0A5F9C6U0	8	888.422	1266.02	1027.19	639.425	670.773	574.408
A0A5F9C6V3	15	4026.62	4461.92	3965.64	4840.26	3997.68	4561.17
A0A5F9C6Y9	1		621.518			591.302	946.401
A0A5F9C713	8	2037.47	2333.67	1882.2	1481.87	1378.74	1515.31
A0A5F9C720	32	79800.6	82859.2	82809	141787	145353	135937
A0A5F9C722	5	995.953	1057.73	713.505	633.001	749.367	1041.78
A0A5F9C736	3	1481.9	1810.58	1232.11	1119.43	644.989	891.279
A0A5F9C740	5	4205.16	4243.18	4432.32	4699.64	5070.07	4413.15
A0A5F9C791	23	79041.9	68926.2	80443.6	35928.3	34986.9	37501
A0A5F9C797	6	3334.75	3232.59	2828.66	2699.36	2322.8	2304.6
A0A5F9C7A0	2	1748.31	1004.77	885.457	732.559	710.372	1318.28
A0A5F9C7D2	6	1637.59	1282.96	2067.99	1347.46	852.619	1208.54
A0A5F9C7D9	14	2281.66	2557.61	2895.08	1854.7	1395.52	1675.38
A0A5F9C7G2	2	793.796	1048.26	824.765	1767.35	1052.92	1360.06
A0A5F9C7N2	2	1363.9	666.033	1296.72	845.138	811.232	797.558
A0A5F9C7N8	1		1479.84	531.098	731.474	488.925	912.432
A0A5F9C7N9	12	3032.72	3106.58	3113.42	4411.77	3570.65	4458.79
A0A5F9C7Q2	8	3378.99	5771.24	2927.72	3370.42	4179.87	3289.81
A0A5F9C7S7	4	1310.2	1941.83	926.672	1082.81	1048.53	931.004
A0A5F9C7U7	16	4308.6	4668.41	4568.63	2591.86	2532.78	2251.12
A0A5F9C7V0	2	1318.99	939.771	1535.73	1093.12	822.769	534.261
A0A5F9C7V5	3	2170.67	1691.93	1747.83	1014.5	844.207	1044.65
A0A5F9C804	32	14787.8	15387.1	12572.2	25370.6	23973.9	25076
A0A5F9C813	2	1411.86	1459.2	1313.33	2556.37	1958.87	2455.46
A0A5F9C843	3	2102.85	1690.24	1731.94	1665.34	1016.63	1403.82
A0A5F9C848	17	25255.6	25435.7	20928.8	49414.4	42433.8	48812.5
A0A5F9C865	5	1977.67	1887.03	2368.4	1188.09	1014.75	982.264
A0A5F9C8A2	2	3574.04	5010.02	3353.55	3827.5	3774.59	3522.38
A0A5F9C8C3	2	1323.9	1044.65	1437.66	1603.17	1380.44	1514.6
A0A5F9C8D3	4	39749.5	43709.3	46658.5	17528	18744.2	28050.3
A0A5F9C8E0	1	2935.86	3228.46	3535.4	504.922	777.954	872.052
A0A5F9C8F6	35	6213.26	4146.68	3705.55	13267.9	14951.5	12401.9
A0A5F9C8G2	13	3222.66	3505.35	3791.24	3958.95	3004.73	3309.29
A0A5F9C8I7	2	861.305	850.802	767.337	354.786	379.739	565.457
A0A5F9C8I8	10	4919.02	4642.47	4119.56	2082.65	1907.83	2128.67
A0A5F9C8I9	3	8621.19	10819	9472.94	11833	10738.3	14104.7

A0A5F9C8J1	4	423.882	1025.65	788.758	459.641	693.115	985.126
A0A5F9C8K6	10	2238.13	1796.19	2640.43	1207.63	574.833	913.102
A0A5F9C8M6	2	730.232	993.809	783.08	951.098	816.742	983.696
A0A5F9C8P7	1	580.885	344.605	774.333		717.523	390.995
A0A5F9C8Q8	4	742.634	1289.35	648.036	1658.8	1615.37	1530.65
A0A5F9C8S6	1	1636.52	1521.31	1665.58	784.897	1289.28	1828.08
A0A5F9C8W6	3	929.368	359.689	504.45	719.585	708.786	637.812
A0A5F9C8X3	1		318.801	246.257	451.869		428.161
A0A5F9C906	12	5508.87	6967.91	4713.23	9391.07	7793.37	9043.06
A0A5F9C975	2	18515.1	38886.1	18736.9	25260.9	29306.9	30925.4
A0A5F9C983	13	3561.94	3888.73	4219.32	3441.41	3806.38	3786.91
A0A5F9C9G6	1	332.369	777.913			615.788	
A0A5F9C9J7	1	324.504	745.554		488.535	939.898	964.432
A0A5F9C9K1	2	563.842	576.425	541.607			396.843
A0A5F9C9L9	4	1992.5	2361.26	1057.48	3410.03	3107.9	2467.73
A0A5F9C9Q0	8	5755.85	4792.99	4138.16			
A0A5F9C9Q2	2	1013.17	1194.44	1026.8	2463.57	1779.82	1820.52
A0A5F9C9S1	11	10451.7	11681.8	8756.18	7777	8790.74	8076.64
A0A5F9C9S5	1	1211.63	1651.57	1667.76	1328.59	713.326	582.925
A0A5F9C9X1	17	2343.05	2790.56	3015.5	1817.95	1641.42	2156.66
A0A5F9C9Y3	1		971.849		2370.08	2842.44	1820.96
A0A5F9C9Y7	19	2650.74	2604.61	2875.04	2041.58	2176.01	1670.9
A0A5F9CA23	1	728.588	835.232	316.972	1430.68	665.416	1570.52
A0A5F9CA51	8	1701.86	1383.9	1564.65	1015.62	422.009	786.761
A0A5F9CA78	4	3658.68	5073.36	2705.05	5951.23	5961.81	5861.33
A0A5F9CAB6	1	1119.54	1675.62	736.412	879.705		283.966
A0A5F9CAD3	4	1111.59	892.531	1217.36	743.192	520.168	666.466
A0A5F9CAI9	10	2310.85	1889.94	2968.86	1428.31	923.258	1318.15
A0A5F9CAK1	3	1300.95	1566.03	1387.42	1310.37	1201.14	1147.03
A0A5F9CAP0	21	18898.6	21790.9	13028.1	12145.2	10944.4	15088
A0A5F9CAU8	11	1783.06	2082.36	912.796			
A0A5F9CAX1	10	19194	14613.9	18502	10394.4	9629.27	11818.5
A0A5F9CAY6	2	320.739	609.721	375.296	699.101	627.34	1014.86
A0A5F9CAY7	12	2894.6	2676.14	3914.95	1980.49	1548.01	1736.38
A0A5F9CB17	18	733350	576953	771521	1.23E+006	1.08E+006	1.21E+006
A0A5F9CB42	3	1372.57	1308.11	1424.19	582.053	565.806	656.221
A0A5F9CB44	2	1497.66	1616.05	1410.83	1701.41	854.582	1513.81
A0A5F9CB63	1	7777.44	14099.8	6202.13			410.451
A0A5F9CB66	3	1692.6	1797.3	1192.98	1048.56	677.072	641.237
A0A5F9CBB3	1	415.219	1444.49		645.377	1293.62	1350.68
A0A5F9CBB8	2	688.827	422.148	833.825	678.447		623.878
A0A5F9CBG4	2	457.522	342.609	197.952	289.239	260.946	94.4122
A0A5F9CBG5	3	999.396	1038.22	1370.62	1755.51	1769.56	1835.76
A0A5F9CBG7	6	2997.32	1855.15	2239.74	2735.39	2270.73	2571.86
A0A5F9CBH5	2		1306.94		1047.95	1067.76	884.453
A0A5F9CBJ5	4	3044.68	2092.94	3814.41	1745.85	1898.98	1913.21
A0A5F9CBM7	4	2988.08	2717.36	2728.49	1601.16	1660.27	1340.57
A0A5F9CBN6	6	1247.13	898.682	1330.49	1139.6	1212.82	1256.88
A0A5F9CBP8	2				1704.71	1492	1877.27

A0A5F9CBS3	1	5835.99	6956.24	3213.22	11473.7	11217.2	10366.4
A0A5F9CBZ0	11	1127.12	1060.01	1093.71	697.7	674.278	864.488
A0A5F9CC03	2	845.984	1158.66	1059.94	1170.39		1453.48
A0A5F9CC05	14	3361.53	3380.61	3247.45	1945.56	1718.72	1800.05
A0A5F9CC17	1	482.063	859.079	502.853	943.294	1218.7	1010.5
A0A5F9CC27	5	1361.92	1128.7	1236	830.552	950.69	941.479
A0A5F9CC29	2	5542.39	3640.57	11958.3	4643.75	11404.8	7310.46
A0A5F9CC54	8	2860.31	2804.46	2467.58	6979.39	5631.57	6314.44
A0A5F9CC60	3	7103.2	6726.48	6319.99	5268.87	5831.53	4598.38
A0A5F9CC70	2	1339.27	938.522	1427.44	1252.74	2479.66	1162.65
A0A5F9CCC4	1	1073.83	966.457	1891.32	1191.3	555.349	367.915
A0A5F9CCH4	1	1227.39		1257.79			
A0A5F9CCH5	9	1775.73	1557.65	2107.71	1433.24	1476.68	1444.63
A0A5F9CCH8	3	1603.63	1180.13	1136.01	893.875	1127.08	800.021
A0A5F9CCJ1	7	367382	169507	476668	189542	202061	263525
A0A5F9CCJ3	13	5682.66	5185.63	5970.91	5412.23	4810.63	5857.43
A0A5F9CCJ6	32	885916	391705	774285	165292	168903	170163
A0A5F9CCK8	1	1207.5	846.605	927.683	1200.9	1301.62	1075.72
A0A5F9CCM9	3	2020.47	2463.93	2105.34	2550.93	2330.76	2668.76
A0A5F9CCU3	11	2132.88	2255.53	2186.86	4123.8	3743.23	3881.25
A0A5F9CCV3	1	52294.3	59422.5	51205.8	49581.9	34130.2	47608.1
A0A5F9CD26	1	7354.68	7072.51	13780	3236.72	2437.22	2510.86
A0A5F9CD31	2	1196.65	517.275	1363.07	1213.27	838.33	1930.85
A0A5F9CDC5	3	973.962	1395.55	761.256	783.389	634.264	873.538
A0A5F9CDC8	3	954.054	1337.88	938.395	946.237	567.908	752.664
A0A5F9CDD1	11	11441.7	7994.47	10658.9	15336.2	10869.9	14596.4
A0A5F9CDD8	5	2012.84	1718.31	2265.5	1862.14	1697.71	1685.18
A0A5F9CDE9	5	1206.74	1482.49	1075.6	1064.48	710.469	798.214
A0A5F9CDI0	2	368.008	351.411	449.59			157.971
A0A5F9CDK5	16	1351.9	1370.92	1511.47	1037.17	878.71	883.647
A0A5F9CDL7	1	417.49	431.434				199.717
A0A5F9CDP6	2	403.527	190.021	186.58			
A0A5F9CDT8	1	847.121	547.079	790.273			745.571
A0A5F9CDU4	4	904.371	888.085	1197.88	419.268	670.229	542.957
A0A5F9CDW8	7	1363.76	1201.07	1173.02	2008.67	1603.2	1704.58
A0A5F9CDY2	3	1688.04	2124.41	1159.27	1574.95	1115.12	1109.28
A0A5F9CE05	8	1.60E+006	1.07E+006	1.77E+006	1.36E+006	1.06E+006	1.39E+006
A0A5F9CE44	8	5521.22	6023.05	4716.57	4465.44	3574.06	3789.44
A0A5F9CE66	7	2049.51	1600.41	1545.32	952.445	692.637	785.416
A0A5F9CE77	1	855.077	518.92	651.987			366.554
A0A5F9CEA3	6	1627.45	2414.16	2495.18	1515.08	899.434	1546.68
A0A5F9CEA5	22	30714.2	33584.4	25762.5	90977.4	90499.5	92601.5
A0A5F9CEF0	1	1450.23	1368.54	2443.64		865.304	
A0A5F9CEL1	3	1946.38	1923.27	2137.83	1497.3	943.309	1330.6
A0A5F9CEM1	2	1299.63	1059.52	1055.4	760.501	930.256	655.099
A0A5F9CEN0	2	1378.34	1784.47	1932.46	723.368	656.524	1084
A0A5F9CER2	2	609.974	661.737	921.266	690.731	542.304	472.733
A0A5F9CEU5	1	1635.84	1704.42	1772.53	1604.99	1097.01	674.901
A0A5F9CEX0	1	1.22E+006	1.28E+006	729751	1.93E+006	3.03E+006	1.54E+006

A0A5F9CEX6	6	852.687	1267.67	1157.42	2099.51	1887.28	2599.22
A0A5F9CEX8	2	662.818	648.913	987.324	856.715	453.969	652.647
A0A5F9CEZ2	3	623.25	1185.37	838.683	1098.93	884.186	779.43
A0A5F9CF18	5	1234.6	1038.13	1452.02	998.134	820.249	763.584
A0A5F9CF19	7	2153.32	3185.46	1462.59	1179.28	1443.79	1467.05
A0A5F9CF25	2	1790.05	1595.81	2151.28			
A0A5F9CF34	4	804.235	741.154	786.311	1348.15	1419	1407.05
A0A5F9CF38	2	2096.36	2259.53	2287.06	2507.39	2666.53	3101.99
A0A5F9CF43	7	2750.85	2742.14	1701.65	2622.12	2193.08	2686.65
A0A5F9CF44	2		404.362	502.685	1625.12	924.223	4604.28
A0A5F9CF51	1	1906.12	1442.03	4288.87		432.981	
A0A5F9CF58	3	891.956	803.748	203.01	984.622	1195.06	1199.08
A0A5F9CFB3	2	24862.8	8332.58	21368.1	34217.5	28662.8	45201.6
A0A5F9CFC1	1		280.204	332.951		550.597	421.731
A0A5F9CFE1	1	889.06	447.927	423.922		429.948	312.42
A0A5F9CFG5	1	507.964		360.534	688.705		
A0A5F9CFL5	1	779.028	732.238	769.416	433.468	371.035	500.357
A0A5F9CFM4	1	1175.2	743.963	1629.15		912.067	958.601
A0A5F9CFM5	1	12781.9	14558.4	9913.35	26103.5	24265.3	19975
A0A5F9CFN4	9	4257.13	4245.66	4647.33	3413.37	3841.12	4082.39
A0A5F9CFN8	3	1598.25	2220.68	2350.51	2338.71	5081.89	2621.28
A0A5F9CFP1	19	56455.6	92024.5	40980.1	87590.6	95846.2	92354.6
A0A5F9CFP8	6	3004.42	3633.19	2891.56	1798.18	2086.69	2135.75
A0A5F9CFU1	29	3001.04	3374.39	3150.9	6530.18	6043.38	6158.16
A0A5F9CG11	1	1502.64	1132.92	766.855	2883.74	3131.33	2563.75
A0A5F9CG17	2	13037.7	26074.2	6990.54	6106.39	8536.45	6467.36
A0A5F9CG27	10	3200.19	2624.66	3641.96	3319.58	2631.37	3130.32
A0A5F9CG40	1	1340.56	951.403	957.851	889.274		493.13
A0A5F9CG43	2	611.292	717.75	754.833	611.309	607.703	678.653
A0A5F9CG53	3	2000.63	1942.41	970.114	2521.84	964.609	1325.2
A0A5F9CGE5	2	770.165	996.865	1283.23	1891.67	1078.34	2206.22
A0A5F9CGH3	3	1011.87	1066.51	1316.17		1228.23	1117.65
A0A5F9CGH5	4	645.298	595.281	870.847	804.352	650.059	862.349
A0A5F9CGI6	1	1899.1	1246.23	1911.27	1720.03	1434.77	1720.25
A0A5F9CGI8	1	1346.13	1068.48	1835.35			812.605
A0A5F9CGJ1	22	5510.08	6522.15	6252.66	11733.5	12506.9	12186.8
A0A5F9CGJ3	4	2136.22	1387.53	2383.89	1466.55	1043.52	1077.65
A0A5F9CGM9	5	1414.87	1760.77	915.55	1502.99	1294.94	1273.97
A0A5F9CGN3	1	10729.1	12117.2	9117.9	22955.2	17641.7	25494.8
A0A5F9CGP0	9	3629.56	3880.51	3252.18	7389.93	7428.11	6228.19
A0A5F9CGR4	32	9297.66	9234.73	9198.29	15868.4	15452.1	15905.8
A0A5F9CGS1	6	1549.78	1265.92	1547.69	1493.91	1069.34	964.514
A0A5F9CGU8	2	3216.84	3196.29	5859.36	4606.57	6074.97	4629.5
A0A5F9CGV1	2	1076.41	1132.05	692.974			715.92
A0A5F9CGW2	7	1165.82	1308.65	1273.49	1161.94	939.508	1071.85
A0A5F9CGY7	2	627.4	322.193	821.972	563.711		278.371
A0A5F9CH05	5	2131.2	1537.01	2022.67	1627.84	1416.22	1194.46
A0A5F9CH43	3	336.705	672.709	743.138	671.185	926.096	761.29
A0A5F9CH57	3	6948.76	6717.92	4395.93	11092.4	12538.1	13406

A0A5F9CH88	9	2024.51	2025.29	2492.2	1159.26	1085.51	1219.05
A0A5F9CHB9	3	1344.81	1169.43	820.401	1762.8	1724.71	2112.26
A0A5F9CHF0	41	428953	490000	303650	493674	502883	476484
A0A5F9CHL8	3	5290.24	6859.58	3576.91	538.631	1099.9	477.78
A0A5F9CHM5	2	2877.68	2304.77	2396.12	1559.44	1077.23	1216.32
A0A5F9CHP7	19	2858.07	2760.35	3148.38	2134.34	1905.84	2058.41
A0A5F9CHW0	8	1870.78	2360.23	2025.11	1820.98	1759.7	1827.74
A0A5F9CHW6	7	4545.72	4580.67	4118.42	3900.71	3449.03	3288.65
A0A5F9CI66	72	471896	504062	469221	433764	414577	450715
A0A5F9CIB4	8	4436.14	4735.45	3807.5	6268.25	5449.31	6023.35
A0A5F9CIC7	6	2315.74	2251.68	2221.61	4265.09	4100.01	4715.79
A0A5F9CIK2	8	1832.53	1459.74	1228.18	2662.91	2119.63	2507.28
A0A5F9CIN0	4	1979.07	2239.07	1859.13	1472.04	998.972	1192.61
A0A5F9CIR3	10	17148.2	14593.6	16019	18166.7	15562.3	19681.7
A0A5F9CIS6	1	484.29	1395.41	994.812	914.058	528.312	910.427
A0A5F9CIV7	3	728.198	797.177	1031.61	626.043	791.019	814.951
A0A5F9CIZ4	1	1501.08	994.966	842.929	1333.93	1210.64	1413.54
A0A5F9CIZ9	47	18600.9	30702.5	8859.68	20071.2	21962.9	21764.2
A0A5F9CJ00	11	2149.82	2150.36	2516.76	1609.42	1591.37	1618.42
A0A5F9CJ09	4	2073.62	2238.47	3239.34	2711.9	1949.72	2330.84
A0A5F9CJ13	4	1087.11	1095.92	711.079	2236.09	2397.12	2577.58
A0A5F9CJA1	9	165052	80547.5	213991	63163.4	58529.6	63360
A0A5F9CJC1	5	1749.66	2188.35	2386.31	1519.36	1335.42	1546.38
A0A5F9CJC8	2	2681.29	3645.35	1123.37	1580.35	1595.72	2054.31
A0A5F9CJE1	35	23519.9	19265.6	20862.6	39391.4	35331.5	36616.4
A0A5F9CJJ5	1	124153	111920	121995	49316.8	49701.4	55311.4
A0A5F9CJM9	2	1200.71	2007.41	482.408	1056.83	1351.99	1097.15
A0A5F9CJN5	3	562.34	579.617	574.967	1245.43	1239.98	1948.2
A0A5F9CJP4	3	2519.55	3192.03	1881.31	6029.05	7531.3	7327.98
A0A5F9CJQ2	63	215784	222933	182437	372369	346105	391403
A0A5F9CJT8	2	1940.84	2219.67	715.755	937.808	812.89	749.568
A0A5F9CJZ7	13	10714.9	13518.5	15366.2	9014.5	10981.8	7020.28
A0A5F9CK42	2	687.438	490.045	643.837	46.8551	233.533	
A0A5F9CKA1	2	270.321	482.535	207.341	506.348	671.127	447.963
A0A5F9CKA3	2	751.606	1214.52	1008.14			
A0A5F9CKB3	3	1115.78	1537.15	869.241	1522.04	1994.74	2252.33
A0A5F9CKC6	4	1211.46	1730.67	1078.87	1675.02	1289.24	1776.18
A0A5F9CKC8	1	504.578	864.404	952.82	1719.19	3400.63	2080.11
A0A5F9CKI2	2	4183.3	6310.2	3645.29	6837.11	3067.56	6302.97
A0A5F9CKJ4	7	1744.73	1520.65	1904.49	946.415	478.197	949.836
A0A5F9CKJ5	2	6843.75	8395.86	2710.96	10021.4	12406.5	29004.2
A0A5F9CKR3	5	801.06	829.94	546.185	1108.24	1184.24	1039.41
A0A5F9CKU4	3	869.179	1767.84	1244.84	736.714	1496.49	588.693
A0A5F9CKV7	9	3850.12	3030.36	4079.18	1880.67	1973.82	2619.73
A0A5F9CKX8	1		276.13	211.472			106.952
A0A5F9CKY7	6	1542.97	1436.81	1872.84	931.444	1119.74	1016.68
A0A5F9CL26	7	555.085	726.766	804.657	1110.87	1085.07	975.886
A0A5F9CL28	1	6312.45	7005.68	3747.43	17316.4	22353.8	15373.1
A0A5F9CL34	11	2656.23	2580.2	2626.62	2504.61	2309.52	2335.23

A0A5F9CLA2	1	1659.42	1876.71	2086.57	1929.51	744.618	2002.22
A0A5F9CLC7	2	202.404	642.315	158.016	1020.19	1007.06	966.741
A0A5F9CLN2	16	5494.93	6149.46	6180.03	5031.08	4613.23	5296.67
A0A5F9CLR1	3	2745.38	2462.92	1681.23	1536.72	982.591	
A0A5F9CLR3	2	964.771	987.105	708.929	465.071	692.777	490.581
A0A5F9CLR7	4	1578.26	863.422	1358.11	646.599	575.458	672.017
A0A5F9CLX5	5	1195.31	1236.22	1227.07	2270	2103.05	2309.9
A0A5F9CM01	5	2475.37	3435.73	3228.95	2353.09	2173.92	2559.1
A0A5F9CM08	2	948.212	1087.82	1224.48	807.558	721.291	922.46
A0A5F9CM15	4	2143.94	1414.1	1704.39	2318.15	2485.91	2686.44
A0A5F9CMB7	9	328375	318459	327281	397634	350533	448024
A0A5F9CMD7	10	1996.72	2196.83	1706.03	3162.98	3587.74	3106.68
A0A5F9CME5	2	396.015	588.757	449.683	409.907	388.031	
A0A5F9CMJ3	9	2163.37	1866.47	2106.88	1278.77	1185.84	1285.19
A0A5F9CMJ6	6	2128.7	5549.7	2818.52	4798.75	4208.57	5634.82
A0A5F9CML0	13	21811.6	24783.2	21411.2	50524.3	45934.6	50562.3
A0A5F9CMN2	3	838.546	630.901	542.742	1155.64	1146.66	1253.19
A0A5F9CMN9	5	1512.06	1577.27	1321.85	2696.92	2379.9	2803.21
A0A5F9CMS4	1	227.179	202.494	266.289	543.897	308.734	
A0A5F9CMU1	17	3498.76	3851.26	2944.12	2640.73	3061.17	2968.77
A0A5F9CMV1	4	5226.95	4406.44	5164.01	2906.54	5358.86	4156.82
A0A5F9CMY3	4	792.879	316.591	574.328	796.986	499.539	712.026
A0A5F9CMZ2	5	2038.25	2551.52	1642.3	1653.58	2369.75	1373.61
A0A5F9CN14	1	413.163	328.538	576.502	714.669	167.93	204.024
A0A5F9CN32	3	735.28	895.076	774.959	642.371		507.419
A0A5F9CN45	1	1065.24	956.572	468.255	789.935	339.776	580.271
A0A5F9CN83	6	2575.79	2320.41	1616.57			
A0A5F9CN85	6	3327.51	8266.1	2397.58	4424.29	7803.49	4579.89
A0A5F9CN86	13	5745.46	5993.01	5449.38	7239.37	6408.73	6326.11
A0A5F9CNA6	1	589.279	796.549		1483.73	2608.64	1594.54
A0A5F9CNB1	1			181.089			
A0A5F9CNB2	14	9514.71	16713.1	7186.01	14174.7	13482.9	15329.7
A0A5F9CNC6	9	1629.28	1331.06	1779.75	689.427	931.989	575.226
A0A5F9CNE1	1	2186.07	2656.67	2173.12	2283.28	906.478	1669.67
A0A5F9CNG0	1	2177.66	1545.78	1618.54	1209.36	1621.92	1396.14
A0A5F9CNH0	4	1801.5	1861.22	2115.92	1507.71	1574.82	1357.9
A0A5F9CNK3	3	30882.3	40797.9	19176.8	32172.7	33505.1	33584.6
A0A5F9CNU0	23	6667.57	5587.37	5459.75	7191.89	6083.87	6944.05
A0A5F9CNX4	5	8591.44	16847.5	8119.51	19942.1	18194.9	17009.1
A0A5F9CNX5	2	2293.26	1636.38	1827.85	1509.7		1415.56
A0A5F9CP06	14	4340.57	4998.48	3834.67	2829.88	3004.68	2344.18
A0A5F9CP20	1	198.233	403.316	215.875	335.812	1132.88	1012.31
A0A5F9CP21	12	1963.15	1762.67	1971.34	901.85	993.573	975.073
A0A5F9CP22	2	1709.9	1742.39	1000.36	1578.93	1502.4	1620.89
A0A5F9CP99	2	790.336	1153.52	920.149	952.381	1307.94	593.439
A0A5F9CPG4	2	585.417	1085.45	896.102	808.317	360.081	535.811
A0A5F9CPG7	1	2236.83	1950.5	1553.54	948.71	1595.92	844.215
A0A5F9CPH0	2	1145.9	1006.38	739.441	923.048	642.259	855.073
A0A5F9CPI5	7	1146.43	1225.82	1188.41	1358.09	686.554	691.639

A0A5F9CPJ2	3	1845.49	2321.59	2582.17	1359.35	1116.13	1661.28
A0A5F9CPK4	17	36757.5	30309.1	40749	72479.1	77243.3	81372
A0A5F9CPL0	1	2265.54	1718.96	1718.72	779.49	620.345	949.145
A0A5F9CPL9	2	1367.89	1378.16	1700.54	976.22	927.54	1062.55
A0A5F9CPP3	3	1333.87	1050	868.519	1578.97	1398.9	1097.48
A0A5F9CPU6	3	1325.75	1334.76	903.278	652.318		683.256
A0A5F9CPW9	5	852.974	1192.06	1047.78	1377.84	855.852	1008.99
A0A5F9CQ04	2	754.435	1059.89	872.69	1472.5	1290.12	1749.77
A0A5F9CQ27	29	4264.84	4816.29	4399.8	2171.11	2636.07	2192.49
A0A5F9CQ79	3	2428.3	2585.72	1724.15	2004.09	1967.04	1976.83
A0A5F9CQC5	4	2294.95	2552.74	2052.45	1822.04	1080.1	1333.26
A0A5F9CQD4	17	6041.01	5746.64	6337.64	4076.54	3578.17	3717.61
A0A5F9CQG3	1	761.022	613.148	175.993	932.258	1372.59	1073.78
A0A5F9CQH1	2	1852.05	673.009	1687.72	1944.99	879.064	1182.41
A0A5F9CQH5	3	3877.6	2587.91	4252.54	607.942	662.047	444.489
A0A5F9CQK5	27	5012.3	4425.22	5475.09	3373.28	2669.68	3193.56
A0A5F9CQK7	3	6948.61	4747.98	14581.8	8365.22	7989.2	11713.6
A0A5F9CQN2	2	989.105	649.912	727.602	895.955	823.052	1103.82
A0A5F9CQN3	9	2794.03	3565.66	2627.17	1996.75	2230.28	1622.67
A0A5F9CQV3	2	798.319	907.145	1058.47	626.581		445.397
A0A5F9CQX3	3	1231.26	1163.39	577.894	632.538	420.724	821.06
A0A5F9CR24	4	1539.38	1954.65	1115.82	2500.52	2642.55	2386.66
A0A5F9CR36	2	397.8	360.729	462.785	320.958		373.07
A0A5F9CR75	2	1941.77	1749.51	1928.76	2667.53	2199.55	2482.2
A0A5F9CRC2	2	981.297	554.659	523.708	899.58	725.304	1713.85
A0A5F9CRD7	10	3093.77	3617.95	2861.16	3900.73	3833.99	3527.35
A0A5F9CRF1	3	1591.58	4652.15	1454.67	5419.89	6629.24	4404
A0A5F9CRI0	5	1954.65	2557.9	2706.44	4239.15	2941.31	3596.02
A0A5F9CRJ6	1		750.316	531.825	1012.14	759.973	600.655
A0A5F9CRL1	7	7400.93	11229	5501.4	16193.5	21494.6	15650.9
A0A5F9CRL8	1	3064.03	1817.31	1176.59	12651.4	18008.8	10324.1
A0A5F9CRP0	2	32122.5	6486.53	54051.8	7676.23	1003.94	5857.59
A0A5F9CRP1	1	837.194	1076.67	630.806	1384.52	956.609	1283.26
A0A5F9CRP6	6	1651.74	1663.63	1634.73	3230.49	3275.4	2998.42
A0A5F9CRT6	1	1959.7	1317.62	1526.34	890.91		551.341
A0A5F9CRT8	6	1699.09	1101.59	1135.47	1122.17	1335.34	1071.54
A0A5F9CS59	2	818.699	636.832	680.136	613.894	504.985	609.934
A0A5F9CS97	10	42146.7	35745.3	43818.8	62997.4	57558.6	62678.9
A0A5F9CSB0	4	2267.55	2680.63	3197.84	3111.72	2226.59	4059.37
A0A5F9CSB4	4	1012.26	851.679	744.745	960.745	765.069	681.314
A0A5F9CSC9	4	4914.78	4570.6	6087.2	3365.45	2932.88	3324.72
A0A5F9CSH4	3	988.994	1020.98	803.382	1666.07	1031.24	944.998
A0A5F9CSM8	3	830.827	1250.41	577.941	677.635	788.248	881.752
A0A5F9CSQ9	25	622232	239509	911673	99501.1	94994.6	142484
A0A5F9CSU2	5	36153.5	53728.2	36538.3	83432	89366.3	74977.8
A0A5F9CSW3	1	1024.36	989.883	1166.58	716.09		458.569
A0A5F9CSX2	8	3425.44	4033.29	3826.95	2782.55	2554.91	2471.11
A0A5F9CSY2	5	4183.88	3128.53	4590.53	2449.21	1797.97	2328.93
A0A5F9CT28	13	4062.95	4255.41	4788.88	2948.04	3352.17	3131.51

A0A5F9CT34	1	226.734	252.501	331.175			
A0A5F9CT46	6	2842.51	2817.72	2050.41	2444.17	1695.84	1770.23
A0A5F9CT68	35	2859.58	2859.95	2691.01	1586.88	1942.52	1626.54
A0A5F9CT93	64	68794.2	86845.6	61157.5	119693	113120	115626
A0A5F9CTF7	1	223.681		307.209			
A0A5F9CTG0	2	1169.56	678.678	966.501		208.488	651.766
A0A5F9CTG1	2	419.605	729.805	756.284	1481.48	970.396	1224.44
A0A5F9CTH8	5	2091.75	1688.41	1858.3	1066.56	917.344	791.372
A0A5F9CTQ6	1	134.305	318.969	351.656		233.285	
A0A5F9CTS6	4	1686.02	4720.27	1473.18	7819.82	11234.5	6468.48
A0A5F9CTU0	5	23782.8	20129.4	19824.1	10653.5	11005.2	12544.1
A0A5F9CTV6	13	3620.04	3002.03	2854.84	3922.52	3042.28	3647.06
A0A5F9CTW0	9	7937.88	8107.26	5757.6	5707.91	5143.77	6064.89
A0A5F9CTZ4	1	1228.7	1754.85	747.796	1201.81	1388.34	1127.66
A0A5F9CU21	21	5979.57	7794.66	5819.1	5387.51	6115.76	5673.03
A0A5F9CU78	9	2273.37	1968.59	2483.55	4193.58	3594.51	3628.22
A0A5F9CUC6	18	6237.8	8506.63	6875.22	4921.16	4476.81	5312.37
A0A5F9CUH2	8	3414.58	4480.86	3112.44	3055.36	3273.3	2637.17
A0A5F9CUM5	2	18923.8	18629.9	22609.2	50942	39609.9	52132.1
A0A5F9CUP3	3	1284.11	1216.56	1557.09	757.469	1096.07	1311.28
A0A5F9CUS4	6	1938.34	1532.71	1347.71	2616.65	2224.67	2085.95
A0A5F9CUT2	4	2195.23	2423.68	2149.71	1398.53	1064.81	1977.92
A0A5F9CUZ4	1	3459.2	2373.68	4230.69	4189.73	2343.86	4886.21
A0A5F9CV54	1	762.961	634.387	929.001	1822.82	1267.63	1960.55
A0A5F9CV64	3	1071.06	578.621	908.093	892.056		961.663
A0A5F9CV98	59	282917	255981	275539	91168.4	93423.6	101382
A0A5F9CVB7	7	3192.1	2574.72	1988.5	5125.42	4673.05	5670.24
A0A5F9CVH3	2	10145	12429.2	4708.71	8145.14		7708.68
A0A5F9CVI6	4	1322.98	1321.26	2309.58	708.671	1180.5	1028.98
A0A5F9CVK6	1	359.804	1531.45	456.753	3868.74	4183.26	1855.23
A0A5F9CVL8	1	3793.72	2353.82	5912.87	6431.79	7368.31	13635.6
A0A5F9CVM9	8	4927.9	4733.87	4691.73	3030.4	3084.53	3002.79
A0A5F9CVR5	1	1302.87	1941.61	984.344	1761.16		1528.88
A0A5F9CVV7	4	2059.57	1569.14	1696.07	1332.5	918.628	1023.11
A0A5F9CVX6	1	4905.25	7060.89	1933.3		2493.74	2233.52
A0A5F9CVY7	4	1248.26	1361.48	1312.57	1718.82	1524.82	2050.31
A0A5F9CW40	1	874.564	888.009	561.994	398.003	603.269	597.971
A0A5F9CW42	1	17345.4	12572.5	10905.3	14765.8	14132.7	16474.2
A0A5F9CW53	3	1024.55	865.636	782.807	739.55		790.796
A0A5F9CW65	1	244.953	745.982	323.394	903.299	390.308	321.525
A0A5F9CWF8	3	1059	1154.63	1340.9	3232.28	2606.6	3763.71
A0A5F9CWI4	3	590.359	1207.28	632.429	949.488	856.111	761.755
A0A5F9CWI9	2	849.173	1014.42	726.706	809.237	764.814	1098.88
A0A5F9CWS3	2	937.031	635.427	1106.24	581.466		547.787
A0A5F9CWS4	5	3950.18	3509.02	5204.04	2780.79	2049.83	2434.75
A0A5F9CWT4	13	26571.4	20507.2	26388.7	42285.2	29411.5	38822.7
A0A5F9CWV3	6	1871.24	2223.63	1758.01	1630.71	1760.54	1294.48
A0A5F9CWY2	8	1189.82	1245.76	1329.79	1053.98	1171.65	849.062
A0A5F9CWY4	5	1067.44	867.403	938.421	2238.03	1743.36	2033.14

A0A5F9CX05	10	1566.87	3030.64	1511.82	1579.82	1634.83	1669.58
A0A5F9CX84	2	4132.06	3707.06	4094.3	3108.75	2843.23	2921.61
A0A5F9CXC0	4	1594.64	1923.67	1742.46	641.182	1490.48	1207.93
A0A5F9CXG8	2	657.075	1136.14	1215.65	795.292	411.264	362.342
A0A5F9CXN0	17	9814.61	13656.3	9732.56	8464.64	8883.13	9847.23
A0A5F9CXT1	3	1657.21	1259.13	1350.69	2460.76	1962.55	2227.22
A0A5F9CXT8	17	5895.26	5819.67	5463.18	4149.97	3580.71	3957.41
A0A5F9CXU6	6	1321.31	1354.98	1335.53	1210.3	1199.81	826.704
A0A5F9CXV0	1	904.088	336.803	546.528	458.068		817.944
A0A5F9CXW0	15	2994.37	2874.77	3681.28	5255.77	5421.88	5645.39
A0A5F9CXZ1	4	962.653	938.207	750.91	1221.9	760.808	901.166
A0A5F9CXZ6	18	42590	42630.8	23053.9	16641.2	15751.1	17876.3
A0A5F9CY06	2	520.696	543.342	499.253	245.633	158.73	541.684
A0A5F9CYP3	24	79043.2	108704	32419.9	23866.6	24934.4	24149.3
A0A5F9CYQ1	14	3174.27	1876.54	2793.04	1390.3	1677.01	1118.28
A0A5F9CYS1	1	505.254	333.164	344.847			
A0A5F9CYU5	3	782.76	745.068	660.534	1032.41	524.211	699.591
A0A5F9CYX6	1	30721.6	9779.15	32974.4	21482.1	3701.13	19441.3
A0A5F9CYY5	143	350804	411466	357941	613391	616011	630842
A0A5F9CZ06	2	3910.92	6539.94	725.457	8358.19	9019.2	7579.98
A0A5F9CZ37	3	652.524	718.331	392.243			
A0A5F9CZB0	10	5802.02	7024.49	4627.76	4030.86	3257.93	3892.77
A0A5F9CZB6	12	2161.07	2369.08	2443.4	1594.58	1404.1	1349.06
A0A5F9CZE1	7	1955.16	1350.64	2016.72	1309.59	693.821	1194.06
A0A5F9CZI2	1	648.112	650.365	1082.04		358.657	562.552
A0A5F9CZI7	39	12199.5	13288.5	12630.6	10982.5	9670.88	10712.9
A0A5F9CZN0	3	887.292	811.435	1033.96	928.723	674.495	422.708
A0A5F9CZQ7	1	1028.98	1184.36	615.979	450.166	697.226	291.106
A0A5F9CZR3	3	818.569	1015.59	1094.27	958.767	830.375	638.702
A0A5F9CZV1	10	5767.53	6597.25	7092.38	4775.55	4215.5	4712.81
A0A5F9CZX5	5	1476.07	2320.63	923.419	2176.94	1528.7	1269.69
A0A5F9D028	2	7266.67	8065.77	4518.47	10085.6	11458.1	15464.8
A0A5F9D053	2	760.469	935.693	1438.06	446.999	488.602	1032.57
A0A5F9D0A4	2	1004.83	1132.56	1631.66	1320.68	1661.66	1633.6
A0A5F9D0B6	3	1005.03	880.027	835.098	634.29	600.214	951.683
A0A5F9D0H4	2	3352.52	3966.19	1702.81	2470.72	2982	2152.67
A0A5F9D0R0	3	1043.35	1058.26	1119.9	856.697		682.237
A0A5F9D0T9	37	58326.1	71680.3	64613.3	116403	121038	120449
A0A5F9D0X4	4	1282.14	1187.5	910.854	1750.2	1912.48	1348.07
A0A5F9D104	6	629.913	686.337	851.131	1418.18	1553.6	1453.79
A0A5F9D112	3	347.878	983.975	530.82		709.338	488.202
A0A5F9D128	4	1782.93	2203.45	1522.6	1478.41	2042.11	1502.55
A0A5F9D1H7	2	1426.96	791.633	1444.53	1086.17	858.176	437.38
A0A5F9D1J7	32	17856.3	17809.5	18875.4	9343.65	7712.96	9570.58
A0A5F9D1T0	1	1049.42	1553.47	462.545	1823.38	2171.84	1396.08
A0A5F9D1T9	17	2649.48	3246.34	2874.32	1996.01	2155.78	2154.67
A0A5F9D1V0	3	2699.94	2989.33	2690.83	3097.97	3551.14	2305.07
A0A5F9D1W7	1	17969	12001				17022.1
A0A5F9D217	14	5054.92	4217.67	3024.5	3514.51	3392.98	4799.45

A0A5F9D221	1	649.78	644.747	748.09	766.881	631.973	888.649
A0A5F9D230	1	2887.98	1000.96	1949.78	666.799	941.006	721.764
A0A5F9D249	2	1123.06	897.501	1324	561.165	881.287	774.955
A0A5F9D252	3	3198.16	2920.51	2985.82	1701.41	1209.83	1473.65
A0A5F9D253	12	6339.76	9102.86	3442.61	1489.15	1985.82	1310.63
A0A5F9D287	32	65124.8	65808.7	65495.6	44196.1	43241.3	50448
A0A5F9D2C7	15	4621.91	5076.36	4575.62	3515.88	3269.17	3418.28
A0A5F9D2F7	2	976.515	1287.58	1231.46	1767.14	2186.45	1513.98
A0A5F9D2J2	10	3676.09	5233.12	2321.35	4359.08	4465.62	3547.94
A0A5F9D311	9	1492.93	1325.85	1295.86	962.232	1244.36	911.343
A0A5F9D360	1	10891.5	15509.8	6672.86	21510.2	21278.7	23650.6
A0A5F9D363	5	15275.2	15005.6	15125.1	9201.28	10265.7	9493.26
A0A5F9D376	8	2269.01	1958.09	1788.04	2007.58	1693.21	1577.03
A0A5F9D3F9	1	701.758	796.467	524.504			
A0A5F9D3H1	3	1946.68	1507.86	1710.97	1026.37	1114.94	972.11
A0A5F9D3K5	1	1021.97	1131.66	1110.69	741.955	592.809	440.454
A0A5F9D3M6	1	545.594	786.104	1048.7	2910	3393.51	3928.16
A0A5F9D3N8	15	2076.69	2074.49	2084.49	3000.97	2398	2700.68
A0A5F9D3P7	37	16897.3	18234.9	14839	69787.5	77636.4	80122.4
A0A5F9D3Q0	3	1289.55	1210.86	1065.33		1026.08	902.241
A0A5F9D3V9	1	936.323	892.816	591.651	601.351	576.143	817.42
A0A5F9D3Y0	6	1671.12	1678.07	2089.89	1596.81	1689.15	2147.82
A0A5F9D432	3	910.886	1300.58	1126.01	672.103	732.5	725.634
A0A5F9D445	3	924.244	610.353	1029.43		544.268	630.278
A0A5F9D454	2	2384.08	2277.88	832.002	1510.58	847.893	1076.43
A0A5F9D479	3	1698.41	2137.34	1981.04	1546.77	817.689	855.859
A0A5F9D483	27	9231.45	8728.9	8546.35	8124.22	7562.17	8224.9
A0A5F9D4G4	8	1360.87	2203.95	1158.41	1465.48	1358.64	1615.09
A0A5F9D4H9	2	1399.31	1902.33	2167.99	1653.21	1537.94	1402.23
A0A5F9D4Q1	2	1240.15	1317.34	1925.64	803.616	1593.35	866.829
A0A5F9D4Q9	1	136.199	475.767			383.738	
A0A5F9D4S1	18	2849.48	6098.38	2822.83	5520.88	7186.27	5034.76
A0A5F9D4S9	3	615.829	1394.5	557.882	882.461	978.027	847.176
A0A5F9D503	2	1135.55	1481.01	1516.68	817.937	1363.1	958.831
A0A5F9D568	1	816.982	749.482	576.057	1048.72	946.336	1033.16
A0A5F9D585	30	40516.8	39498.6	42184.2	67746.3	68738.8	75907.9
A0A5F9D587	1		1191.9	241.65	1586.72	1894.84	2004.44
A0A5F9D5A6	19	4223.87	3680.59	4696.94	588.671	980.604	878.318
A0A5F9D5B4	2	824.593	840.229	885.997	985.12	1126.94	844.176
A0A5F9D5C2	14	2145.22	2847.5	2477.63	1657.81	1913.51	1363.3
A0A5F9D5D2	2	1279.86	715.666	626.034	763.669	657.785	692.389
A0A5F9D5K0	2	378.664	713.919	592.617	913.032	772.324	660.917
A0A5F9D5N6	2	1567.5	1082.9	1099.34	708.691	728.384	822.93
A0A5F9D5Q4	2	1868.25	1347.48	1740.58	1477.16	522.273	872.061
A0A5F9D5S5	1	22226.5	67790	9726.58	13450.8	16463.3	16027.4
A0A5F9D5U6	2	369941	324234	425011	285746	95862.4	314358
A0A5F9D5Y6	7	1903.92	1464.21	1902.66	1291.33	765.98	1064.61
A0A5F9D5Z3	3	8698.53	4725.74	4378.11	11902.5	12386.2	12685.8
A0A5F9D5Z5	11	7192.19	8633.37	7988.97	7317.6	6193.2	6851.35

A0A5F9D603	1	36068.6	32166.6	36440.5	44939.4	31931.6	56563.5
A0A5F9D613	15	142919	201760	148867	136491	138173	142148
A0A5F9D683	39	16975.1	14795.1	16622.5	8468.93	8234.17	7994.58
A0A5F9D6B2	5	1977.56	2646.83	2703.45	1573.16	2244.19	1358.24
A0A5F9D6E4	2	1687.88	1063.63	751.616	1149.45	1124.74	
A0A5F9D6U2	7	3743.47	3129.19	4493.7	4809.19	5372.99	5294.17
A0A5F9D6U8	6	5066.36	5505.99	4197.33	2247.69	2633.25	3299.65
A0A5F9D6W1	19	4887.07	4680.53	3240.99	575.892	1240.44	506.115
A0A5F9D6Z2	4	671.378	895.391	745.153	812.517	449.034	723.022
A0A5F9D6Z9	1		518.989		667.257	1220.77	1180.19
A0A5F9D748	1	758.188	672.557	635.341		672.013	909.594
A0A5F9D756	7	24506.4	27507.4	17684.5	17668.2	21548.3	18787
A0A5F9D7D4	1	2078.77		1469.33	6996.53	8502.44	5766.04
A0A5F9D7Q7	13	3189.61	3442.72	3414.48	1971.79	2206.95	1794.17
A0A5F9D7S4	1	594.952	941.241	1126.28	644.048	653.564	715.782
A0A5F9D7W0	2	680.606	761.945	630.042			308.131
A0A5F9D7X0	1	190.532		238.447			360.751
A0A5F9D7Z0	4	890.723	618.813	748.61	373.108	364.49	256.209
A0A5F9D808	1	629.467	1441.07	556.081	1896.06	1890.96	1413.83
A0A5F9D8E1	4	15264	18147.8	14738.7	16430.6	57555.5	19411.6
A0A5F9D8G2	1		213.269	392.357			
A0A5F9D8G4	1	216.19		891.73	403.338		374.636
A0A5F9D8K0	21	17669.5	17665.9	9002.37	21958.4	31631.5	83465.2
A0A5F9D8N2	4	1484.66	1664.79	1829.12	1506.36	1235.11	1153.9
A0A5F9D8R7	7	2135.66	1655.31	1868.6	936.56	834.998	823.709
A0A5F9D8S4	1	11108.1	11605	4349.53			
A0A5F9D8U1	3	1382.86	2169.61	880.066	2792.62	2953.18	2621.02
A0A5F9D8U7	17	2531.71	2417.81	2938.83	1474.7	1608.65	1415.5
A0A5F9D954	12	1892.99	1824.1	1670.33	1695.89	1784.56	1693.19
A0A5F9D9E3	4	9777.48	10022.1	8836.99	12383.3	12304.9	11287.1
A0A5F9D9E9	5	1964.23	1586.43	2254.03	2805.15	1562.17	2509.52
A0A5F9D9H1	15	1634.61	2314.05	1683.59	1512.65	1310.53	1479.95
A0A5F9D9U4	5	1397.7	940.733	1488.56	925.944		863.207
A0A5F9D9X0	3	1339.46	1641.53	1448.14			453.12
A0A5F9D9X3	3	4212.33	4857.51	3475.46	4587.84	4427.83	4455.48
A0A5F9DA40	1	832.656	763.32	869.963	336.176		
A0A5F9DA41	12	10765	10482	9761.79	9121.74	9040.87	8743.7
A0A5F9DA47	1	1059.56	688.451	843.8		532.642	897.663
A0A5F9DAC7	2	324.955	1007.02	531.955	1500.94	1150.12	1918.38
A0A5F9DAI8	1				3706.75	4532.54	3484.08
A0A5F9DAK7	2	621.449	553.361	792.325	1657.59	1547.03	1217.11
A0A5F9DAQ1	10	3044.66	2461.23	2876.82	1475.4	1388.41	1468.42
A0A5F9DAQ2	33	9198.02	8340.53	9898.1	18142.6	16576.4	16909.5
A0A5F9DAT4	14	3630.91	3017.21	3735.33	2340.1	1881.93	2229.6
A0A5F9DB45	13	16497	16942.2	15161.1	11896.3	12785.2	12237.3
A0A5F9DB61	6	4201.58	3798.16	4410.15	3442.89	3820.83	3619.48
A0A5F9DB70	44	41126.7	37560	46429.4	71736.5	66986.3	67004.3
A0A5F9DB73	2	348.842	403.152	600.523	1026.78	775.697	514.516
A0A5F9DB91	3	797.511	626.759	1041.16	812.324	471.514	423.629

A0A5F9DB98	4	21736.6	9012.76	21118	25538.5	24271.5	30280.4
A0A5F9DB99	1	751.742	605.821	918.631	3091.58	2377.87	1247.13
A0A5F9DBB2	8	1008.55	786.537	846.809	623.832	892.624	1057.82
A0A5F9DBB4	11	4308.99	5961.59	3821.82	4764.78	4263.47	4818.53
A0A5F9DBD2	5	697.961	1087.63	1427.33	2587.33	1949.71	2886.65
A0A5F9DBJ1	1	22408.6	35251.4	15243.7	7449.69	9764.72	14855.4
A0A5F9DBL7	1	7371.91	15650.4	1900.69	10184.8	15766.9	10719.2
A0A5F9DBQ8	15	3292.43	3143.06	3295.45	3444.57	3065.86	3426.1
A0A5F9DBR5	11	7047.62	5842.05	6134.93	11106.5	9069.04	12318.5
A0A5F9DBT6	3	1060.34	1011.24	1262.14	1238.11	1059.32	1042.74
A0A5F9DBU5	1	1155.21	1640.74	897.665	1011.4	1128.72	925.039
A0A5F9DC16	1	881.684	561.038	1560.41	1101.25	1078.66	1402.58
A0A5F9DC47	1	2028.94	1624.84	1830.29	1659.05	1213.64	1572.68
A0A5F9DCA8	2	631.689	553.102	772.253	523.948	328.102	333.067
A0A5F9DCD4	67	21236.5	23185.7	22797	9580.11	9284.88	9147.85
A0A5F9DCG0	33	13933.7	12942.9	14742.2	19780.9	17609.4	19468.3
A0A5F9DCJ0	1	2104.79	2033.7	1268.3			414.71
A0A5F9DCK4	12	4275.67	4177.19	4658	3058.99	2798.16	3188.44
A0A5F9DCQ3	1	16474.3	11414.4	15928	5168.55	5620.44	5029.17
A0A5F9DCX4	15	1696.21	1414.07	1740.19	1011.58	991.013	1100.96
A0A5F9DD89	1	308.674	1155.62	417.308	740.271	904.693	555.843
A0A5F9DDA1	3	20140.4	25049.6	22228.7	40471.4	46595.1	47311.6
A0A5F9DDB1	1	43346	47305.9	103935	29906.5	19621.5	29743.7
A0A5F9DDP6	3	1049.54	805.658	948.925	991.552	592.199	733.634
A0A5F9DDT0	2	938.717	171.01	356.474			173.027
A0A5F9DDT5	13	2542.24	2291.65	2525.92	1835.58	785.4	1355.96
A0A5F9DDV3	2			735.888	473.745		893.013
A0A5F9DDZ7	74	25045.5	37134.6	13678	1594.56	2322.69	1918.46
A0A5F9DE32	13	4612.4	5314.75	4304.41	4031.42	2973.27	3610.18
A0A5F9DE53	5	1998.87	2882.38	2064.73	2648.6	2436.61	2380.67
A0A5F9DE89	29	37011.2	30759	32033.8	85387	80987.5	87523.8
A0A5F9DED5	4	748.032	612.16	605.521	962.788		794.378
A0A5F9DEE6	1	312.96	423.99	132.034	572.772	479.234	501.9
A0A5F9DEF9	12	8317.14	7951.23	9395.77	8193.2	8867.74	7572.97
A0A5F9DEK2	11	8836.75	8406.5	8713.5	6349.56	5700.28	5713.51
A0A5F9DEL1	3	36442	39631.4	32654.2	67970.8	102941	88657.3
A0A5F9DEN1	22	21016.7	27790.9	14006.9	9801.98	9388.16	9021.67
A0A5F9DES0	2	347.704	464.866	603.357	322.006		241.302
A0A5F9DF00	4	941.548	2242.25	1502.46	3105.33	2915.49	3262.06
A0A5F9DF82	8	223941	197113	244587	218591	214466	223683
A0A5F9DFH2	2	918.802	1192.08	1331.83		1195.57	981.833
A0A5F9DFI2	8	10404.7	9101.42	11096.4	255.783	720.831	731.956
A0A5F9DFI8	25	97070.8	155864	63799.9	71971.7	85683.2	76697.3
A0A5F9DFJ9	1	16446.7	23732.5	2524.82	17694.8	13794.5	14924.7
A0A5F9DFL7	2	983.048	905.139	648.499	529.177	784.371	645.22
A0A5F9DG82	2	3861.53	3111.3	2255.56	2508.78	2009.85	2081.12
A0A5F9DGE4	40	50954	47648.6	42171.9	48770.3	49527.7	50285.8
A0A5F9DGF1	1	3813.11	10258.1	2903.23	14892.5	25131.9	14208.6
A0A5F9DGK7	4	1307.36	941.724	1349.13	2037.26	1504.83	1853.89

A0A5F9DGL8	1	1202	2108	1815.25	2597.34	2989.8	2615.86
A0A5F9DGN7	18	3835.83	3833.2	4122.94	9433.23	7093.05	7164.22
A0A5F9DGS3	1						471.463
A0A5F9DGZ2	11	2006.34	1340.04	1620.53	3932.9	4285.86	4021.15
A0A5F9DH17	3	7148.55	8976.08	4177.67	8651.79	9803.85	7167.24
A0A5F9DH52	13	1728.12	1946.44	1308.18	1468.15	1598.82	1481.84
A0A5F9DHN3	5	1544.78	1694.47	1393.77	890.369	944.211	918.946
A0A5F9DHU0	8	4010.34	3544.88	3460.88	5922.98	5655.96	7027.5
A0A5F9DI86	3	2085.21	1385.2	2860.04	1623.28	1515	1769.69
A0A5F9DI97	11	22865.8	39180.5	22197	44039.9	44217.1	43084.5
A0A5F9DIE2	4	937.736	1037.46	848.894	2847.5	1140.09	2420.42
A0A5F9DIH2	1	33203	110633	23354.7	118701	168662	122189
A0A5F9DIQ9	5	1051.66	1341.78	1280.22	3837.99	1435.28	1813.17
A0A5F9DIW3	3	1024.07	605.763	806.755	582.327	329.309	568.683
A0A5F9DIY4	11	4305.75	3834.09	3789.67	2072.75	2808.72	2525.02
A0A5F9DJG1	24	30475	26492.1	31631.9	20113.7	16832.3	20545.1
A0A5F9DJG6	22	16884	19490.4	15889.7	27096.6	25371.2	31616.9
A0A5F9DJP3	1	578.686	1356.22		575.176	943.829	999.075
A0A5F9DJR0	18	28595.5	28399.2	31718.8	38287.9	36841.3	40032.5
A0A5F9DJS7	1	596.832		770.868			
A0A5F9DJT8	13	8355.87	10218.5	9477.19	17552.3	18279.6	19606.7
A0A5F9DJW2	4	3921.09	4953.46	3431.95	3277.77	2207.71	2393.92
A0A5F9DK70	1	7024.21	7251.83	8856.61	6713.18	4476.31	9248.66
A0A5F9DKF7	1	1027.32	763.647		785.079	989.359	857.934
A0A5F9DKH0	1	682.495	752.645		1076.46	2226.95	685.561
A0A5F9DKI8	7	371286	301172	282941	212920	251775	186418
A0A5F9DKQ7	1		321.318	355.718			
A0A5F9DKV6	2	957.893	1465.46	572.37	714.351	1655.32	1069.18
A0A5F9DKW1	13	3147.07	3260.16	3104.56	1788.16	1939.95	1672.36
A0A5F9DKX1	5	927.948	766.386	954.424	786.994	999.781	939.067
A0A5F9DKZ1	10	2771.42	2975.54	2755.5	3708.92	3262.61	3845.09
A0A5F9DL41	10	2970.75	2692.27	3687.76	5640.86	6290.32	6241.67
A0A5F9DL63	1	628.431	1066.83	809.044	596.297		398.854
A0A5F9DL81	1	2654.08	1993.33	2781.26	1999.74	3723.96	5092.83
A0A5F9DLC0	1	192159	136034	160086	58078.1	59927.7	54566.5
A0A5F9DLC9	1	52693.8	72755	48236	11209.7	10072.2	14668.5
A0A5F9DLF3	1	1889.82	3473.08	452.557	1843.46	1625.45	2519.34
A0A5F9DLR1	1	2904.83	2421.85	3111.16	5281.88	3510.61	3889.87
A0A5F9DLR9	12	3951.52	5901.98	3307.13	3423.87	3382.17	2368.56
A0A5F9DLZ1	1	2899.78	2074.4	4962.89	2025.39		
A0A5F9DM68	2	680.875	401.118	514.621	535.335		567.239
A0A5F9DM87	12	2636.76	2201.38	3030.06	1157.43	1207.39	897.889
A0A5F9DMG2	11	16527.3	15079.3	17463.5	21946.1	21181.1	20912.4
A0A5F9DMU0	4	1080.68	1188.07	1331.19	862.175	1106.5	631.28
A0A5F9DMU6	2	3393.1	5357.35	3972.26	3590.35	3214.92	3492.3
A0A5F9DN85	2	10186.2	12886	9306.13	18080.1	14932	11380.2
A0A5F9DNA1	17	2516.9	3124.5	1968.42	2368.38	2452.69	1756.67
A0A5F9DNH9	2	1170.62	951.828	1001.62		1285.11	800.216
A0A5F9DNI2	1	313.902	269.394	255.42			435.948

A0A5F9DNM1	1	1477.61	973.257	1203.69	1564.33	1764.02	1291.93
A0A5F9DNN9	8	4697.03	4243.02	3866.42	3037.01	2405.93	2769.91
A0A5F9DNQ1	4	4195.61	3570.6	2920.45	2344.48		1271.36
A0A5F9DNS8	3	1364.5	979.334	1398.23	726.32	821.193	971.013
A0A5F9DNT2	57	168733	156392	162344	235763	203641	221668
A0A5F9DNU0	8	2621.14	3218.74	3169.78	4178.68	4748.73	6401.49
A0A5F9DNW0	2		688.138	519.289	193.181	905.395	497.209
A0A5F9DNY5	2	629.433	870.129	856.843	282.299		721.879
A0A5F9DP48	1	1405.5	3019.09	1146.59	1953.32	2231.73	1362.82
A0A5F9DP68	20	70183.4	54437.3	84647.3	126622	129680	124807
A0A5F9DPC3	7	6117.78	6792.19	7126.69	5706.63	4374.22	4363.53
A0A5F9DPG5	6	1059.39	1199.46	821.713	698.087	1060.19	779.31
A0A5F9DPI7	7	4164.26	4205.08	3558.4	2968.59	2815.85	2982.05
A0A5F9DPK6	3	1129.78	847.634	539.501	1383.18	1127.07	965.002
A0A5F9DPL1	15	15803.8	15872.1	16374.1	12472.2	11550	12369.9
A0A5F9DPX6	1	2332.84		1365.59	2198.83	2163.24	
A0A5F9DPZ5	33	3204.16	3543.96	3243.83	2877.8	2542.44	2680.12
A0A5F9DQ59	5	1453.35	1715.48	1758.31	2292.25	2522.8	2597.83
A0A5F9DQB7	12	4871.13	5995.28	2245.17	2406.92	2529.07	2286.53
A0A5F9DQH9	3	1241.96	1020.88	1042.65	1079.89	1802.73	880.729
A0A5F9DQQ2	7	1705.74	2351.14	1961.08	1501.97	1746.17	1500.88
A0A5F9DQV7	2	1570.78	1128.57	1227.45	1257	817.015	1160.63
A0A5F9DQX8	11	3309.11	3310.72	3961.36	1884.01	1936.28	1891.38
A0A5F9DR44	3	4351.14	5587.2	2856.26	730.195	799.42	1212.72
A0A5F9DR62	3	3025.94	3041.23	2304.03	1868.01	1037.85	1690.51
A0A5F9DRA0	1	1329.19	899.369	708.204	466.942	675.118	944.586
A0A5F9DRG1	2	754.394	1024.75	871.012	284.617	1102.68	644.661
A0A5F9DRJ7	1			686.03			
A0A5F9DRL8	3	1056.68	1140.25	1182.4	331.31	732.44	425.69
A0A5F9DRL9	10	2211.72	2475.64	2431.42	1504.82	1298.88	1506.68
A0A5F9DRT0	5	701.459	635.914	891.94	1052.97	774.19	993.314
A0A5F9DRU0	77	87929.3	147283	51975.9	105837	121154	101080
A0A5F9DRV0	6	1476.95	1439.11	1454	881.697	828.854	1091.78
A0A5F9DRV6	1	595.333	602.429	230.489	603.848	579.237	461.797
A0A5F9DRW1	5	3689.28	1621.71	1363.61	15146.9	16382.7	10241.4
A0A5F9DS02	2	877.009	628.217	781.524	219.98	381.913	246.029
A0A5F9DS50	31	63746.4	55164.4	63944.7	72139.4	74701.7	81210.8
A0A5F9DSF0	2	1397.86	442.891	844.261			1782.69
A0A5F9DSL4	4	754.745	984.734	511.311	431.315		761.586
A0A5F9DSM1	1	901.388	1293.99	652.633	930.363	893.568	
A0A5F9DSS0	1	11043.2	9597.33	10041.3	15898.7	13668.1	20024.7
A0A5F9DT47	2	4800.84	8336.54	4575.91	4113.64	8088.94	4185.84
A0A5F9DT59	1	393.967					
A0A5F9DT65	1	2125.03	408.339		1719.37		
A0A5F9DTB9	1	947.477	621.089	844.594			431.13
A0A5F9DTD6	13	14359.6	19588.1	15219.1	9879.89	9471.61	9845.99
A0A5F9DTE6	1	765.888	820.719	705.656	1448.89	2042.85	860.978
A0A5F9DTH8	3	3285.52	2049.77	3716.86	1209.55	674.902	1096.37
A0A5F9DTJ7	2	328.407	255.995			185.463	

A0A5F9DTK5	20	36637.7	31885.3	36230.4	56866.8	49619.8	60322.1
A0A5F9DTN5	4	1743	1679.01	2483.93	1176.84	2367.38	
A0A5F9DTR5	2	441.147	479.524	323.634	388.355	303.667	
A0A5F9DTZ9	1	919.5	1463.83	1124.85		968.457	
A0A5F9DU56	9	814.82	1316.94	919.066	1078.46	1018.31	1056.42
A0A5F9DU83	2	322.773	428.989	622.828			
A0A5F9DU85	10	2415.21	2727.42	2291.59	1741.88	1469.93	1588.73
A0A5F9DUJ9	31	13716.4	12707.3	14332.3	6441.51	5362.53	6492.41
A0A5F9DUL5	2	1149.9	1286.21	2046.74	3328.89	1842.33	3054.66
A0A5F9DUM3	3	1477.72	1434.5	1138.35	1994.41	1425.03	1558.01
A0A5F9DV00	1	4937.68	4105.48	5356.63	5852.62	3659.54	5618.76
A0A5F9DVA7	10	2448.87	3547.77	2494.02	2778.21	2253.68	2416.55
A0A5F9DVF4	1	640.29	564.641	809.348		194.98	610.878
A0A5F9DVK3	2	675.959	520.934	695.654	1068.34	488.706	852.787
A0A5F9DVK6	1	472.58	611.627	600.609			
A0A5F9DVW5	11	131462	129917	152908	121017	104782	115605
A7LH84	3	1810.61	2034.58	970.57	2144.09	1712.49	2322.85
A7X8X3	8	7224.59	9209.92	6797.2	6763.33	5136.68	5949.08
B6S6L6	9	2.73E+006	877386	7.38E+006	1.71E+006	436434	1.47E+006
B6V9S9	36	24674.9	19153.1	26152.4	11412.8	11183.1	10689.2
B7NZD2	3	815.68	891.751	1297.73	945.365	1133.38	863.488
B7NZD9	1	23630.9	66643.7	11245.3	14072.1	11296.8	13516.5
B7NZF1	16	4086.94	3407.81	3202.03	4612.73	4634.28	4768.57
B7NZM0	68	1.12E+006	849456	1.01E+006	1.21E+006	1.08E+006	1.17E+006
B7NZM8	9	2690.55	3873.03	2708.3	2848.99	3897.74	2950.59
B7NZQ1	3	26806.6	27696.9	21280.7	15441.2	14065.5	14382.5
B7NZR4	1	668.808	840.13	1015.62	650.001	608.35	315.048
B7NZR5	62	28024.4	35217.3	28607.2	41421.9	37363.6	41318.2
G1SCE4	5	4832.02	3754.23	5068.6		2288.64	2262.22
G1SCJ7	3	672.906	592.538	501.796	536.814	575.684	679.988
G1SCJ8	9	18286	28174.7	9496.92	15926.3	16472.5	22493.4
G1SCK5	24	25627.7	26438.8	22552.9	26741.8	24164.7	27356.1
G1SCN8	30	13489.3	13435.9	17376.7	7053.94	5606.41	8004.94
G1SCP8	36	29531	27082.8	29618	21327.1	18697.9	21012.2
G1SCR0	4	1936.1	1528.48	1874.19	1226.36	794.451	943.969
G1SCR4	2	1689.21	1901.95	1595.95	1579.65	1281.53	1438.89
G1SCY3	8	974.97	483.233	675.497	562.826		413.075
G1SCY4	10	21316.7	26132	22587.6	17709	16555.1	17243.8
G1SD01	9	1124.67	1137.77	1255.37	958.83	540.886	884.278
G1SD02	9	2124.81	2390.94	2457.84	1844.02	1874.9	2108.73
G1SD38	1	1926.04	1514.75	1592.45	693.599	306.961	733.969
G1SD43	60	262252	206151	237314	572072	554687	587442
G1SD48	7	1984.54	2243.26	1466.57	2719.35	2876.96	2732.75
G1SD52	4	936.888	602.716	955.71	2018.54	1568.53	1946.11
G1SD60	2	679.468	805.142	514.591	876.637	740.218	1218.47
G1SD70	1	124.695	192.032	214.339	391.47	516.527	263.375
G1SD89	6	2574.8	2243.01	1975.97	6217.06	4875.25	5069.99
G1SD91	7	1643.99	1422.55	1618.94	1305.23	1119.29	1235.07
G1SD99	24	70533.6	71231	73717.3	39597.4	35001.9	39298.8

G1SDA2	10	7792.95	11289.6	5840.08	4983.67	6083.06	4749.5
G1SDA4	15	3301.7	3121.72	3449.77	2259.84	2616.67	2235.63
G1SDA8	26	72774.8	59154.6	78029	143802	121484	151835
G1SDD0	5	1759.38	1856.68	2230.21	3984.22	3216.27	4291.48
G1SDD6	27	37417.5	52371.4	24543.5	17887.4	15495.9	17156.8
G1SDG2	2	2976.66	2428.3	2565.21	540.41		
G1SDI1	10	1443.32	1640.15	1750.11	1140.55	897.906	1249.77
G1SDL3	12	2158.22	1873.55	1742.89	1434.3	1373.1	1359.04
G1SDN4	2	1030.65	1253.7	575.422	890.474	476.082	765.126
G1SDR2	10	41557.8	78307	35224.6	33343.2	39294.8	35592.5
G1SDT0	1	6761.73	10027.3	4223.77	6858.76	6664.28	5539.82
G1SDU8	1	1695.26	902.647	1070.03	1506.18	2068.05	2359.54
G1SDV4	2	542.218	325.39	814.652			
G1SE06	31	114093	138179	114026	126601	128369	133602
G1SE51	5	1205.58	1168.73	818.128	1073.04	654.198	929.674
G1SE56	1	243.176	285.025	174.059			92.1323
G1SE67	4	967.999	1126.42	1506.6	923.744	863.022	1081.41
G1SE95	112	68801.6	92447.8	52443.8	62360.5	64904.4	60225.6
G1SEB2	20	14399.2	19308.3	12672.6	27129.5	30195.4	27969.8
G1SEC6	4	3449.1	2813.89	2289.54	9171.06	5947.1	9006.88
G1SEF6	1	351.24	179.664		803.89	449.658	420.161
G1SEF9	8	1602.21	1694.98	1671.3	851.035	616.67	360.969
G1SEG6	2	1153.34	1045.95	1032.68	1820.83	1884.77	2006.65
G1SEJ4	18	7122.04	5286.14	7756.83	6584.03	5996.46	6946.13
G1SEK2	4	1899.78	1621.44	1309.47	935.72	1401.19	945.361
G1SEK8	32	1.29E+006	1.43E+006	804896	249002	258838	289064
G1SEL1	21	17070.9	20202.5	15334.9	11173.5	11248.2	12102.3
G1SEL4	25	21159.9	16054.5	23250.4	39246.2	32635.2	42344.6
G1SEQ6	4	2351.34	1801.88	1633.02	3104.17	3173.71	2987.07
G1SER2	3	2028.16	2672.65	2246.82	1607.79	890.917	1289.72
G1SER8	9	59071.5	120135	17343.1	14036.2	14385.1	14021.2
G1SEY0	5	14537.1	16669.2	13315	38811.1	35341	36677.2
G1SF35	1	599.478	415.147	608.998		264.544	705.784
G1SFD7	3	1018.54	833.188	1631.41	3493.47	2824.4	3215.45
G1SFE9	4	2057.99	1959.07	1645.63	1284.25	777.726	1093.4
G1SFG0	4	1209.07	577.833	613.874	1184	967.072	1249.03
G1SFH8	4	878.661	1225.9	1160.54	1274.88	1538.94	1058.25
G1SFJ9	1	251.373	331.284	343.175	191.913		223.62
G1SFN1	6	5089.24	9588.62	3943.13	5799.6	4489.45	4361.92
G1SFR3	18	6667.73	8869.37	7202.58	7617.03	8528.67	8193.24
G1SFS8	34	6420.37	5323.33	6657.41	3193.65	2776.81	3286.37
G1SFT6	2	2359.23	2078.14	1988.41	1620.76	1621.69	1483.21
G1SFV1	22	4752.06	4103.87	3500.37	5302.81	4633.97	5415.68
G1SFV7	25	5903.28	7723.32	6024.17	6329.5	5050.23	6170.43
G1SFW1	11	2560.92	2250.07	1751.95	2729.84	2752.35	3296.73
G1SFX7	3	1041.01	2434.18	867.556	2202.85	2059.65	2110.16
G1SG57	1		597.259	384.543	420.642	378.659	401.182
G1SG65	20	4796.92	5101.53	3832.35	2721.73	2717.13	2575.56
G1SG72	10	2278.11	2292.11	2195.85	1783.41	1350.88	1709.14

G1SGC0	7	1162.16	913.878	1293.99	1158.04	935.126	766.185
G1SGF8	59	123683	104227	120416	204164	195757	223308
G1SGM2	3	1026.22	801.424	1293.05	464.583	286.272	453.876
G1SGM3	3	1964.94	1781.64	3704.09	3305.66	3513.26	2471.93
G1SGQ2	8	101101	90882.7	108146	157062	150914	165459
G1SGU0	20	131382	135862	125847	148195	151175	164661
G1SGV9	14	21691.7	18864.5	19718.6	68829.1	62335.2	68998.6
G1SGW4	11	24014	20141.7	20377.8	65630.1	62192	60078
G1SGY2	7	2607.26	2368.2	2507.7	2277.67	2490.19	2174.42
G1SH05	25	88499.8	79386.7	85590.3	42734.6	42587.7	45609.1
G1SH09	1	519.397	939.69	720.479	533.123		
G1SH41	1			204.185			
G1SH58	19	20884.5	23913.2	22969.4	28404.5	24216.8	26375.5
G1SHD6	2	2242.54	1559.33	2538.67	2006.7		2152.37
G1SHF1	1	618.95	494.883	572.259	571.499		587.979
G1SHF9	2	2650.56	2543.49	1518.63	579.462	417.298	791.842
G1SHH0	2	971.379	882.387	1184.7	591.417	835.437	812.502
G1SHI0	12	2532.18	2074.52	3036.82	4926.32	4427.47	5603.45
G1SHI6	1		1565.99	503.747	1579.56	1641.25	1097.84
G1SHR3	5	3933.4	3809.44	3700.84	3862.23	3309.01	3386.4
G1SHS7	42	63504.1	60110.8	68127	33751.2	29395.5	36005.3
G1SHT9	3	1944.5	2372.26	2531.49	2964.25	2704.55	3610.1
G1SHV9	20	37273.2	34066.3	38300.4	81216.8	73267.4	80045.8
G1SHZ4	3	3693.4	3275	1263.83	4487.81	5966.56	11292.7
G1SHZ8	41	20276.9	20453.6	18474.2	11612.8	10914.6	10833.5
G1SIA4	3	1341.23	1301.51	1836.19	2301.82	2525.87	2267.07
G1SIB7	14	35427	27975.4	38771.5	35885.7	31377.9	35042.8
G1SIB9	54	51567.7	44814.8	56256.7	30992.9	32125	32274.3
G1SIE3	2	824.873	724.033	743.436			1560.9
G1SIE6	19	25093.2	25593.2	21770.2	22015.1	20354.1	24523
G1SIF2	5	1945.7	1986.96	1642.24	2940.29	2409.01	2721.95
G1SIJ2	4	1281.71	1083.33	1149.33	651.051	592.894	818.721
G1SIJ6	1	487.635	396.725	492.968	397.204	551.94	560.589
G1SIJ8	1	2325.1	1413.99	2985	1243.84	754.568	872.607
G1SIK0	64	867020	751847	628728	459028	470505	1.52E+006
G1SIL2	18	49332.4	53526	51179.5	81326.9	75942	75233
G1SIN7	7	1745.76	1508.91	1899.6	846.748	600.684	846.308
G1SIP1	9	2771.73	2435.84	2941.02	1236.3	1738.01	1380.38
G1SIT6	2	1420.29	1347.71	1155.48	685.378	552.2	732.894
G1SIV7	6	3278.69	3830.98	3399.54	2634.1	2533.88	2398.99
G1SIY5	4	1986.9	1959.07	2186.01	3368.74	2437.29	2360.29
G1SJ23	19	13236.5	12358.8	10594.2	7629.33	7937.24	8896.79
G1SJ27	2	440.059	514.659	676.3			405.407
G1SJ72	7	1519.7	1757.11	1279.97	1690.23	2211.03	2518.75
G1SJE1	9	2601.33	3499.96	2142.28	3100.45	3014.42	2578.99
G1SJJ2	3	1181.02	722.913	729.719	588.034		502.357
G1SJM1	13	74237.4	64531.7	62670.8	30807.1	35223.4	41387.9
G1SJQ2	12	4234.07	5247.18	4274.73	4196.19	3345.27	4160.74
G1SJQ4	41	8666.95	8541.75	7779.73	1992.76	2205.8	1809.01

G1SJQ5	2	4651.19	7876.64	3832.39	5083.51	4244.67	5449.29
G1SJQ9	3	920.508	810.086	714.476	361.757	346.753	921.882
G1SJW7	1		760.814	397.051	453.573	548.135	906.088
G1SJY8	13	4791.6	4447.26	4384.88	3423.91	3339.64	3598.16
G1SJZ4	4	2824.89	3263.76	2852.62	1381.33	1044.46	1091.36
G1SK00	19	4213.25	4178.98	5136.24	3252.4	2443.52	2851.16
G1SK63	6	2124.91	2636.29	1929.23	3786.14	3570.84	4067.53
G1SKE3	2	8044.24	14992.7	9673.01	16639.6	26015.8	58312.3
G1SKE7	15	7131.73	7254.68	8471.38	8687.5	8588.5	8959.41
G1SKF1	69	298548	266756	303523	268828	265668	297580
G1SKI3	1	17075	10670.5	16242.3	19224	14625.2	23765.5
G1SKK0	9	1850.66	1967.08	2434.06	908.733	1029.86	720.877
G1SKM2	32	8448.75	7459.42	8588.9	9461.98	7147.02	9091
G1SKP2	17	2467.21	2849.87	2452.48	1942.29	1555.49	1423.75
G1SKS0	4	2247.35	2997.12	2136.85	1474.76	1502.28	1763.6
G1SKV7	18	10313.3	8395.31	10523.6	6750.61	6561.96	6703.9
G1SL03	4	1999.78	2653.35	2248.59	2221.05	2155.91	2016.14
G1SL41	12	4538.69	3871.49	4502.67	14198.1	12253.6	14816.3
G1SL57	3	1944.48	3322.69	1909.86	1704.79	1001.8	1898.35
G1SL68	159	122798	131344	135313	74996.9	72637.2	73654
G1SL78	1	31621.9	32110.7	21910.5	54583	65822.1	46903.4
G1SLC2	8	3600.27	2874.88	3549.05	2123.72	1855.68	2226.68
G1SLF5	1		860.303				
G1SLF8	16	2335.05	2728.81	1870.05	1948.59	1399.2	1907.02
G1SLK2	7	3588.51	2685.69	3258.92	2146.57	1874.44	1748.29
G1SLQ4	21	18746.8	24372	16958.1	13642.6	11335.2	11880.2
G1SLS8	3	705.172	634.124	267.939	791.804	557.569	764.471
G1SLT4	2	2037.91	1556.73	1744.82	1802.46	1078.47	699.867
G1SLU6	2	754.664	793.252	541.117	2472.7	1530.12	1652.02
G1SLU7	14	4749	4785.31	5598.14	2777.15	2513.14	2993.21
G1SLW8	5	1615.87	2219.96	1100.66	981.837	768.815	808.119
G1SLZ8	1	529.157	462.701	517.858		237.669	178.899
G1SM05	6	29568.6	33931.7	25130.7	26857.3	23836.2	25918.7
G1SM52	4	1876.55	2014.3	1360.24	670.66	393.942	748.289
G1SM87	4	1850.62	2202.48	1573.96	2232.99	2198.79	2156.38
G1SM91	2	768.565	1066.76	1010.94	1019.75	1017.44	914.784
G1SMA9	1	36719.7	45072.4	37208	31375.4	29334.1	32575.5
G1SMB8	12	8350.8	9081.57	8856.72	5117.05	5139.65	5522.79
G1SME6	7	3493.39	3333.47	2698.7	2287.99	2230.39	1505.84
G1SMI5	2	767.415	755.125	1110.62	629.574		517.783
G1SMJ7	1	353.479	402.245	186.581	1136.68	619.043	912.413
G1SMM4	8	11618	10835.8	10565.1	20971.3	21998.5	19304.2
G1SMM5	4	2752.83	2970.21	2275.09	1562.07	1019.28	1629.63
G1SMM6	7	2972.23	2549.18	2881.19	6292.04	6337.71	7003.57
G1SMS3	19	9277.92	13811.9	8219.21	7731.16	8795.94	7393.02
G1SMS5	8	2784.21	2637.9	2553.26	2433.59	2037.15	2844.01
G1SMT7	8	3012.87	3859.47	3626.17	2217.93	2218.66	1993.3
G1SMU8	3	1570.4	2246.94	1222.39	786.502	1458.45	609.854
G1SMY6	15	4026.58	4057.03	3673.47	3844.11	4378.47	3114.53

G1SMY7	17	15614	14119.4	18542.6	8574.93	10246.7	10071
G1SN14	28	16635.9	21019.3	13999.5	14937.6	12818.9	12867.7
G1SN51	2	4511.73	4978.6	2824.1	4517.13	5031.6	7845.47
G1SN56	1	618.198		1536.79			
G1SN60	13	3350.98	3475.79	3659.43	2686.45	2895.11	2343.86
G1SN67	2	1073.35	1273.2	680.441	752.044	795.963	584.22
G1SN68	7	1686.95	1624.02	1773.55	1475.88	861.476	1734.21
G1SN70	4	1718.26	2061.79	1834.28	713.866	1023.88	731.201
G1SN76	4	1036.1	694.13	1674.58	567.19	538.149	790.814
G1SN82	3	620.336	629.324	710.962	717.129	611.759	706.824
G1SN83	5	615.525	1147.69	668.822	1187.61	853.839	1043.21
G1SN87	9	1477.49	1985.01	2091.87	1665.93	942.563	1590.81
G1SNC7	4	5753.8	6177.73	8248.18	12282.8	14418.9	15504.1
G1SNJ7	4	543.698	796.141	451.411	1755.06	1609.96	1533.1
G1SNM1	19	5383.79	4668.67	6150.22	3157.07	2607.15	3005.62
G1SNP7	4	1226.83	1139.08	1606.72	1009.63	511.316	484.148
G1SNQ8	10	6740.39	5357.57	6191.36	7195.85	5391.1	7035.69
G1SNS5	25	9176.84	9670.63	8584.74	4865.24	4562.15	4830.57
G1SNT1	2	660.376	566.646	701.508	781.951	586.849	465.211
G1SNU5	3	4671.38	5079.93	6737.04	5319.95	4814.17	6566.18
G1SP40	8	1479.98	1408.2	1745.75	1597.61	1569.79	1779.54
G1SP46	3	623.108	700.927	645.417	1103.69	1574.09	1366.65
G1SP54	33	17953	19921.1	18623.8	13761	13111.8	13258.8
G1SP97	20	411270	413235	389274	305917	300623	318481
G1SPD1	1	372.742	1018.73	356.387	688.151	873.293	657.173
G1SPF9	148	250181	265843	258352	469564	488804	492191
G1SPH6	10	4199.6	3572.83	3177.11	1963.04	1864.19	1453.86
G1SPJ6	2	1090.91	1169.69	927.673	598.692	303.496	438.972
G1SPK4	5	1573.34	2005.45	1047.95	2012.38	2027.71	1725.82
G1SPP0	7	2277.95	1817.03	1787	1632.37	1729.09	1506.68
G1SPQ3	1			1004.96	196.563		
G1SPX0	29	5168.95	5519.78	6368.49	3830.47	3773.1	3906.6
G1SPZ7	4	6968.61	6017.8	8782.82	7311.19	6036.38	6765.68
G1SQ01	7	4028.87	3980.82	3763.23	1069.25	646.982	747.878
G1SQ02	15	31590.9	38695.3	24213.7	15787.7	14160.8	15081.7
G1SQ22	17	4229.41	3235.7	4696.63	2011.53	1676.41	1750.29
G1SQ31	9	1703.09	1813.72	1089.24	1315.91	982.181	1250.61
G1SQ46	5	2054.61	3901.85	904.149	555.772		340.387
G1SQ70	98	255625	356092	215382	286430	279022	304566
G1SQA8	5	1494.08	1237.41	1247.71	1209.15	1111.9	1010.34
G1SQB4	7	1188.34	1033.52	812.684	1261.31	1732.12	1521.31
G1SQC7	23	21063.5	15912.9	25650.2	18344.7	15241.8	17732.8
G1SQE0	1	946.543	1026.05	1565.12	2061.7	2674.37	2678.94
G1SQF0	1	1133.97	896.022	951.749	1463.38	1003.06	1300.66
G1SQF5	3	374.099	492.485	299.413	593.014	675.198	261.974
G1SQG5	6	3160.95	4661.23	1960.7	1327.51	2490.55	1026.69
G1SQG6	14	10686.1	13769.9	5538.48	9838.31	10212.7	9526.18
G1SQK3	3	503.714	1465.82	724.638	989.857	731.952	1113.38
G1SQP2	4	1596.72	1655.02	2665.9	812.853	1191.95	1003.87

G1SQR7	3	2548.65	2290.43	2912.45	1917.74	667.323	2130.86
G1SQU1	8	17695.9	18461.9	14772.9	61036.1	75103	59885.9
G1SQV5	3	1993.32	1723.59	959.537	582.572	862.667	669.789
G1SQW3	4	7521.58	6686.5	6391.46	14571.1	19184.3	12729.7
G1SQW5	1	308.638	389.32	206.47	406.473	302.352	629.507
G1SQY8	4	2812.17	3389.2	947.36	522.349		1149.13
G1SR03	61	26299.9	29054.6	28301.3	11883.9	12503.6	11889.8
G1SR15	34	7393.27	6876.75	5704.91	13424	11224.1	12713.6
G1SR28	5	4461.29	3332.19	4089.05	4658.48	3729.31	4155.44
G1SR29	24	5611.39	6370.45	5240.84	5493.96	5976.56	5936.38
G1SR33	1	562.72	307.341	1159.89		215.912	
G1SR80	2	585.248	861.762	289.125	824.493	488.512	565.497
G1SRA2	6	51704	71684.7	39803.5	68268.3	57625	79207.6
G1SRA9	1	49536.6	46499.9	58824.7	22072.2	17902.1	20833.8
G1SRB6	10	4456.13	4397.2	3880.75	3276.71	2717.14	2644.72
G1SRB7	7	2699.46	2655.2	3860.64	2495.18	2684.97	2678.69
G1SRD2	3	976.86	717.606	971.092	666.389	630.889	769.617
G1SRF7	2	1192.66	1045.89	698.216	463.071	680.613	
G1SRL1	9	2150.01	2983.89	2020.19	2141.76	1734.73	811.373
G1SRL3	2	670.882	578.051	795.778		300.556	
G1SRP8	4	3275.86	2922.17	4323.97	4558.65	2593.19	3803.82
G1SRQ2	6	3207.81	4730.62	2529.1	2935.87	2991.88	3075.11
G1SRQ7	2	2403.93	221.294	1697.82	372.198		
G1SRS3	2	690.631	665.138	1065.98	1078.32		
G1SRW5	5	2855.03	2501.9	2994.7	2254.77	1591.3	1622.56
G1SRW8	2	323.22	228.819	598.075			326.617
G1SRY0	4	1406.29	1835.46	1541.09	1312.49	1799.55	1725.86
G1SRZ0	1	1277.38	756.639	794.842	233.173	790.081	733.064
G1SS66	35	23489.8	28223	15584.5	23647.3	22778.6	23374.8
G1SS69	48	38963.1	43109	32074	15804.8	16544.7	16124.8
G1SS73	5	3537.76	2223.21	3884.16	2018.04	1579.36	1611.77
G1SS91	176	1.56E+006	1.21E+006	1.50E+006	2.00E+006	1.91E+006	1.99E+006
G1SSA2	18	1937.02	2637.74	2161.9	1257.78	1321.64	1418.43
G1SSA8	15	1748.03	1724.19	1207.77	5457	5558.09	4689.88
G1SSC9	4	1274.51	2377.38	1261.49	874.653	1177.13	1105.83
G1SSL2	2	1309.54	1165.59	2028.81	970.887		779.229
G1SSL8	1	635.979	1192.09	487.408	1169.5	686.873	456.585
G1SSN5	8	7412.3	7422.82	6339.4	3180.05	3526.66	4231.89
G1SSP5	3	114048	115609	49323.7	16268	20428.4	24096.5
G1SSQ6	30	20424.9	18111.2	16211.7	26181.9	23701.4	26253.5
G1SSS1	2	1253.53	1127.61	530.816	1696.58	2786.36	2146.85
G1SSX2	5	926.599	1228.86	1462.88	1203.86	881.34	865.577
G1SSX7	2	954.876	703.134	970.014	664.672	496.962	476.37
G1ST08	1	507.507	347.401	362.857	375.462		360.749
G1ST17	5	17551.7	13127.7	19899.8	22682.5	15586.8	18157
G1ST33	8	7304.94	7752.47	7822.91	7163.65	7001.12	7644.91
G1ST52	6	1737.01	2249.92	969.789	1456.82	1070.76	1115
G1ST54	7	1518.81	2550.33	1303.57	1973.04	1962.41	1700.24
G1ST56	3	1456.42	1405.39	1388.99	833.056	1247.24	730.774

G1ST63	1	1112.44	1309.42	1362.35	915.625	664.878	595.758
G1STA7	17	48817.4	55539.7	32778.9	26242.6	26635.4	30348.1
G1STB1	1	296.165	258.349	436.883	151.774		
G1STE4	2	2015.63	1245.16	1991.65	2580.58	1597.99	2578.85
G1STF9	10	3848.23	3303.28	3745.13	1949.97	1983.27	1748.61
G1STH4	2	1712.1	1496.5	1939.71	866.809	1211.74	1916.99
G1STJ4	34	398629	488217	380342	377991	425665	404175
G1STK3	4	6109.77	5890.7	8559.49	10934.7	9655.87	10526.6
G1STM6	8	1610.97	2945.46	1750.23	5249.69	4240.93	4754.6
G1STS3	2	433.759	918.849	764.579	814.223	874.893	425.183
G1STT3	34	51368.5	86640.4	28521.2	11610	11118.7	13722.4
G1STW9	1	1105.32	935.357	1157.06	1323.77		1011.48
G1STX3	2	1181.94	984.219	635.986	678.39		888.075
G1SU04	6	1106.59	2041.79	1109.57	1042.71	1369.58	1430.65
G1SU36	16	1.09E+006	1.03E+006	909436	1.52E+006	1.27E+006	1.51E+006
G1SU42	2		606.032		429.413	902.236	867.684
G1SUC8	17	2248.96	2944.47	2270.92	1440.29	1631.47	1561.37
G1SUH8	14	7555.94	7706.32	4325.95	9099.27	13065.6	27424.2
G1SUJ2	1	486.644	737.767	549.874	1125.12	1431.6	734.286
G1SUT0	1	467.543	519.11				
G1SUT8	1					266.443	
G1SUU7	2	998.306	640.387	1370.38	752.404	492.009	580.907
G1SUW0	10	4032.79	4102.47	3360.91	7415.91	6135.85	5878.04
G1SUW1	20	14257.3	20841.8	11991.1	6462.55	7116.6	6480.94
G1SUX1	17	66810.1	96491.5	60002.5	101812	103970	116185
G1SUY5	3	1729.65	1583.87	1221.7	1959.08	1571.29	2098.77
G1SVA3	15	3526.11	2981.57	3740	2135.92	1993	1923.41
G1SVE4	9	2793.06	3160.84	2845.56	3219.45	3046.32	3172.08
G1SVF9	2	691.068	551.958	573.74	654.541		
G1SVH0	22	5154.19	6019.58	5858.77	8799.85	8598.19	9369.19
G1SVK5	3	5492.8	3750.12	4470.13	6222.07	6031.7	6980.3
G1SVP7	11	4000.54	6962.31	2637.06	3281.34	2514.63	2785.28
G1SVT4	8	3760.36	3412.54	3749.65	2240.19	2114.96	1817
G1SVT9	1	1284.27	932.025	951.343	760.512	606.563	961.385
G1SW24	16	3251.43	3147.51	3957.75	2254.2	1985.91	2095.14
G1SW47	2	1229.6	1172.67	1420.3	1163.93	677.98	1113.94
G1SWD8	3	643.646	845.427	540.532		681.815	489.254
G1SWF0	40	553054	588682	421386	238981	243210	255909
G1SWF7	5	710.195	977.798	712.367	1304.78	1216.27	1056.7
G1SWG7	43	7465.29	6640.79	6604.14	13849.2	13203.7	13535.7
G1SWI7	6	71274.5	48224.9	73410.4	120891	101388	123019
G1SWK8	9	23116.2	23071.7	22149.5	46835.6	48135.6	45401.1
G1SWR0	7	2130.31	2230.97	2387.23	2595.33	2181.06	2083.76
G1SWS6	2	2755.32	2228.91	2318.84	2919.66	2160.73	2622.27
G1SWS9	29	5000.16	4828.98	4608.93	11090.1	10705.2	11031.9
G1SWV6	17	5225.86	4104.97	4598.53	8497.04	8493.85	8007.6
G1SWX2	3	1490.31	1150.86	890.59	789.916	916.582	912.339
G1SWZ1	4	1448.42	1356.67	1510.21	1241.77	1010.98	1043.63
G1SX17	22	14626	18427.9	9829.77	8794.93	9565.58	9626.03

G1SX80	19	2140.06	1740.51	2135.28	3124.26	2608.79	2799.69
G1SXG6	3	2613.8	3230.81	2645.81	2014.85	1784.42	2001.73
G1SXG7	1	315.621	521.385	353.433	226.027		242.242
G1SXL3	14	5517.17	6215.22	5066.8	3007.46	2392.6	2647.86
G1SXM6	2	2945.02	1170.47	3805.63	1747.76	1000.83	1747.42
G1SXQ0	14	2716.97	4174.81	2670.22	2818.31	2534.08	3092.58
G1SXR1	4	970.565	1105.36	1286.66	1389.66	1018.24	1209.74
G1SXT1	2	820.843	732.929	735.696	320.465	595.521	560.762
G1SXZ7	7	1785.59	1490.3	1825.99	1695.1	1685.34	1933.4
G1SY36	48	142416	210979	121969	111534	118533	125998
G1SY93	8	39363.1	30497.3	35024.2	19368.6	17896	20736.2
G1SYB4	12	14245	13794.7	15427.7	8849.46	9060.06	10034.4
G1SYC9	12	17366.7	16569	13121.3	10065	10782.8	10976.6
G1SYD3	2		1248.64			630.865	354.643
G1SYI2	13	13378.6	14316.3	12308.4	8907.61	7786.93	8796.53
G1SYK4	124	479754	467112	461615	377664	343632	396234
G1SYL3	4	1462.71	1644.54	1300.69	970.329	1173.59	787.651
G1SYL8	2	794.435	1735.84	642.555	982.994	973.051	878.116
G1SYM2	18	3507.92	2825.17	3393.84	2452.69	2235.86	2538.05
G1SYM7	2	567.24	374.394	427.545			
G1SYQ5	10	2682.21	2262.31	2970.56	2189.15	2127.46	1892.99
G1SYS4	1		500.628				266.574
G1SYU0	2	529.615	517.68	578.07			771.911
G1SYV0	14	2902.09	1968.28	2825.33	1413.83	1273.54	1224.94
G1SYV9	176	161712	180494	140511	110332	103230	106169
G1SZ03	16	4694.81	4491.71	3481.33	1921.58	1840.65	1687.96
G1SZ14	26	38667.7	34233	41105.5	83595	80912.2	81643.4
G1SZ19	1	2251.51	1569.64	1962.97	1429.28		
G1SZ34	4	1412.8	1044.58	1243.98	863.087	1025.2	705.766
G1SZ90	8	28510.3	19187.9	28299.3	523.54	228.927	487.025
G1SZ91	2	676.336	435.714	523.066		852.867	450.991
G1SZA3	1	252.241	161.811	147.61			179.043
G1SZD6	15	28669.9	36357.8	33081.3	22649.4	22894.2	23690.4
G1SZE0	13	4401.86	3882.52	4185.06	2589.55	2814.54	2732.2
G1SZF9	4	1363.82	565.674	453.373	918.977	734.861	730.803
G1SZJ5	1	284.744	329.507	747.271			118.923
G1SZL8	4	1935.69	1977.99	2248.52	1375.22	1009.99	1798
G1SZN9	21	5264.26	4450.19	5320.67	3196.7	2457.11	2707.07
G1SZP0	1		832.712			721.956	
G1SZP6	9	5005.94	4224.29	3876.06	6115.88	5899.74	6460.73
G1SZQ2	4	1598.35	1257.26	1844.57	1529.51	869.635	1269.42
G1SZT8	1	369.657	893.677	644.425	834.476		331.304
G1SZW0	22	4674.12	4239.76	4260.11	3241.68	2956.78	3142.82
G1T013	8	5084.36	9878.28	3415.84	6001.34	5740.47	4041.88
G1T035	10	2410.71	2434.11	2502.99	1701.79	1323.95	1659.48
G1T039	1	434.045	261.033	234.295			
G1T044	4	2008.25	1455.07	1219.94	1638.29	1733.2	1361.44
G1T086	5	2986.86	3298.05	4183.93	8071.07	5440.72	5886.9
G1T088	8	563.295	470.655	353.134	1311.26	1276.88	1523.41

G1T090	9	1357.22	1542.38	1046.03	636.474	1075.21	806.174
G1T0E9	2	832.071	1312.17	849.006	967.855		1498.37
G1T0F8	33	231970	233185	290405	197897	223445	248867
G1T0G5	10	3872.8	2864.55	3585.25	4614.49	4200.2	5125.41
G1T0H7	3	1763.42	1437.76	1767.58	1228.54	1099.94	1455.79
G1T0J6	51	54630.6	61002.5	44642.1	81026.3	75597.5	82168.9
G1T0K7	3	829.138	767.369	643.018	2195.81	1755.88	1617.06
G1T0M0	1	1563.46	1205.53	1584.49	856.797	1181.64	722.838
G1T0N5	3	1532.1	1893.9	1629.6	774.161	1284.65	1754.1
G1T0P2	9	3886.15	3160.29	4991.24	2150.65	2281.88	2153.48
G1T0Q0	19	238588	241790	289791	420300	373812	438216
G1T0R8	1	6140.19	5348.81	6157.11	7569.8	7416.86	6918.3
G1T0R9	9	9690.84	15232.5	3838.83	3655.33	2807.77	2798.47
G1T0T5	3	1379.44	929.985	1211.7	1071.23	1009.91	630.965
G1T0U2	15	4317.22	4168.1	4673.05	3299.73	3784.94	3373.27
G1T0U8	35	89362.4	106739	103809	121917	120769	137700
G1T0X2	46	56936.8	57417.9	51945.7	116881	122393	125156
G1T0Z8	8	2004.8	1900.83	1928.58	1479.45	1820.65	1164.9
G1T107	7	3486.09	6727.57	1489.59	2234.24	2510.85	2059.26
G1T127	39	28741.7	25461.5	24269.4	24611.1	22787.8	24476.1
G1T138	8	4533.9	5505.69	3041.32	2490.91	2583.43	2066.62
G1T156	13	5883.17	7631.46	4674.59	9219.09	8216.49	7800.33
G1T157	13	4001.65	7897.61	2787.64	3560.23	3085.4	3143.41
G1T196	4	790.662	387.073	717.049	422.545		223.248
G1T1B8	30	60328	79428.2	79504.2	152085	134006	166714
G1T1D9	6	1720.11	1655.34	1409.48	1176.64	935.183	940.271
G1T1G4	10	7825.39	7005.17	8700.34	5493.07	3832.06	5764.35
G1T1G5	4	5237.5	4103.22	6099.9	6448.96	3518.41	4909.65
G1T1U7	25	4320.85	5096.69	4264.75	3506.08	3355.62	3088.65
G1T1V5	5	2311.35	1918.49	1999.82			
G1T1V9	20	21347.8	19838.7	23595.2	16833.4	15547.6	16566.1
G1T1Y7	8	7578.84	8662.75	5227.42	11414.9	17116.1	29150
G1T1Z5	27	70381	68840.4	79611.9	118198	116651	127302
G1T221	10	8535.69	6591.79	8614.93	1785.11	1417.63	1910.35
G1T235	4	19795.8	21568.4	19468.5	30966.1	49897.6	33756.8
G1T277	11	11077.3	16625.6	6300.78	10258	11017	9975.69
G1T2I4	20	2680.68	2645.44	2418.35	1376.35	1271.75	1270.14
G1T2J0	2	551.578	502.95	405.109	958.597	913.801	1484.5
G1T2L1	17	64620.8	53379.8	68095	130600	123076	135614
G1T2Q8	3	2504.53	2275.83	2402.56	2264.53	2208.18	2214.07
G1T2V2	21	13573.5	15746.3	9303.34	1505.48	1922.51	1385.57
G1T336	6	2069.64	1948.08	1944.15	2600.74	2059.35	3413.26
G1T346	20	3504.69	4639.18	3068.89	4015.45	4255.35	4093.65
G1T373	14	6957.95	5408.3	8431.33	4714.46	4404.92	3991.58
G1T398	6	2140.2	2610.42	1709.29	2325.3	2134.9	1656.59
G1T3D7	6	41785.9	41116.8	38732.4	49766.6	56123	42557.7
G1T3E6	10	25281.3	19031	24382.5	16680.7	13633.8	17800.8
G1T3P1	6	2429.8	2586.46	3006	1527.27	1186.77	1805.58
G1T3Q2	3	610.025	768.19	758.775	415.758	335.307	127.846

G1T3S1	11	9390	7806.2	11657.2	4646.58	4110.13	5295
G1T3V2	2	1833.56	1547.57	2720.5	2967.92	1787.2	2534.01
G1T3Y8	12	2405.92	2226.75	3097.75	1846.44	1889.56	2266.11
G1T3Z1	17	141712	105754	140915	198501	182504	184482
G1T3Z6	21	9988.02	8403.51	10357.9	5268.25	4468.04	5370.04
G1T418	6	1494	1872.37	1667.29	2435.73	2906.88	2076.04
G1T419	2	49421.5	69144.5	18475	52909.7	48255.9	33659.8
G1T432	35	32611.6	37039.2	23199.4	15469.4	18436	15922.5
G1T437	2	1675.97	1365.59	1755.02	1819.14	1057.72	1553.95
G1T464	29	48387.9	50773.9	45692.8	24301.5	23261.7	25251.2
G1T466	5	915.778	1099.04	948.287	823.946	646.368	939.023
G1T4H0	6	2929.69	2575.13	2901.86	1929.23	1754.23	2274.01
G1T4H3	12	2297.21	1937.3	1749.59	3003.31	2643.71	2577.55
G1T4I7	2	500.635	415.94	630.034	554.481	497.008	419.557
G1T4J9	7	12945.1	6230.98	17822.8	11165.3	13084.1	9276.17
G1T4N8	8	4083.52	2769.62	4199.14	2232.09	2176.25	2270.8
G1T4P7	11	9193.47	7061	8758.53	3906.34	3472.93	3712.05
G1T4Q8	2	9007.95	9643.62	6124.85	11878.7	14989.6	9443.24
G1T4Q9	19	24624.5	20838.6	27652.6	31126.2	28944.6	29809.9
G1T4R0	1	567.331	836.343		865.549	989.565	671.609
G1T4T6	87	36060.8	41215.1	22998.4	3945.67	3938.11	3837.47
G1T4W2	2	1280.18	1789.19	958.179	1439.2	1742.68	1768.02
G1T4X8	20	24644.1	24925	27794.8	62245.6	54009.7	59045.7
G1T4Z2	36	4815.27	4542.46	5277.35	3435.9	3009.44	3253.47
G1T507	1		2519.84	746.51	5269.56	7242.27	5936.12
G1T519	18	68152.1	68946.9	58100.4	166028	161270	166258
G1T543	6	682.534	619.971	739.841	1421.34	698.802	1212.3
G1T550	7	7346.2	6753.42	7489.57	6043.32	5582.56	5744.44
G1T567	12	23466.4	24711.8	24303.3	19352.5	15758.3	18410.1
G1T568	4	1595.39	1574.76	2262.84	1194.02	970.732	1415.42
G1T5A0	2	836.716	786.839	869.523	493.358		394.807
G1T5C3	4	3998.11	3923.96	4305.26	5665.93	4804.25	6528.48
G1T5H0	10	5501.98	6019.01	5801.33	8166.97	7067.39	7725.11
G1T5K0	4	1199.06	985.462	733.547	1004.57	766.164	836.121
G1T5K3	9	96189.2	79064.4	145961	135463	96075.1	121950
G1T5T0	5	927.511	977.609	836.521	1770.08	1940.42	1655.15
G1T5T8	3	1491.74	1491.43	1326.45		535.669	789.08
G1T5W5	2	508.163	665.19	418.448	654.998	537.624	615.668
G1T5Y1	5	1506.08	1494.37	1732.74	1000.54	1085.92	843.729
G1T5Y2	6	13472.5	13429.8	8819.75	9876.7	8353.16	11762.7
G1T5Y9	19	8722.63	9223.28	8224.5	11614.4	9711.02	11301.8
G1T5Z7	2	673.007	419.588	781.8	227.294	514.818	257.706
G1T616	1	276.178	509.027		406.162	362.111	373.872
G1T641	1	459.841	289.649	350.782	264.995		197.786
G1T643	15	1578.76	1789.32	1551.78	1155.59	1102.06	1040.9
G1T647	5	1534.31	1791.7	1434.11	1476.59	1664.26	1544.11
G1T661	11	7215.36	6374.84	6385.71	8534.53	6565.62	7796.05
G1T670	13	65649.4	54582.1	66672.7	117309	123843	126219
G1T671	8	3005.44	3625.92	3234.04	3982.68	5631.18	5478.38

G1T6B3	10	1421.86	1148.96	1578.19	951.67	838.851	773.489
G1T6C0	3	2557.92	1899.51	2192.79	1676.67	1780.37	2320.18
G1T6D4	13	2341.81	2266.09	2192.71	1689.25	1344.58	1588.76
G1T6F6	2	453.168	1024.89	363.638		401.614	412.903
G1T6K0	7	27800.4	35868.2	24199	54156	44846.9	45648.8
G1T6Q0	5	1007.95	1042.02	781.695	2171.7	1929.48	1490.33
G1T6S6	1	6900.06	2744.56	5205.81	3366.03	4716.87	4947.47
G1T6W7	27	5496.67	5155.03	6000.85	6083.19	5498.68	6127.39
G1T702	1	379.925	407.902	616.681	577.248	725.258	946.362
G1T704	20	12714	14080.7	12326.7	29260.5	27662	30847.5
G1T705	3	884.415	1043.55	1325.01	1329.92	1299.67	1586.91
G1T739	6	17883.7	14586.7	16511.3	34141.8	31029.4	32572.6
G1T780	1		165.085	232.287	399.372	374.656	257.515
G1T7A8	4	6624.45	6891.26	7045.4	9637.7	9477.45	11241.8
G1T7B1	1	1368.77	982.587	969.77	758		563.312
G1T7C7	2	557.169	716.361	396.716			
G1T7D9	1	815.039	598.813	865.603	747.663	496.465	273.294
G1T7F1	4	6739.65	5447.71	4724.76	7725.97	4951.78	6199.92
G1T7I4	28	3692.38	3675.64	3832.06	2491.55	2355.64	2263.83
G1T7I8	6	1854.75	1761.91	2027.57	1734.72	1972.2	1657.52
G1T7L5	1	738.664	1206.54	784.551	502.039		821.39
G1T7Q5	3	1637.9	954.84	930.008	1239.43	925.555	1137.53
G1T7R2	21	7812.26	10770.4	3875.98	7486.26	7523.4	7819.34
G1T7S0	4	54318.2	44287.1	63363.1	17480.4	36111.4	18405
G1T7S1	2	1177.35	1141.24	963.885	1145.95	678.257	701.972
G1T7U6	25	77681.8	81609.1	83877.3	112748	129913	125881
G1T7W2	7	1233.26	1324.83	1877.78	911.201	941.027	1038.88
G1T7X6	15	4336.38	3379.57	5184.98	9192.08	9094.3	7760.27
G1T7Y7	3	1448.41	1204.47	1488.73	944.488	963.158	829.559
G1T7Z0	19	5815.94	5861.21	5314.55	6109.39	5404.4	5995.42
G1T7Z6	43	28598.8	26038	33889.9	15867.1	11706.3	16617
G1T804	7	35592.6	31783.7	29044.8	38621.3	46920.4	38279.4
G1T809	18	176084	204855	124458	215185	222197	193339
G1T821	14	14136	16875	14588.9	25789.7	24196.1	25258.5
G1T823	2	1229.1	1455.91	1042.23	2551.64	1642.96	2804.26
G1T874	1		194.84	200.083	112.898		104.623
G1T892	3	648.671	697.931	483.45			158.334
G1T8H3	3	1387.24	1490.34	1327.76	878.569	509.381	685.994
G1T8K0	1		133.449	1067.73			
G1T8P4	1	6208.14	4506	5598.79	6132.58	5200.32	7017.91
G1T8R1	7	1736.48	1507.69	1768.4	1367.77	1133.75	814.133
G1T8T3	7	126535	93505.6	127627	101140	105303	102606
G1T8T7	5	1507.95	1669.19	1408.02	3051.27	2899.29	3014.88
G1T8V2	12	150563	148205	199861	226399	233586	235541
G1T8X8	12	5530.12	5181.4	6827.51	7180.41	6807.61	7710.94
G1T8Z0	24	103844	134004	83462.5	66495.8	62615.3	62219.7
G1T918	10	48534.3	38366.6	44967.6	83179.5	88099.3	84848.3
G1T970	2	732.285	666.456	787.705	501.516		370.53
G1T9D6	6	5865.5	6399.13	4075.64	3423.74	3519.95	2980.62

G1T9F3	18	10008	10916.2	9531.81	5666.2	5345.72	6455.39
G1T9H4	6	4279.12	4800.19	3243.12	1444.89	1806.12	2065.64
G1T9I5	1	275.732		405.194	612.082	262.456	256.277
G1T9L2	1	342.583	105.599	447.835			
G1T9L4	5	7850.66	6545.25	10503.4	9865.67	9256.61	8350.73
G1T9M9	21	37485.9	44808.1	36610.5	26854.5	28288.9	28378.9
G1T9U7	1	9433.71	9118.36	10674.3	2519.1	1029.78	1630.53
G1T9U8	1	1165.79	726.323	1835.14	611.847		1287.73
G1T9V6	8	5585.11	4831.72	6834.07	3849.38	4347.98	3013.11
G1T9W9	5	1281.53	1582.69	777.313	1937.3	2347.45	1403.24
G1TA11	18	2600.88	2150.71	2295.99	1331.45	789.068	1301.08
G1TA37	3	2126.54	1040.52	2578.94	2853	2573.98	2041.4
G1TA40	20	17390.9	17076.4	17190.3	26075	22381.8	24790.6
G1TA48	14	9609.88	6471.77	9781.09	5429.78	4906.2	6449.19
G1TA82	2	375.931	616.844	366.799	454.053	738.875	
G1TAB2	3	2652.69	1481.75	3580.23	1639.9	1767.41	1650.07
G1TAB8	2		732.759		663.61	467.418	675.408
G1TAC4	3	1954.6	2407.37	1813.22	2152.62	1403.09	1638.16
G1TAE5	2	1081.54	744.358	1288.09	420.566		378.819
G1TAF8	13	4453.37	5431.74	4294.53	3637.39	3665.87	3754.86
G1TAH7	14	3187.06	2612.05	3344.25	2739.71	2269.72	2670.1
G1TAJ7	6	1702.2	1330.83	1633.71	1407.86	1730.08	864.856
G1TAK5	3	804.497	617.751	883.947	438.223	507.591	965.673
G1TAR4	21	15277.8	13357.7	15494.2	30191	31612.9	29999.3
G1TAR5	1		146.449	366.858	1154.26	846.876	782.943
G1TAR9	5	2860	3289.52	4504.7	3707.21	2514.63	2924.36
G1TAV9	12	9028.46	8841.87	5848.85	2554.69	2109.15	3149.49
G1TAY2	1	223.609	124.96	468.799			
G1TB08	1			387.142			
G1TB18	22	13358	15323.8	11639.1	11031.7	11924.4	10495.4
G1TB61	1	922.539	597.801		1346.09	1058.41	932.195
G1TB93	13	3034.14	2835.49	2986.3	4169.67	3358.45	3572.03
G1TB95	35	16603	16262.4	16550.4	13694.3	13406.9	12941.9
G1TB99	2	1203.07	1507.45	1442.87	1085.98	1212.72	1084.12
G1TBC1	39	19609.9	18359.4	18821.4	27844.1	26540.1	29755
G1TBC8	2	1140.74	950.9	1309.73	999.07	475.104	740.526
G1TBK0	2	595.59	687.076	752.882		275.885	276.094
G1TBQ3	1	2407.37	2053.86	2816.74	1225.08	606.905	1079.52
G1TBS1	5	2369.09	2391.73	1902.25	820.573	963.52	956.507
G1TBS8	68	604485	344991	736475	296043	273337	307917
G1TBT8	1	1129.21	1543.97	1076.56	1129.26	1081.73	474.154
G1TBX7	9	10642.7	11833.7	9805.97	8821.73	6782.38	8678.19
G1TBZ2	2	372.096	268.555	557.092	301.829		416.026
G1TC07	6	1865.72	2358.94	1621.7	1499.18	1394	1265.28
G1TC10	3	2110.74	2663.38	2008.05	1943.72	2114.43	2166.4
G1TC17	1	3413.61	3136.37	3285.5	3583.57	3442.74	4309.8
G1TC73	1	1397.85	1064.56	1179.71	2170.33	2258.22	2072.54
G1TCG2	8	4115.83	4117.99	4249.99	2702.69	2811.59	2541.81
G1TCG8	1	1230.66	1404.57	1615.07	2558.6	1921.47	2479.87

G1TCI1	8	2971.89	2645.64	2523.53	5279.69	4848.27	4424.68
G1TCK9	15	4507.92	4173.64	3334.74	2559.16	2852.67	2358.8
G1TCN8	11	2055.65	1822.96	2805.05	3413.44	3403.91	3553.01
G1TCS8	17	25396.6	25542.4	25624.8	20427.3	16688.1	21867.2
G1TCW1	29	8011.96	9248.08	7845.13	14862.4	12334.2	13486
G1TCW5	11	1847.85	2239.6	1810.31	3034.14	3366.16	3377.01
G1TCZ8	17	6502.42	7369.13	8168.95	4821.3	3594.02	4537.19
G1TD16	14	3313.46	2795.46	3521.14	2497.49	1896.95	1956.15
G1TD24	1	1555.65	5540.75	1401.78	3926.54	9484.09	5331.71
G1TD94	5	3556.91	3130.17	3157.56	7525.5	6240.82	6651.06
G1TDB6	16	5690.57	4294.28	6520.76	3430.57	2229.08	2573.77
G1TDD4	2	697.459	662.829		409.17	536.576	621.066
G1TDE8	4	711.887	952.339	1108.13	906.683	272.343	1227.26
G1TDG7	8	1317.27	1381.67	979.386	644.207	990.509	1074.64
G1TDI0	9	5691.23	6383.12	5462.41	4868.87	3209.86	4741.04
G1TDJ3	2	1084.5	1092.85	1268.39	742.014	589.421	947.397
G1TDM3	7	3421.19	4066.3	1966.27	8337.55	6525.43	5813.1
G1TDN4	10	3150.6	2609.6	3249.98	3156.18	2875.5	2983.12
G1TDN6	10	30611.7	34752.2	20412.1	41020.7	58776.8	106081
G1TDT6	3	1345.92	1824.04	874.367	704.998	619.471	523.443
G1TDU5	2	635.482	307.1	536.293	1351.16	1018.83	1468.96
G1TDU9	8	3962.33	4101.22	3845.97	3128.24	2500.6	3435.67
G1TDZ1	3	3156.46	3651.14	3518.59	2347.16	1728.69	1961.26
G1TE13	5	1719.7	1336.67	1997.81	1131.12	1012.45	887.874
G1TE37	3	1432.54	1754.92	1274.1	2056.07	2464.34	1842.29
G1TE41	4	1313.94	1618.29	1889.17	1032.17	1143.98	1106.72
G1TE61	4	2556.74	2601.76	3573.81	1479.62	1376.76	1522.65
G1TE68	1	675.896	503.64	787.991	513.796	470.066	
G1TE88	12	4560.55	5234.35	4375.24	2709.75	2359.17	2319.1
G1TE96	1	444.175	374.397	223.751	225.49		
G1TEA3	13	6570.98	9842.55	8565.99	5356.95	5763.23	6025.84
G1TEA5	11	2831.43	3456.88	4212.95	3911.5	3767.94	4113.34
G1TEC1	1	25863.3	14409.5	24755.4	21429.3	17891	19574.5
G1TEG4	3	1127.4	778.324	746.895	939.772	509.89	1034.46
G1TEG8	22	3638.69	3602.69	3435.74	3901.15	3288.75	3072.35
G1TEJ7	4	2075.71	1806.47	1362.33	995.161	1277.23	1340.26
G1TEM1	7	5004.89	3914.64	2981.22			
G1TES6	4	1514.72	1642.46	1932.92	1778.43	1089.96	1946.34
G1TET0	21	24038.7	23241.4	22155.9	31751	27048.1	29376.4
G1TET2	42	58753.8	115315	25662	63639.1	65067.5	66432.2
G1TEW3	4	2524.96	2277.15	2478.82	1901.21	1817.02	1619.33
G1TF09	5	1650.81	1877.17	1691.68	2510.2	2575.53	3229.32
G1TF20	5	1306.26	1663.82	955.157	974.794	640.106	640.123
G1TF82	2	1640.49	1941.76	1700.09	1545.76	1194.88	1397.28
G1TFB5	8	3106.44	2716.37	2700.75	2247.91	2081.49	2022.81
G1TFL3	7	5217.43	4741.07	6435.66	4238.36	3025.96	4264.95
G1TFP2	2	1213.46	1040.07	452.665	454.895	1195.95	1147.17
G1TFU9	33	637130	525147	571230	697724	698720	705797
G1TFW8	55	36483.4	41169.3	39701.2	59229.5	59882.4	52782.3

G1TFX2	9	205320	66033.5	181600	134223	62674.7	75152.3
G1TFZ6	9	6730.07	6912.29	6237.21	3453.74	3423.78	3181.28
G1TG13	7	2544.98	6628.75	3547.96	3341.78	3532.99	3418.57
G1TGF1	2	3923.32	4221.08	2958	2129.22	1916.43	2198.28
G1TGM2	16	3.45E+006	2.59E+006	3.23E+006	651317	465853	584544
G1TGP6	5	2991.33	3035.93	4984.74	6569.73	5390.48	5550.95
G1TGQ1	1	822.62	758.111	834.967	845.927	441.826	543.369
G1TGV8	2	2279.78	3088.2	3005.62	2892.18	2300.16	3651.95
G1THG2	1	26982.7	18774.4	27678.1	33722.9	26397.7	42280.1
G1THQ5	18	8707.37	6673.61	7050.13	4311.54	4357.44	3400.17
G1THY3	6	3067.37	4378.99	2923.72	2148.58	2573.22	1954.22
G1THZ6	22	963627	654782	900255	1.36E+006	1.51E+006	1.14E+006
G1TI79	1	663.137	381.087	279.947			443.438
G1TIB3	9	2675.05	2398.26	2118.43	1193.48	1316.57	1495.33
G1TIR7	3	547.702	779.309	448.029	665.724	802.37	816.522
G1TIT9	9	4386.04	6226.58	4433.94	4993.26	4472.73	4640.04
G1TIU0	1	901.775	884.737	849.681	621.083	1186.02	1010.78
G1TIZ2	22	3350.17	3504.53	3770.61	4794.48	4329.64	4340.33
G1TJ17	1	761.256	994.91	367.049		415.141	327.808
G1TJ93	3	548.815	533.698	604.194	742.398	569.686	916.878
G1TJC3	1	2527.18	762.587	3381.48	862.517		497.547
G1TJG3	4	808.844	915.01	1159.29	1630.11	1847.3	2149.75
G1TJL6	1	454.561		3075.8			
G1TJP4	16	5450.16	5155.84	5252.66	7295.84	6303.61	6487.21
G1TJR8	2	10903.3	7542.83	10629	12463.6	9432.27	14323.6
G1TK26	8	3396.11	3614.71	3736.06	2938.55	3345.86	2936.11
G1TK32	6	8163.48	9003.02	8717.9	6050.2	4732.65	7161.48
G1TK85	11	393646	341080	492712	527428	418045	545818
G1TKC3	1	3085.69	2149.89	3493.34	1753.53	656.938	1822.96
G1TKE3	10	28350.9	39299.6	14948.5	16257.8	14631.9	16796.7
G1TKE4	13	15059.4	18821.2	12848.9	22092.3	21137.9	20810.9
G1TKL2	13	98991.5	100146	112333	48199.4	44145.8	49592.4
G1TKL6	3	1149.34	1017.2	1528.77	1208.73	856.26	1065.73
G1TKM0	1	167.9	1088.77	295.001	1139.73	2271.47	1404.13
G1TKQ8	2		586.426	404.688			
G1TKY3	33	6769.69	7838.97	7217.42	8091.77	8901.04	8367.62
G1TL57	1			5698.75			4243.63
G1TLD3	15	1816.99	1569.36	1305.71	4385.41	3967.12	4075.3
G1TLE4	4	2565.91	2794.12	2516.26	1916.14	1701.79	2358.61
G1TLH1	5	2114.01	2024.28	2127.38	1189.14	1338.19	1385.2
G1TLS4	3	538.731	544.371	382.313	819.576	670.621	1049.71
G1TLT8	7	2532.59	2581.24	2454.38	1767.75	1846.32	1916.71
G1TM00	4	2025.5	3209.94	3820.74	2375.1	2210.81	2672.87
G1TM29	5	1374.02	949.308	1264.77	1708.53	1341.29	1649.63
G1TM35	1	378.459	1285.21	180.323	675.917	691.524	713.927
G1TM88	25	126949	186167	72704.6	44090.7	41793.6	47575.8
G1TMB1	15	164971	177580	172253	234982	231336	277187
G1TMB9	1				20058.8		
G1TMC5	5	2138.85	2418.52	1703.71	2104.82	1661.99	2516.3

G1TME7	1	809.877	1066	386.083	744.211	1300.49	824.213
G1TMP1	1	23840.7	12038.2	12497.2	19249.1	19142.4	63268.9
G1TMS5	33	12063.1	12042.6	12767.9	5722.76	5690.74	5462.78
G1TMV1	15	35500.6	31191.8	25254	25572.4	24445.8	26873.6
G1TN08	9	2549.08	4894.81	1795.98	2845.04	4988.25	3292.22
G1TN25	19	36102.8	65675.8	13976.9	10927.5	10148.9	11548.7
G1TN86	3	9067.61	7115.45	8831.69	16690.5	14839.8	16241.6
G1TN89	25	4387.15	3943.22	3690.02	8741.81	8295.68	8026.65
G1TNI4	16	8046.85	9812.04	5944.44	2191.41	1850.54	1987.16
G1TNJ2	4	2337.85	1640.07	3060.01	3200.95	1315.77	2423.16
G1TNS3	2	1481.44	2405.32	1087.8	4405.41	2001.05	2730.87
G1TNT5	1	576.046		660.391	662.393		606.404
G1TP15	18	1674.99	1997.5	1739.2	1153.05	1247.08	1192.52
G1TP36	3	4617.53	3911.87	5030.64	4279.66	6150.66	5088.31
G1TP56	2	1495.21	1524.03	2496.1	1193.73	1624.07	1036.73
G1TP57	18	4930.12	5493	4396.08	3391.24	4074.26	3501.02
G1TP66	2	1488.32	703.123		859.376	554.582	335.955
G1TP67	2	10077	7745.51	8050.91	8791.6	9551.19	8231.89
G1TPR4	1	3183.41	2511.23		3181.78	4266.64	3254.2
G1TPW2	5	2020.47	3201.95	2122.11	4433.58	6194.67	5146.4
G1TPZ1	9	8822.08	12268.6	7298.88	14688.9	14895.6	14414.7
G1TQC4	5	22506.9	33495.1	19640.4	24764.1	26614.5	20809.8
G1TQC5	5	488130	51626.5	508452	82798.8	4536.86	72755.1
G1TQJ4	5	1806.27	1306.23	1988.35	892.577	1189.36	1320.49
G1TQW5	1	1138.95	1583.07	1023.49	1105.77	1569.89	912.478
G1TR82	31	205299	218224	235291	102103	94449.1	109577
G1TRA4	6	7374.05	5894.21	7391.69	3886.04	3549.7	3648.14
G1TRC0	13	4221.33	4273.24	4859.36	3045.15	3492.48	3034.58
G1TRF0	1	1759.06	4967.6	1637.83	2553.98	4773.66	4176.15
G1TRG8	11	5029.06	5482.19	4427.33	4779.11	3970.61	4273
G1TRH5	17	9683.96	10902.9	4655.43	1810.72	2041.01	1636.3
G1TRR7	2	672.187	1227.33			791.001	1295.49
G1TRR9	2	414.026	416.454	274.248	2599.66	1517.97	1757.21
G1TRS4	4	2732.84	2355.57	1959.25	4423.13	3361.06	4582.34
G1TRW8	20	82005	63691.1	102789	147597	173706	170441
G1TS93	9	2107.5	2109.85	2067.19	1803.12	1408.88	1724.44
G1TSJ0	3	62526.8	50308.8	57116.5	74561.1	57113.9	94841.5
G1TSK5	1	1064.77	2048.24	1027.8	2057.58	1189.42	1625.27
G1TSN0	6	3824.6	3351.06	3578.7	9093.09	11261.3	8877.73
G1TSP2	2	526.127	792.201	558.045			
G1TSY8	27	121026	140645	88556.6	142248	133308	145413
G1TT53	2	2589.65	9432.17	2202.61	7626.21	16772.7	8253.31
G1TT67	11	2460.26	2538.88	2752.16	2373.95	2003.77	2698.33
G1TT75	1	919.481	767.583	433.386	1033.04	1385.82	1372.75
G1TT99	5	6635.43	5944.34	6545.83	9746.08	9796.57	11052.1
G1TTH9	2	167.448	417.523	336.126	519.556	525.412	424.82
G1TTI2	2	21906.5	65570.4	19745.7	98505	87513.2	69285.9
G1TTJ1	16	19787	15867.6	19519.8	17131.8	15825.8	17620.2
G1TTJ3	13	2514.3	2801.25	2245.41	5849.08	5007.39	5395.44

G1TTX7	2	1148.74	1134.05	754.433		218.64	
G1TTY5	2	1231.39	1145.71	792.276	1192.86	1250.98	1821.66
G1TTZ8	1				232.115	282.853	287.237
G1TU01	1	216.25	502.44	453.062	620.769	382.406	191.198
G1TU32	3	3597.49	3542.95	3180.64	1792.66	1373.39	2158.76
G1TUA3	2	2237.17	2356.09	1903.11	1289.51	911.743	1007.18
G1TUC3	2	10972.5	9623.67	11309.6	16681.4	12564.9	20541.1
G1TUG7	2	2193.29	1782.26	1939.47	1865.13	1387.01	1707.82
G1TUK8	1			1511.35	2280.97	2987.37	1815.3
G1TUP1	2	1689.14	825.172		1594.26	1198.14	1286.67
G1TUQ2	1	1294.97	1727.89	1464.19	2368.06	3458.76	3204.38
G1TUU8	4	2218.72	1923.2	2727.3	3419.55	2843.57	2661.74
G1TUX2	2	1394.81	967.252	1238.29	483.325	497.98	787.282
G1TV17	7	15619.2	20456.8	14327.9	29454.4	29661.7	25816.5
G1TV43	2	585.524	820.741	710.512	974.261	511.228	649.6
G1TV79	19	110473	96017	107966	134046	123294	140414
G1TVH4	4	980.609	1127.65	603.885	3059.25	2775.17	2606.64
G1TVH9	10	14802.2	16814.1	13998.7	9904.43	7804.19	7035.61
G1TVJ8	3	488.169	251.936	353.793	881.367	633.412	851.689
G1TVS4	2	20896.6	39807.1	10357.9	18444.8	21325.7	24956.2
G1TVU4	8	2014.42	1810.73	1853.48	2132.66	1768.43	2167.66
G1TVU6	9	60625.2	41327.3	54610.1	158201	138288	147174
G1TW85	1	50261.1	36107	50361.4	58054.9	51728	74292.8
G1TWB8	1	5133.23	3616.08	4502.34	5700.56	5590.13	5959.88
G1TWC5	1	1970.05	2503.65	1656.51	2263.72	1936.81	1594.36
G1TWM1	1	1750.73	1426.79	1751.32	1841.41	1369.59	2066.35
G1TWP4	19	6470.32	4928.07	6822.96	3930.92	3273.27	3624
G1TWQ3	3	1686.04	1797.28	1176.79	1530.01	1371.85	1570.61
G1TWT5	2	2170.06	1446.07	2097.65	1337.72	517.653	1513.24
G1TWU9	1	637.769	553.159	665.971	1225.58	505.275	1325.49
G1TX74	4	3149.86	1941.07	2574.21	1292.14	1264.4	1895.19
G1TXB1	1			204.591			543.183
G1TXP9	3	6780.85	5474.83	5094.66	3547.2		366.197
G1TY29	67	759593	691197	616966	643918	580732	622755
G1TY46	4	925.412	1507.93	456.607	1055.46	1100.98	652.016
G1TY57	2		1459.59	1097.36	2806.11	6833.73	2942.33
G1TY77	1	991.09	750.66	1444.33	506.742		558.228
G1TYC1	8	4568.79	6005.82	4552.84	4303.09	4004.24	3843.37
G1TYT3	5	14243.4	20524.8	11305.7	31959.4	31166.4	33866.5
G1TZA1	4	7128.96	19545.9	2306.63	19412.1	22511.5	24739.6
G1TZC1	14	3808.5	3920.6	3136.39	5376.3	5972.22	6032.08
G1TZG4	1	4388.63	2926.25	7319.04	2931.8		2745.67
G1TZK5	1	5520.62	5561.99	4944.04	2473.49	2282.76	3881.5
G1TZP0	22	42310	60583.3	36568.4	42610.7	48478.4	43641.2
G1U013	3	1414.4	1255.72	1070.92	1277.95	841.793	1046.34
G1U027	2	638.928	772.831	658.347	566.798	540.543	435.984
G1U0M5	23	3050.66	2766.25	2741.34	4794.78	4194.92	4410.68
G1U0Q7	14	8683.91	10428.1	8844.04	7053.23	5458.27	6225.63
G1U0Y6	5	3257.2	2057.26	1721.18	2023.53	1688.51	1585.35

G1U115	6	3258.93	4640.22	3119.7	3245.82	2189.38	4232.67
G1U128	13	40423.6	31948	33181.5	53225.2	56824.6	45623.3
G1U1B4	2	514.701	1002.88	550.641	1818.3	1607.52	1443.29
G1U1F6	3	2206.24	1267.26	1672.57	188.855	417.742	
G1U1Q1	15	6797.55	6666.55	8476.29	7409.29	6515.22	6977.62
G1U1Q8	1	2380.52	2469.2	1019.13	824.084		
G1U1T8	4	1569.39	2133.21	1560.8	2605.9	2044.55	2205.02
G1U2B5	1			318.718			513.797
G1U2E5	3	1497.91	1348.57	1182.6	985.233	895.657	776.843
G1U2M9	5	562.905	437.568	369.226	527.137	209.349	509.481
G1U2Q3	3	116040	137484	116926	178801	183128	176581
G1U2R2	33	4477.13	4477.13	4969.11	3057.02	3127.76	3200.43
G1U2S5	8	989.694	801.791	779.463	1299.58	1275.63	1581.66
G1U2V8	4	390095	521673	535170	814272	905871	883893
G1U2W0	2	1030.81	699.57	1307.39	1090.08	733.879	773.315
G1U2Y0	1	732.191	897.02	550.781	689.665	578.499	747.893
G1U334	80	15247.8	15761.2	9740.36	18388.7	19522.7	19717.8
G1U3C3	1	543.246	814.276		299.739	522.884	485.495
G1U3C5	9	5598.79	5074.04	6223.64	10369	8675.58	8106.25
G1U3I5	1		802.073	728.292	1219.78	1500.87	1128.87
G1U3U2	1	2948.05	2510.79	2795.92	6157.75	2847.56	4584.09
G1U3W5	1		597.623	1249.57	770.207		420.593
G1U410	9	43836.8	43405.1	63092.8	26776.2	21713	28113.6
G1U415	27	813692	813516	951591	875092	792785	862370
G1U453	2	678.288	1222.46	389.756	309.125		
G1U466	10	50026.6	64556.2	54961.1	38957.8	38338.3	39606.1
G1U492	13	4144.84	3464.65	3503.15	1742.48	1936.03	2184.7
G1U4H8	5	969.744	1543.66	1418.65	981.531	1631.98	1190.51
G1U4P8	11	2154.86	1903.56	2575.34	4621.1	3864.14	4372.05
G1U4V1	1		202.764	308.027	166.773	280.663	153.022
G1U522	6	914.637	811.182	1356.58	466.805	539.779	352.195
G1U571	1	540.583	522.63	763.306	501.874	342.537	160
G1U575	5	4453.87	6710.1	2787.19	5301.41	4215.44	7582.61
G1U5Q7	9	13783.9	14745.6	10124.6	10369.4	9781.27	9812.37
G1U602	3	1496.95	1956.6	1288.63	1743.82	1803.94	2086.09
G1U612	18	549356	458177	555590	326469	325245	296324
G1U647	2	1181.54	917.539	755.935	1090.17	1307.3	1544.99
G1U654	2	1197.85	1217.29	1392.78	2215.9	1172.12	1724.05
G1U685	1	1143.8	798.228	797.491	1903.5	1611.78	2694.75
G1U6A6	1		401.996				158.36
G1U6B2	12	16779.9	19622.1	17291.2	19004.9	19634.1	19784.7
G1U6C9	17	131038	95212.1	113302	189411	194126	207938
G1U6D0	10	2405.54	2594.84	2175.09	1774.49	1557.73	1772.78
G1U6R8	101	562833	610126	485507	946034	1.03E+006	1.00E+006
G1U6S4	12	7260.6	7714.22	7781.55	12840.7	10275.8	11742.4
G1U6T1	2		1066.66		1441.33	2392.55	1149.94
G1U754	34	384541	398051	317859	464671	429067	432305
G1U7Q3	2	1106.06	914.731	984.562			
G1U7Q6	2	535.39	567.235	422.933	956.607	563.214	796.67

G1U7S4	24	65929.6	84314.1	50523.2	3124.17	2863.89	2645.73
G1U7U3	3	1970.19	2948.88	3857.57	2417.09	3343.98	2627.87
G1U7V8	3	513.433	964.382	1078.22	347.688	826.353	859.33
G1U804	2	7470.18	3967.86	10380.7	5507	1850.36	5122.42
G1U8C4	11	4380.33	5499.18	3630.71	3017.79	3446.14	2009
G1U8F6	2	646.015	470.858	877.825	1631.7	838.443	1381.32
G1U8T9	7	6728.35	9139.09	3994.17	8658.18	8748.51	10096.1
G1U915	2	323.184	731.209	432.081	344.747	190.743	566.571
G1U949	7	2568.52	3777.56	1892.59	1266.22	1682.41	1419.2
G1U974	14	3506.9	2851.07	3187.25	3414.53	2876.57	3651.67
G1U985	18	2044.65	2250.15	1859.46	3299.13	3129.3	3273.69
G1U992	9	1925.93	1566	1968.09	1229.38	807.73	1205.4
G1U9C1	34	9998.65	8585.9	10751	5544.59	4953.94	5133.34
G1U9D6	1	5057.76	3546.66	5956.64	7285.22	4192.85	6742.51
G1U9I8	10	32007.5	25358.2	16643.9	30971.2	54637.7	98476.7
G1U9Q9	65	249748	233666	274724	259702	241981	282718
G1U9R4	400	701156	808023	485095	427412	445893	475324
G1U9S1	14	58805.5	55485.7	54740.4	33648	33648	43267.8
G1U9T1	32	10325.3	8713.89	11487.6	5395.69	4266.2	4892.9
G1U9U0	24	6710.76	6290.57	7842.14	3618.95	3163.7	3514.41
G1U9U2	15	138889	123981	122342	140166	149486	130318
O02767	8	960.556	1419.61	1233.07	2120.77	2614.28	2324.12
O18998	2		791.789		1912.56		1803.55
O19045	22	140560	111397	126054	99345.7	93016.1	96074.5
O19048	7	10757.2	9738.87	10219.9	7059.58	6697.27	7906.02
O19049	6	1190.29	1817.02	1353.73	1520.21	1712.69	1709.05
O19053	11	3165.29	4393.5	3242.09	4253.86	3977.46	4399.03
O46373	1		1778.83	4325.22			
O46559	8	2375.35	3052.95	2130.51	3193.76	3095.35	2874.09
O46649	2	435.028	202.565	392.226	878.286	529.669	734.474
O46650	15	787.673	853.411	817.56	5527.13	5959.3	5539.04
O62695	2	5153.35	5804.08	4272.91	8284.67	12415.7	9226.52
O62742	2	435.151	474.817	396.606	295.65		
O77622	31	6860.46	7093.3	9867.86	3880.77	3578.72	4030.95
O77676	12	3561.86	3446.8	2947.9	2728.78	2314.36	2765.21
O77708	2	1537.06	1254.07	1611.37	1434.26		843.516
O77768	2	1283.75	943.852	1386.33	1070.75	1646.2	1005.78
O77791	4	5062.36	6212.77	3306.44	6481.36	6189.57	5752.95
O77814	3	1492.04	1371.04	1758.79	2129.64	2045.63	1504.36
O77819	8	2243.97	1467.15	2684.74	2196.97	1525.54	1582.85
O77821	1	1576.22	821.517	1136.23	1350.34	823.31	1202.91
O97572	8	12089.9	12384.3	12147.7	9575.11	8812.38	9219.95
O97755	2	977.071	449.493	692.835	639.571	416.814	652.998
O97860	5	3183.07	2194	3220.38	3737.51	3146.47	3758.15
O97862	6	8324.78	10702.8	6992.24	11690.8	12668.7	12384.1
P00389	3	826.027	664.054	781.763		523.659	381.494
P00489	84	120737	121436	114300	6209.67	5949.59	5318.44
P00511	7	2122.54	2290.06	2329.78	1826.81	1382.52	1759.01
P00563	36	45429.8	65311.5	45579.2	6384.48	7216.31	7180.79

P00567	18	137301	158552	145304	67920.4	61520.2	62671.7
P00569	7	16039.8	14188.3	12040.1	9018.35	8769.29	8763.35
P00637	3	620.1	1278.01	1386.89	2047.65	873.023	1971.33
P00883	43	308982	339752	225519	5880.14	5461.25	5621.09
P00919	7	732.347	631.745	484.674	1241.28	1088.05	1304.71
P00939	2	901.426	601.114	562.374	419.716	713.104	426.624
P00949	50	37138.2	50448.3	25508.3	2997.21	3114.4	3011.54
P01376	1	4747.65	1761.76	6825.65	3665.58	2148.33	2753.27
P01377	7	132892	60499.6	251762	87167	111654	97650
P01683	1		569.948	640.374			1496.11
P01684	1	8312.85	5690.24	9341.14	11717.6	7599.71	13440
P01687	2	30101.4	32224	71773	44002.3	36396.5	44860.1
P01689	1	94474	68099.4	96385	92327.5	78168.5	112760
P01692	1	39953.8	26464	29388.1	39580.5	31531.7	45459.8
P01693	1	1886.47	1013.09	2262.03	3177.96	1118.41	3585.11
P01696	4	246418	192834	222204	298461	268889	291708
P01697	2	16560.2	23336.2	9866.97	24067.1	28865.4	27884.4
P01826	4	130505	104796	183579	152152	130065	153786
P01827	1	366.598	829.609	742.362	1117.19	1577.64	838.684
P01832	36	23792.6	21631.9	21514.2	40162.4	39133	41102.6
P01839	3	11346.5	16724.6	5642.57	34758.7	47648.6	30452.6
P01840	4	378500	475714	337016	576651	612775	515123
P01879	11	54010.8	41632.4	46904.9	105677	128868	104582
P01885	3	3768.35	4700.53	3471.94	5838.98	5303.97	6716.17
P01894	2	3682.35	2685.62	3751.04	5415.2	3613.77	4062.22
P01948	12	84459.1	109771	75614	70675.2	65659.7	70629.2
P02057	16	102807	159178	70201.9	95224.8	109166	87368.3
P02170	3	1386.75	1217.6	1576.16	2287.86	3356.23	1708.4
P02252	1	556.384	466.647	394.259	463.62	489.633	479.192
P02586	3	19177.6	24882.6	21348.8	964.867	838.635	884.144
P02591	3	5254.51	4914.31	4688			460.877
P02602	10	32247.8	30664.5	32075.2	5948.54	5861.36	5212.03
P02608	19	15900.9	21273.9	15795.7	3059.21	2886.94	2364.74
P02641	1	505.185	549.408	276.561			
P02643	8	24470.7	23351.9	22093.2	949.939	928.978	1157.7
P02742	10	195218	244975	203721	229332	214852	223125
P03988	25	1.36E+006	1.40E+006	1.15E+006	1.93E+006	2.30E+006	2.04E+006
P04191	4	490.765	735.74	592.672		1698.43	
P04461	13	3674.89	2842.24	3459.56	904.654		531.815
P05772	18	6960.49	8821.77	6454.39	6020.14	5340.12	6271.99
P06813	14	11377.7	12436.5	9403.5	8701.26	10466.9	8833.03
P06815	21	11451.6	12942.9	7360	9031.01	11148.5	8348.58
P06912	12	38472.3	25946.6	29933.3	37495.1	35255.1	40906.8
P07313	1			13256.5	11834.7		13248.3
P07466	5	169517	237157	189098	340506	381400	402814
P07467	3	101657	85009	104404	267076	174130	276533
P07468	4	15282.5	12253.9	16138.1	15497.5	12301.3	20788.6
P07469	1	509.094	277.832	466.131	944.758	485.318	1216
P07489	17	607984	542802	588772	893510	968385	952962

P07511	7	985.891	1116.28	1302.2	2439.56	1911.99	2494.38
P07855	1	727.469	988.077	332.644	475.828	800.706	570.148
P07952	9	6973.67	5642.21	6318.63	8384.73	6782.38	6452.19
P08507	13	10079.9	11209.5	10147.9	3046.69	2629.28	2629.91
P08628	5	1440.86	2504.24	831.933	1944.56	2055.48	1390.37
P08855	2	2331.17	2294.92	1860.67	3183.98	2835.7	2688.49
P09809	61	2.51E+006	1.69E+006	2.37E+006	2.65E+006	2.08E+006	2.40E+006
P10160	5	5325.65	4518.24	4167.91	2827.96	2553.94	2739.36
P11716	2	2125.05	699.836	2049.44	1027.7	629.844	
P11909	4	1498.52	1582.93	2700.58	1524.02	1357.56	1903.14
P11974	66	313436	302885	302574	33310.2	32294.7	31346.8
P12247	50	1.78E+006	1.94E+006	1.77E+006	1.37E+006	1.48E+006	1.64E+006
P12337	15	13387.3	17391.5	14673.1	18283.3	19048.2	20251.8
P12345	1			397249	473943		473921
P13019	3	3156.23	3359.92	4599.22	3337.41	4423.79	4267.88
P13280	6	1427.97	1792.93	1571.47	1688.03	1477.5	1702.48
P13491	11	10962.3	10406.4	11734.2	8511.6	8773.08	7393.01
P13642	14	4976.98	4073.14	5385.31	3788.63	3347.62	3745.46
P13806	23	3920.84	3769.25	4172.62	3911.64	3298.63	3495.09
P13834	2	1277.5	1297.92	1004.51	1118.33	530.748	925.036
P14422	3	9000.54	12856.3	10591.9	15898.8	11461.2	14963.9
P14755	12	8324.69	6736.47	7962.28	3943.1	4926.78	3496.62
P15122	5	6171.77	5731.48	5197.42	2641.57	1172.96	2315.25
P15196	27	231775	235234	228416	449296	455382	560385
P15253	20	13963.5	17596	13624.7	22043.1	19243.3	23409.1
P15285	4	7819.21	4439.62	11375.2	4004	3392.89	5110.99
P15541	2	530.434	422.064		643.724	384.687	376.347
P16258	3	867.288	1147.06	925.989	766.66	545.348	831.257
P16973	1	1794.68	1539.51	779.433	643.814	899.187	1248.81
P17165	8	102503	130396	56664.4	71086.1	73837.3	77876.2
P17454	16	166754	166854	173127	145222	136007	150379
P18287	26	728360	565638	651692	802596	689938	778259
P19007	21	162418	228645	123478	83013.7	91645.7	87622.3
P19134	59	210725	250556	152768	234081	272279	261122
P19761	8	15135.9	14085.3	15041.9	21698.4	20945.9	21786.8
P19943	3	1005.31	1178.06	1129.81	1731.92	1298.14	1666.47
P20058	26	112341	151657	75270.2	81956.2	97787.4	86381.8
P21195	22	10443.8	10362.5	9123.92	10286.6	8495.87	10428.6
P22000	1	555929	353336	488782	45332.3	37809.4	44288.3
P22687	37	99562.8	107804	93698.5	205050	194923	213329
P23035	14	184024	228609	131250	279437	286704	238991
P23108	11	282117	214887	300177	309443	305781	355951
P23612	27	20467	21036.1	20037	14408.8	12465.8	13039.2
P23775	13	86244.2	117446	66675.6	286585	258111	276713
P24480	1	1177.38	1295.5	1129.52	1366.03	2057.43	2009.55
P25227	11	22621.3	54892.5	11309.1	8200.15	10078.9	8294.11
P25704	39	53540.1	59783.4	57298.1	11596.9	11425.7	9967.38
P25915	1	476.577	357.567	449.645		416.07	349.993
P26202	7	21551.9	28531.9	14363.6	87590.3	106253	76945.5

P27115	2	944.936	1536.07	1527.44	1051.71	848.18	1316.35
P27124	19	3692.01	4083.47	3059.24	1830.17	1757.57	1628.13
P27170	30	2.46E+006	1.97E+006	2.16E+006	2.24E+006	2.46E+006	2.26E+006
P27821	3	1203.16	1266.01	1424.23	2257.53	1507.6	2081.86
P29294	25	14332.4	18493.1	11825.5	10687.7	9348.55	10275.8
P29338	7	4501.5	3467.54	3979.08	2091.95	2087.86	2424.07
P29562	17	8078.34	7254.12	7423.19	10634.9	9628.35	11514
P29678	10	2391.24	2819.99	3154.44	2193.02	1779.45	1978.67
P29694	15	19211.8	17492.7	20396	12200.7	11257.2	10994.5
P29751	38	1.07E+006	943574	963145	597215	500597	606778
P30801	3	1828.96	2716.54	1050.88	7195.71	5950.66	9353.58
P30946	43	42426.9	43462.9	51014.5	25918.9	22610.4	24308.8
P30947	13	13905.7	12029.8	16361.3	5414.52	5189.96	6692.21
P31097	1			415.624	649.3		321.774
P31347	3	2837.47	1047.72	2886.93	652.875	487.339	1332.57
P31429	4	1846.56	1763.3	1711.47	3533.25	2846.3	3495.13
P33047	4	87600.2	258749	81865.6	299350	456595	276770
P33477	2	899.645	927.643	881.912	923.217	921.468	1270.11
P33674	3	939.217	739.973	696.832	503.962	262.28	404.493
P33887	1	4296.93	3912.82	3812.22	3278.53	1831.36	3182.67
P34826	5	4159.54	3650.08	3983.82	2160.68	1642.85	2274.3
P35543	4	2808.37	2371.85	2404.76	1640.06	1221.75	1913.21
P35574	19	5566.36	4454.8	4469.45	1883.45	2038.1	1875.7
P35814	11	6161.69	5701.77	5462.26	5285.97	3779.89	3756.03
P36233	9	15413.7	14851.5	17792.9	38575.3	35975.5	40805.5
P37153	11	2.89E+006	1.55E+006	3.01E+006	1.19E+006	1.04E+006	1.19E+006
P40826	10	2443.71	2141.92	2864.27	2354.08	1791.66	2139.26
P41035	4	1163.96	1442.26	1418.69	1016.16	1063.92	1341.15
P41110	6	1975.95	1354.95	1272	932.72	1074.17	908.291
P41316	1		732.767	184.619			
P41975	9	51663.1	49744.3	40308	65214.5	69731.7	65884.8
P41982	3	1716.86	2946.34	1386.15	1973.16	1921.77	2492.68
P43348	5	4738.12	4695.7	4342.46	3269.13	2751.66	3163.45
P45701	19	6516.86	9047.17	5982.86	5924.85	6155.59	5296.56
P46406	25	90859.4	94005.5	108115	72372.7	71399.9	76805.4
P46409	20	36849.2	47329.5	22137.5	3851.75	4550.16	3302.12
P47814	4	1418.86	1617.22	2296.83	1317.09	1081.15	861.997
P47841	2	10543	8965.75	13339.2	27761.3	24582.6	31731.7
P47844	2	945.343	1119.55	1235.97	1291.08	1130.86	1227.53
P47845	5	552.748	935.465	448.558	1124.25	1494.83	1390.41
P47859	34	22435	21254	25593.9	12068.2	10927.3	10461.7
P48738	5	3114.32	2765.99	3339.54	1320.24	1259.44	1351.69
P48747	51	871943	746281	864811	940273	988292	980695
P49065	98	2.31E+006	1.63E+006	2.17E+006	1.65E+006	1.72E+006	1.62E+006
P49139	12	3099.73	3046.44	3656.27	1585.91	2195.51	2292.23
P50117	7	2240.48	2745.12	2419.94	2144.19	2217.87	2278.03
P50757	2	1336.05	1102.35	776.59	2319.85	1131.76	1203.43
P51662	5	1164.79	1113.86	450.99	666.026	351.837	559.029
P53614	11	920932	697696	871936	87780.3	58270.3	86215.9

P53787	5	9367.99	9923.55	10068.3	7415.54	6573.95	8552.4
P53789	42	368174	268039	478252	328212	291908	363657
P55057	10	609256	537744	759516	468664	389891	443890
P56201	15	9180.37	9247.63	6734.77	6519.96	6502.69	6947.2
P58772	26	11669.9	13427.6	10628.8	2706.3	4020.67	2987.59
P58776	19	10145.8	13033.8	7613.73	1800.84	1413.68	1859.34
P60990	1	363.339	445.112	258.224	424.569		105.959
P62139	16	3428.17	3654.98	3630.01	3620.49	3931.45	3073.31
P62143	4	2274.69	3332.58	2992.17	2079.73	3368.99	2336.54
P62160	9	14958.9	30412.9	15312.7	14114.7	14551.7	13046.3
P62493	14	28489.4	25969.3	23885.8	21553.1	17169.6	20953.1
P62497	5	5509.8	4533.54	4923.4	4836.85	3619.78	3410.22
P62740	2	11024.4	15069.5	8941.96	12833.7	8878.58	10020.4
P62943	1	808.347	2482.69	471.359		436.07	322.143
P62975	2	851.793	884.937	691.591	797.654	889.91	883.096
P63150	7	2095.79	2169.21	2598.85	1519.16	1688.78	1570.47
P63169	6	3848.21	3108.17	2818.01	2978.42	2039	2715.55
P67777	9	3803.39	3746.16	2909.67	3706.27	3125.13	2675.34
P68105	10	6625.88	6355.61	4796.6	6933.86	5932.89	6898.96
P68135	15	91452.1	105387	98348.1	18515.7	12729.8	15321.1
P79226	10	2877.05	5030.21	2185.23	1761.47	2768.97	2000.17
P79398	4	1707.8	1827.5	1310.9	1547.39	1666.85	1860.38
P80223	2	7749.22	7899.18	3824.35	40534.9	32331.4	19909.6
P80405	1	385.103		481.278			457.24
P80456	4	818.503	560.135	975.905	1154.53	782.533	1092.5
P82810	2	2041.21	2598.17	1943.43	1034.26	1286.9	1194.49
P83470	1	5521.6	10292.7	5154.1	10909	23116.3	12769.5
P84246	4	1208.35	692.68	1551.73	1776.74	1509.2	1613.34
P98118	19	73960.4	106235	60292	129923	137879	133494
P98136	29	153368	148317	112297	263352	235708	264620
P98137	35	189542	169552	164757	271517	257066	282009
P98139	11	25986.3	22173.3	24104.9	28259.5	28684.3	29661.3
Q01059	7	2515.46	2434.25	2798.66	1963.61	1540.95	2247.61
Q01971	8	4043.15	3919.74	3556.19	4345.79	3239.75	3730.78
Q02157	1	523.205	463.846		754.974	729.758	327.107
Q03505	21	4996.01	4504.16	5451.9	18478.7	15523.7	19350.8
Q09YN4	4	6054.83	9602.83	4371.36	5666.74	5887.39	6999.9
Q09YN5	2	2122.54	2377.43	2917.16	1249.69	841.367	1828.97
Q1XH18	6	1519.1	1051.31	1209.39			
Q28619	2	985.544	939.101	1095.14			
Q28623	3	973.277	665.91	862.017	1243.61	1236.67	828.488
Q28629	3	3419.96	1521.78	2853.01	2125.49	1993.61	2182.43
Q28632	1	262.217			827.661	760.024	796.716
Q28641	4	2185.14	2134.91	1803.75	762.706	883.172	1119.21
Q28651	1	1617.9	1119.4	1642.31	1510.9	695.537	1103.67
Q28653	3	1052.32	949.725	1021.91	894.149	788.395	1023.04
Q28661	17	118550	109093	146648	217175	172581	194309
Q28668	6	2820.09	2351.69	1802.75	2653.38	3009.15	2761.83
Q28679	11	159446	151913	125376	275994	238130	248234

Q28680	18	42311.3	43863.4	46807.5	33255.6	42857.6	32911.7
Q28685	5	3491.99	3625.78	2561.28	4119.91	4154.75	4191.78
Q28717	4	1185.3	1768.82	1236.89			
Q28719	5	1494.74	1727.84	1617.17	1756.3	1904.56	1716.29
Q28739	20	4353.06	3791.22	4686.28	9490.17	8235.79	9798.61
Q28740	2	1783.91	1653.3	1359.64	1767.88	1544.44	1610.35
Q28749	91	243896	296563	238910	617944	775639	659843
Q29425	6	1449.7	1268.28	1628.52	1176.04	738.015	871.161
Q29426	3	925.986	776.959	657.781	1264.05	1103.99	2792.69
Q29502	19	8499.3	6981.81	8939.11	5265.28	4498.73	5310.25
Q29504	30	6735.95	6835.32	7961.94	4047.39	3785.67	4022.81
Q29513	4	1298.81	735.755	717.378	4711.37	4633.83	5399.49
Q29514	1			785.094			
Q6XLQ7	2	4716.95	3548.73	3877.05	2427.7	1792.49	2596.59
Q7M2V4	10	4458.08	5397.2	5075.03	1182.09	702.706	919.093
Q7M370	3	5849.43	4267.07	6429.53	8876.22	6484.59	6019.65
Q866N2	5	5684.14	4309.97	5003.38	2785.5	2399.67	2651.18
Q8MI17	26	15858.9	17256.6	13124.6	16118.8	15807.2	16593.2
Q8MJF1	17	22646.5	30869.1	17819.4	16741	14661.8	16513.2
Q8MK67	2	1366.22	1119.15	1100.99	909.39	246.022	457.328
Q95209	11	1448.45	1708.52	1608.01	1963.43	1835.36	1953.53
Q95215	15	8304.73	8700.72	6983.32	10931.6	10096.9	10936.2
Q95222	2	1660.07	1283.01	1906.5	1025.93	597.885	1362.08
Q95223	14	7250.07	6814.58	6605.96	3469.29	3464.43	2667.7
Q95KM0	2		847.253	638.801	836.751	529.845	1525.42
Q95LB3	4	1883.91	1444.98	2074.07	5260.29	5758.79	5214.63
Q95MF9	14	92808.1	90103.4	102147	63451.6	51274.8	61036.5
Q9BDJ4	3	1067.66	988.898	748.878	1031.76	1042.82	1048.97
Q9BGN0	27	320362	343641	259688	593894	563496	603947
Q9GLY5	43	168944	219034	81324.5	104329	98606.6	95615.6
Q9MZS1	1			1056.49	4587.32	5110.75	
Q9N1E2	8	2077.3	2009.15	1907.69	1202.08	752.267	855.799
Q9TT38	32	9553.95	9738.05	9409.01	9553.98	8255.04	9528.68
Q9TTC6	12	21677.5	21633.7	13877.3	16595.7	18528.2	19019.9
Q9TU29	1	2146.25	3004.59	3713.09	1707.84	1571.46	1960.31
Q9TV70	6	4358.3	3856.04	4519.04	6050.27	5555.98	6063.8
Q9XS70	18	11448.3	11977.3	11816.6	6352.37	4921.24	7153.97
Q9XSC2	1	2736.55	3103.82	1800.04	1480.82	1714.45	
Q9XSC5	36	374024	361215	332865	483440	488290	497159
U3KM82	37	4439.91	4935.99	4262.17	3815	3640.57	3728.74
U3KMB5	13	173046	163102	168775	183741	160987	157007
U3KME3	5	1741.59	1282.32	2171.46	2045.17		1238.24
U3KMR2	16	205573	356344	113202	170256	248725	222164
U3KMU7	29	13225.7	12422.2	15924.3	10518	9714.94	10027.8
U3KN01	1	4287.15	5115.71	5864.93	3367.84	2474.63	4029.68
U3KN73	3	1010.49	1587.06	1281.19	1885.94	1533.44	1391.25
U3KN85	1	3512.19	2421.04	3426.77			
U3KNJ3	11	17488.4	15418.2	20381	16262.7	12561	14824
U3KNN0	7	2353.75	2222.15	2636.55	1540.54	1406.79	1760.73

U3KNW7	1				716.877	703.832	486.535
U3KNZ4	9	7312.52	9274.5	6877.44	5407.92	4507.74	5742.66
U3KP51	1				445.462	347.378	466.799
U3KP72	7	2751.76	3106.61	2749.61	6506.06	4689.9	4795.39
U3KPG6	9	2480.73	2509.38	2871.73	2950.66	2619.13	3090.12
U3KPI5	19	163259	160913	127174	160272	150693	162620
